# Diastereodivergent Construction of Octahydrophenanthridinone and Octahydrophenanthridine Cores

**DOI:** 10.3390/molecules30020371

**Published:** 2025-01-17

**Authors:** Chunzhao Sun, Hiromichi Nishikawa, Tsubasa Inokuma, Ken-ichi Yamada

**Affiliations:** 1 Graduate School of Pharmaceutical Sciences, Tokushima University, Shomachi 770-8505, Tokushima, Japan; 2 Research Cluster on “Key Material Development”, Tokushima University, Shomachi 770-8505, Tokushima, Japan; 3 Research Cluster on “Hybrid Modality Exploration”, Tokushima University, Shomachi 770-8505, Tokushima, Japan

**Keywords:** octahydrophenanthridinone, octahydrophenanthridine, alkaloid, stereodivergent synthesis, arylation, organocuprate, reduction

## Abstract

Diastereodivergent synthesis of octahydrophenanthridinone and octahydrophenanthridine skeletons, structural motifs often found in biologically active natural products, is described. We previously reported a total synthesis of a pancratistatin analog using novel octahydrophenanthridinone construction. In this study, we examined the generality of our method and its extension to octahydrophenanthridine formation. Conjugate addition of diarylcuprates to nitrosocyclohexenes, which were generated in situ from 2-chlorocyclohexanone oximes, provided 2-arylcyclohexanone oximes. Subsequent reduction of the oxime moiety gave *cis*- and *trans*-configured amines. Both amines were separately converted into the corresponding octahydrophenanthridinones and octahydrophenanthridines via hexahydrophenanthridine intermediates.

## 1. Introduction

**Figure 1:**
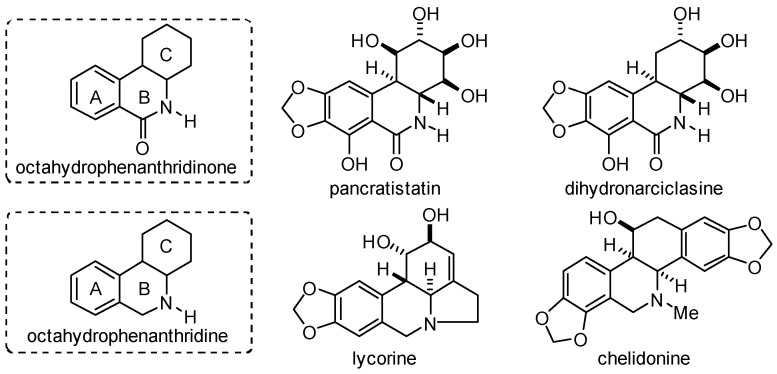
Examples of biologically active natural products with octahydrophenanthridinone and octahydrophenanthridine cores.

Octahydrophenanthridine and octahydrophenanthridinone are structural motifs often found in natural products such as pancratistatin, dihydronarciclasine, lycorine, and chelidonine (Figure 1) [1,2,3]. These natural products possess interesting biological activities such as antitumor activities [4,5] and antiviral activities [6,7]. Recently, derivatives of these natural products also possess potential activities as well as the parent natural products [8,9]. Therefore, the development of the methodologies for constructing these structures contributes not only to natural product synthesis but also to developing novel drug candidates derived from these natural products [10].

The representative strategy for octahydrophenanthridinone synthesis is initiated by amide formation of benzoic acid with cyclohexanamine bearing a leaving group at the β-position. Subsequent cyclization of the amide is accomplished via photo-induced cyclization [11], intramolecular nucleophilic substitution [12], and Heck reaction [13] to yield the desired structural cores. Another approach involves a coupling reaction of the arene and the cyclohexane moieties followed by lactamization. In this strategy, the first ring connection was realized by conjugated addition to cyclohexene [14,15,16,17,18,19], nucleophilic substitution to cyclohexane [20,21], or Suzuki–Miyaura coupling [22]. After introducing an amino group, the resulting coupling products were converted to octahydrophenanthridinones via lactam formation such as a Bischler−Napieralski reaction [23,24,25], formylation followed by oxidation [26], or ester–amide exchange [27]. An octahydrophenanthridine core was constructed by a Pictet–Spengler reaction of the same intermediate [14,28] as well as reduction of octahydrophenanthridinone [15,16,17] and hexahydrophenanthridine [29].

**Scheme 1:**
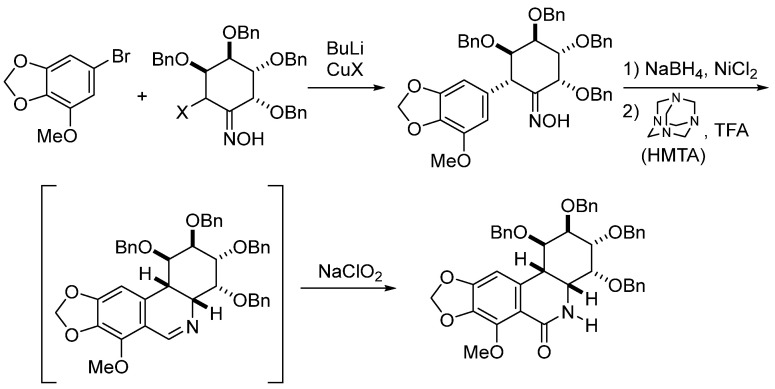
Our strategy for construction of an octahydrophenanthridinone core for synthesis of the pancratistatin analog.

During our synthetic study on a pancratistatin epimer [30], we established a novel strategy for the construction of an octahydrophenanthridinone core, involving coupling between aryl and cyclohexane rings by the reaction of diarylcuprate and α-acetoxy oxime, reduction of the oxime to give *cis*-amine, formylation using hexamethyleneteramine (HMTA) [31] followed by intramolecular imine formation, and oxidation of the imine to give lactam [32] (Scheme 1).

**Scheme 2:**
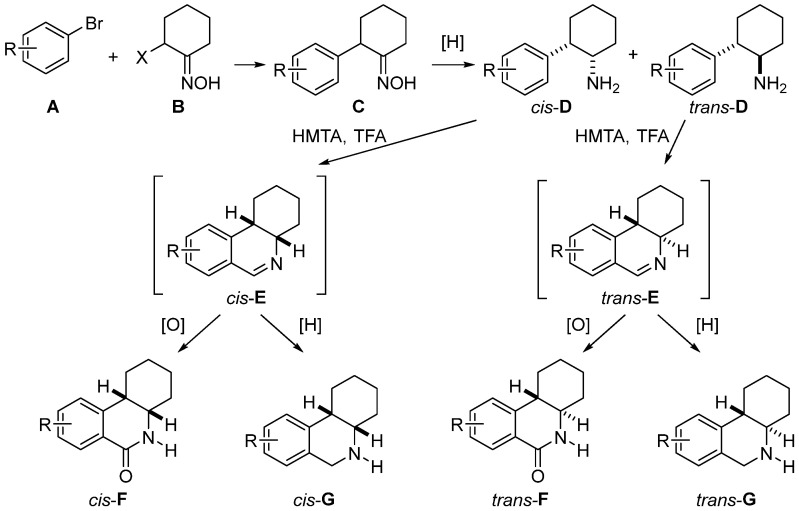
Concept of this work: Divergent synthesis of *cis*- and *trans*-tricyclic cores.

Based on this achievement, we envisioned that this strategy could be applied to the divergent synthesis of both octahydrophenanthridinone and octahydrophenanthridine (Scheme 2). First, the coupling of aryl bromide **A** and oxime **B**, bearing an α-leaving group gives α-aryl oxime **C** [33]. Subsequently, β-aryl amine **D**, generated by reduction of **C**, is converted into cyclic imine **E**. Oxidation and reduction of **E** would then afford oxazolidinone **F** and oxazolidine **G**, respectively. It is noteworthy that separation of *cis*- and *trans*-isomers of **D** makes both *cis*- and *trans*-isomers of **F** and **G** available. This would be a desirable feature for use in the drug discovery stage.

## 2. Results and Discussion

Our investigation commenced with the screening of copper salts for the coupling reaction of 5-bromobenzo[d][1,3]dioxole **1a** and 2-chlorocyclohexanone oxime **2** (Table 1). According to Weinreb’s report [34], **1a** was treated with butyllithium and cuprous iodide in tetrahydrofuran (THF) to prepare the corresponding organocuprate. The reaction of the cuprate and **2** provided 2-arylated oxime **3a** in 75% yield (entry 1). Although the use of cuprous acetate in place of iodide led to decreased yield (entry 2), the use of cuprous cyanide slightly improved the yield (entry 3). Finally, we found that cuprous 2-thiophenecarboxylate (CuTC) was the optimal copper source (entry 4). When diethyl ether was used as solvent instead of THF, the yield of **3a** significantly decreased to 9% (entry 5).

Next, we examined the generality of the reaction (Table 2). Trisubstituted aryl bromide **1b** was also converted to the corresponding arylated product **3b** in good yield (entry 1). Methoxyphenyl bromides **1c**–**e** gave products **3c**–**e** in moderate to good yields (entries 2–4). Furthermore, the reaction was applicable to aryl bromides such as **1f**–**h**, bearing a C1 unit at the *ortho*-positions (entries 5–7). Unfortunately, aryl bromides having an ester and an amide moiety failed to afford **3i** and **3j**, respectively, presumably due to the low nucleophilicity of the cuprates (entries 8 and 9).

We then explored reduction of 2-arylated oxime **3a** to prepare 2-arylcyclohexanamine **4a** (Table 3). Palladium-catalyzed hydrogenation, samarium(II)-mediated reduction, and reduction using DIBAL-H, LiB(*s*-Bu)_3_H, NaB(OAc)_3_H, and NaBH_3_CN resulted in either no reaction or the formation of unknown products (entries 1–6). Among the conditions tested, the reaction using sodium borohydride with nickel chloride [35,36] produced the desired amine **4a** as a 1:1 mixture of *cis*- and *trans*-isomers in moderate yields (entry 7). These diastereomers were readily separated by silica gel column chromatography. The assignment of *cis*- and *trans*-**4a** is based on the ^1^H and ^13^C NMR spectra of *cis*-**4a**, which are in good agreement with those reported [37]. The coupling constants between the adjacent benzylic and amino methine protons (3.0 and 11.0 Hz for *cis*- and *trans*-**4a**, respectively) are also consistent with the assignment. Interestingly, when the reduction was performed with molybdenum trioxide [36], the *trans*-isomer was preferentially produced with a 3:1 diastereomer ratio (entry 8). These results might suggest that the tortional strain and the steric repulsion between a hydride source and the aryl group are competitive in the reaction with nickel chloride, while the steric repulsion might be less severe with a hydride source derived from sodium borohydride and molybdenum trioxide.

We next applied the reduction with nickel chloride to obtain other amines with stereodivergency (Table 4). The results showed that substitution at *meta*- and *para*-positions of aryl groups was tolerated, and the reduction provided both diastereomers of the corresponding amines (entries 1–3), whereas *ortho*-substituted variants, such as **3e**–**h**, failed to give the desired amines, possibly due to the steric hindrance of the *ortho*-substituents (entries 4–7).

Next, the lactam formation of **4** to give octahydrophenanthridinone was carried out using HMTA followed by oxidation with sodium chlorite (Table 5). Both *cis*- and *trans*-**4a** were converted into the corresponding lactam **5a** in moderate yields (entries 1 and 2). The other substrates were successfully transformed into the corresponding tricyclic compounds except *cis*- and *trans*-**4d** (entries 3–6). The failed cyclization of *cis*- and *trans*-**4d** would be attributed to the electron deficiency at the *meta*-positions of the oxygen functionalities (entries 7 and 8).

**Scheme 3:**
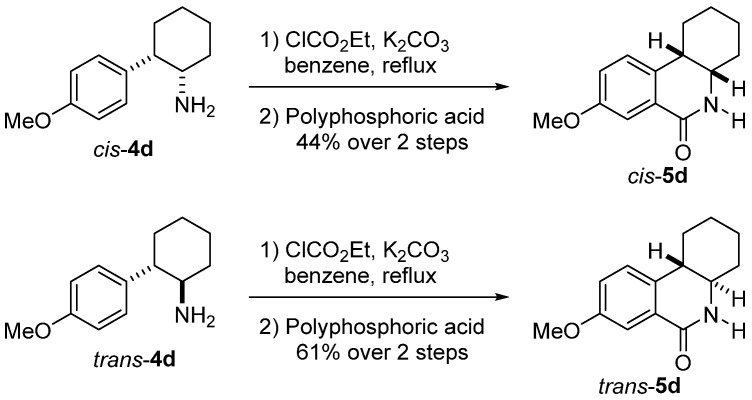
Construction of phenanthridinone from **4d** using a Bischler–Napieralski reaction.

The cyclization of **4d** was achieved using the Bischler–Napieralski reaction [38] (Scheme 3). Thus, *cis*- and *trans*-**4d** were separately converted to the corresponding ethyl carbamates, and the following reactions with polyphosphoric acid provided *cis*- and *trans*-**5d** in moderate yields.

**Scheme 4:**
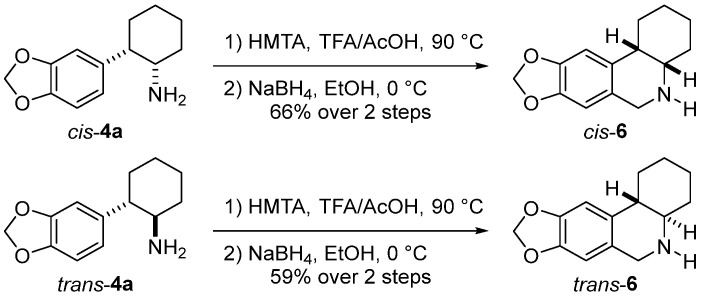
Reductive construction of phenanthridine from **4a**.

An octahydrophenanthridine skeleton was also constructed from **4a** via the imine intermediate (Scheme 4). Thus, *cis*- and *trans*-**4a** were separately converted into the corresponding imine intermediates using HMTA, and subsequent reduction with sodium borohydride in ethanol afforded *cis*-**6** and *trans*-**6** in good yields.

## 3. Conclusions

In conclusion, we successfully developed stereodivergent construction of octahydrophenanthridinone and octahydrophenanthridine cores via α-arylation of α-chloro oxime followed by reduction and cyclization. This protocol would enable divergent synthesis of these structural cores using various aryl bromide and cyclohexanone oximes.

## 4. Materials and Methods

### 4.1. General Information

All anhydrous reactions were carried out under a positive atmosphere of argon in dried glassware. Dehydrated solvents were purchased for the reactions and used without further desiccation. Analytical thin-layer chromatography was performed on Merck TLC silica gel 60F254 silica gel plates. Visualization was accomplished with molibudenium phosphate, *p*-anisaldehyde, Hannessian’s cocktail, or ninhydrin. Column chromatography was performed using Silica Gel 60N (particle size 0.040–0.050 mm) purchased from Kanto Chemical Co., Inc. (Tokyo, Japan). NMR spectra were recorded using a Bruker AV400N, Bruker AV500N (Bruker Corporation, Billerica, MA, USA) or JEOL JNM-ECZL500R (JEOL Ltd., Tokyo, Japan) in the stated solvents using tetramethylsilane as an internal standard. Chemical shifts were reported in parts per million (ppm) on the δ scale from an internal standard (NMR descriptions: s = singlet, d = doublet, t = triplet, q = quartet, m = multiplet, br = broad). Coupling constants, *J*, are reported in Hertz. Mass spectra were recorded on a Waters/Micromass SQD2, MICROMASS^®^ LCT PREMIERTM (ESI-TOF). Optical rotations were measured using a JASCO P-2200 polarimeter (JASCO Corp., Tokyo, Japan) (concentration in g·dL^−1^). IR was measured using a JASCO FT-IR 6200 (JASCO Corp., Tokyo, Japan). Melting point was determined on J-SCIENCE RFS-10. Unless otherwise noted, reagents were used without further purification. The substrates **1b** [39], **1f** [40], **1g** [41], **1h** [42], **1i** [43], **1j** [44], and **1k** [45] were prepared according to the literature procedures.

### 4.2. General Procedure for the Coupling Reaction of Aryl Bromide and α-Chloro Oxime

To a solution of bromoarene **1** (0.80 mmol) in dry THF (3 mL), a 1.60 M hexane solution of BuLi (0.50 mL, 0.80 mmol) was added dropwise at –78 °C, and the mixture was stirred for 30 min to give a solution of the corresponding organolithium. Then, the mixture was added to a suspension of CuTC (97 mg, 0.40 mmol) in dry THF (1 mL) at 0 °C via cannulation, and this mixture was stirred at 0 °C for 20 min to give a suspension of the corresponding diarylcuprate. The mixture was cooled to –78 °C, and to the mixture, a solution of **2** (0.20 mmol) in dry THF (0.5 mL + 0.25 mL wash) was added via cannulation. The mixture was warmed to 0 °C and stirred for 1 h. The reaction was quenched with saturated aqueous NH_4_Cl (5 mL), and the whole was extracted with EtOAc (3 × 10 mL). The combined organic layers were washed with brine, dried over Na_2_SO_4_, and concentrated under reduced pressure. The residue was purified by silica gel column chromatography (hexane/EtOAc 9:1 to 5:1) to give coupling product **3**.

2-(Benzo[d][1,3]dioxol-5-yl)cyclohexan-1-one Oxime (**3a**)

Isolated yield: 85%. Pale yellow solid of mp 119–122 °C. ^1^H NMR (500 MHz, CDCl_3_): δ 7.68 (br s, 1H), 6.75 (d, *J* = 8.0 Hz, 1H), 6.73 (d, *J* = 1.5 Hz, 1H), 6.67 (dd, *J* = 8.0, 1.5 Hz, 1H), 5.93 (s, 2H), 3.40 (dd, *J* = 9.5, 5.5 Hz, 1H), 2.97 (dt, *J* = 14.0, 5.0 Hz, 1H), 2.11 (m, 1H), 2.04–1.95 (m, 2H), 1.88–1.79 (m, 2H), 1.65–1.55 (m, 2H). ^13^C NMR (125 MHz, CDCl_3_): δ 162.1 (C), 147.5 (C), 146.0 (C), 134.4 (C), 121.1 (CH), 108.7 (CH), 108.1 (CH), 100.8 (CH_2_), 47.8 (CH), 33.4 (CH_2_), 25.7 (CH_2_), 24.7 (CH_2_), 23.9 (CH_2_). LRMS (ESI) (*m*/*z*): 232 [M–H]^−^. HRMS (ESI) (*m*/*z*): [M–H]^−^ calcd for C_13_H_14_NO_3_, 232.0974; found, 232.0967. IR (KBr): 3250, 2940, 2866, 1667, 1504, 1488, 1471, 1440, 1237, 1213, 1040, 725.

2-(7-Methoxybenzo[d][1,3]dioxol-5-yl)cyclohexan-1-one Oxime (**3b**)

Isolated yield: 83%. White solid of mp 151–154 °C. ^1^H NMR (400 MHz, CDCl_3_): δ 6.45 (s, 1H), 6.40 (s, 1H), 5.94 (s, 2H), 3.89 (s, 3H), 3.41 (dd, *J* = 9.0, 5.5 Hz, 1H), 3.00 (m, 1H), 2.15 (m, 1H), 2.04–1.95 (m, 2H), 1.90–1.79 (m, 2H), 1.65–1.60 (m, 2H). ^13^C NMR (125 MHz, CDCl_3_): δ 161.9 (C), 148.7 (C), 143.3 (C), 135.1 (C), 133.7 (C), 107.7 (CH), 102.4 (CH), 101.2 (CH_2_), 56.5 (CH_3_), 48.2 (CH), 33.5 (CH_2_), 25.6 (CH_2_), 24.8 (CH_2_), 24.0 (CH_2_). LRMS (ESI) (*m*/*z*): 262 [M–H]^−^. HRMS (ESI) (*m*/*z*): [M–H]^−^ calcd for C_14_H_16_NO_4_, 262.1079; found, 262.1089. IR (KBr): 3210, 2941, 2879, 2784, 1634, 1509, 1450, 1430, 1238, 1044.

2-(3-Methoxyphenyl)cyclohexan-1-one Oxime (**3c**)

Isolated yield: 71%. Pale yellow solid of mp 119–120 °C. ^1^H NMR (400 MHz, CDCl_3_): δ 7.23 (d, *J* = 8.0 Hz, 1H), 6.84 (d, *J* = 7.0 Hz, 1H), 6.81–6.75 (m, 2H), 3.80 (s, 3H), 3.49 (dd, *J* = 9.0, 5.0 Hz, 1H), 2.93 (m, 1H), 2.24 (m, 1H), 2.14–1.98 (m, 2H), 1.89–1.78 (m, 2H), 1.66–1.57 (m, 2H). ^13^C NMR (100 MHz, CDCl_3_): δ 162.2 (C), 159.5 (C), 142.3 (C), 129.2 (C), 120.6 (C), 114.2 (C), 111.7 (C), 55.1 (CH_3_), 47.8 (CH), 33.0 (CH_2_), 25.7 (CH_2_), 24.5 (CH_2_), 23.8 (CH_2_). LRMS (ESI) (*m*/*z*): 220 [M+H]^+^. HRMS (ESI) (*m*/*z*): [M+H]^+^ calcd for C_13_H_18_NO_2_, 220.1338; found, 220.1351. IR (KBr): 3201, 2931, 2833, 1662, 1609, 1583, 1488, 1446, 1262, 1051, 966, 926, 771, 745, 700.

2-(4-Methoxyphenyl)cyclohexan-1-one Oxime (**3d**)

Isolated yield: 73%. Pale yellow solid of mp 165–166 °C. ^1^H NMR (400 MHz, CDCl_3_): δ 7.15 (d, *J* = 9.0 Hz, 2H), 6.86 (d, *J* = 9.0 Hz, 2H), 6.71 (br s, 1H), 3.79 (s, 3H), 3.45 (dd, *J* = 8.0, 7.0 Hz, 1H), 3.01 (m, 1H), 2.22–2.12 (m, 1H), 2.06–1.98 (m, 2H), 1.88–1.80 (m, 2H), 1.64–1.56 (m, 2H). ^13^C NMR (100 MHz, CDCl_3_): δ 162.8 (C), 158.1 (C), 132.7 (C), 129.1 (Cx2), 113.7 (Cx2), 55.2 (CH_3_), 47.2 (CH), 33.4 (CH_2_), 25.8 (CH_2_), 24.7 (CH_2_), 23.9 (CH_2_). LRMS (ESI) (*m*/*z*): 220 [M+H]^+^. HRMS (ESI) (*m*/*z*): [M+H]^+^ calcd for C_13_H_18_NO_2_, 220.1338; found, 220.1328. IR (KBr): 3193, 2939, 2835, 1667, 1611, 1514, 1461, 1441, 1248, 1037, 970, 924, 841, 809, 778, 754.

2-(2-Methoxyphenyl)cyclohexan-1-one Oxime (**3e**)

Isolated yield: 84%. White solid of mp 186–187 °C. ^1^H NMR (400 MHz, CDCl_3_): δ 7.21 (d, *J* = 8.0 Hz, 2H), 6.96 (t, *J* = 8.0 Hz, 1H), 6.88 (t, *J* = 8.0 Hz, 1H), 3.87 (dd, *J* = 12.0, 4.0 Hz, 1H), 3.80 (s, 3H), 3.40 (m, 1H), 1.97–1.87 (m, 5H), 1.70–1.50 (m, 2H). ^13^C NMR (100 MHz, CDCl_3_): δ 162.0 (C), 156.7 (C), 129.4, (C), 128.5 (CH), 127.6 (CH), 120.4 (CH), 110.5 (CH), 55.5 (CH_3_), 41.4 (CH), 33.0 (CH_2_), 25.9 (CH_2_), 25.8 (CH_2_), 24.8 (CH_2_). LRMS (ESI) (*m*/*z*): 220 [M+H]^+^. HRMS (ESI) (*m*/*z*): [M+H]^+^ calcd for C_13_H_18_NO_2_, 220.1338; found, 220.1340. IR (KBr): 3267, 3064, 2998, 2938, 2845, 1714, 1671, 1598, 1492, 1461, 1324, 1240, 1132, 1047, 1031, 973, 930, 900, 756.

2-(2-(Triisopropylsilyloxymethyl)phenyl)cyclohexan-1-one Oxime (**3f**)

Isolated yield: 64%. White solid of mp 122–123 °C. ^1^H NMR (400 MHz, CDCl_3_): δ 7.40 (d, *J* = 8.0 Hz, 1H), 7.25–7.17 (m, 3H), 4.81 (d, *J* = 12.0 Hz,1H), 4.62 (d, *J* = 12.0 Hz, 1H), 3.76 (dd, *J* = 12.0, 4.0 Hz, 1H), 3.40 (d, *J* = 13.0 Hz, 1H), 2.04 (m, 1H), 1.99–1.89 (m, 3H), 1.75 (t, *J* = 13.0 Hz, 1H), 1.66–1.48 (m, 3H), 1.26–1.11 (m, 2H), 1.07 (d, *J* = 3.0 Hz, 9H), 1.05 (d, *J* = 3.0 Hz, 9H). ^13^C NMR (101 MHz, CDCl_3_): δ 161.9 (C), 152.0 (C), 138.7 (C), 127.6 (CH), 127.5 (CH), 127.1 (CH), 126.5 (CH), 63.8 (CH_2_), 43.9 (CH), 33.7 (CH_2_), 26.1 (CH_2_), 25.8 (CH_2_), 24.8 (CH_2_), 18.07 (CH_3_x3), 18.05 (CH_3_x3), 12.0 (CHx3). LRMS (ESI) (*m*/*z*): 388 [M+Na]^+^. HRMS (ESI) (*m*/*z*): [M+Na]^+^ calcd for C_22_H_37_NNaO_2_Si, 398.2491; found, 398.2491. IR (KBr): 3278, 3179, 3089, 2946, 2866, 2716, 1461, 1360, 1252, 1079, 1038, 1013. 993, 881, 747, 672, 638.

2-(2-(Benzyloxymethyl)phenyl)cyclohexan-1-one Oxime (**3g**)

Isolated yield: 66%. White solid of mp 149–150 °C. ^1^H NMR (400 MHz, CDCl_3_): δ 7.38–7.28 (m, 8H), 7.25–7.21 (m, 1H), 6.90 (br s, 1H), 4.55 (t, *J* = 12.0 Hz, 2H), 4.47 (d, *J* = 12.0 Hz, 1H), 4.42 (d, *J* = 12.0 Hz, 1H), 3.72 (dd, *J* = 12.0, 4.5 Hz, 1H), 3.38 (d, *J* = 15.0 Hz, 1H), 2.04 (m, 1H), 1.97–1.88 (m, 3H), 1.67 (m, 1H), 1.55–1.45 (m, 2H). ^13^C NMR (100 MHz, CDCl_3_): δ 162.4 (C), 140.1 (C), 138.4 (C), 135.6 (C), 129.7 (CH), 128.4 (CH), 128.2 (CH), 128.1 (CH), 127.9 (CH), 127.6 (CH), 126.5 (CH), 72.1 (CH_2_), 70.6 (CH_2_), 44.2 (CH), 33.8 (CH_2_), 26.0 (CH_2_), 25.7 (CH_2_), 24.7 (CH_2_). LRMS (ESI) (*m*/*z*): 348 [M+K]^+^. HRMS (ESI) (*m*/*z*): [M+K]^+^ calcd for C_20_H_23_KNO_2_, 348.1366; found, 348.1378. IR (KBr): 3223, 3062, 3036, 2925, 2867, 1493, 1450, 1410, 1360, 1102, 1071, 1022, 978, 934, 903, 745, 696.

2-(2-(Methoxymethyl)phenyl)cyclohexan-1-one Oxime (**3h**)

Isolated yield: 67%. White solid of mp 139–140 °C. ^1^H NMR (400 MHz, CDCl_3_): δ 7.34–7.29 (m, 3H), 7.23 (m, 1H), 6.73 (br s, 1H), 4.52 (d, *J* = 12.0 Hz, 1H), 4.32 (d, *J* = 12.0 Hz, 1H), 3.75 (dd, *J* = 12.0, 4.0 Hz, 1H), 3.46 (dt, *J* = 14.0, 3.5 Hz, 1H), 3.35 (s, 3H), 2.10–1.92 (m, 4H), 1.83 (m, 1H), 1.69–1.60 (m, 2H). ^13^C NMR (100 MHz, CDCl_3_): δ 162.3 (C), 139.8 (C), 135.7 (C), 129.3 (CH), 128.0 (CH), 127.9 (CH), 126.5 (CH), 73.1 (CH_2_), 58.0 (CH_3_), 44.3 (CH), 33.8 (CH_2_), 26.0 (CH_2_), 25.8 (CH_2_), 24.8 (CH_2_). LRMS (ESI) (*m*/*z*): 272 [M+K]^+^. HRMS (ESI) (*m*/*z*): [M+K]^+^ calcd for C_14_H_19_NO_2_K, 272.1053; found, 272.1047. IR (KBr): 3414, 3256, 2927, 2867, 2827, 1667, 1488, 1443, 1360, 1216, 1183, 1080, 975, 931, 903, 788.

### 4.3. General Procedure for the Reduction of α-Aryl Oxime

To a mixture of oxime **3** (0.80 mmol) and NiCl_2_·6H_2_O (400 mg, 1.68 mmol) in EtOH (10 mL), NaBH_4_ (250 mg, 6.61 mmol) was added portion-wise at 0 °C, and the mixture was stirred at room temperature. After 30 min, EtOAc (20 mL) and H_2_O (20 mL) were added to the mixture, and the whole was filtered through a Celite pad, which was successively washed with EtOAc (20 mL × 3). The combined washings were washed with H_2_O and brine, dried over Na_2_SO_4_, concentrated in vacuo, and purified by silica gel column chromatography (hexane/EtOAc 3:1, EtOAc, EtOAc/EtOH 1:2, and then EtOH) to give *cis*- and *trans*-**4**.

*cis-*2-(Benzo[d][1,3]dioxol-5-yl)cyclohexan-1-amine (*cis*-**4a**)

Isolated yield: 34%. Colorless oil. ^1^H NMR (400 MHz, CDCl_3_): δ 6.77 (d, *J* = 8.0 Hz, 1H), 6.73 (d, *J* = 1.5 Hz, 1H), 6.66 (dd, *J* = 8.0, 1.5 Hz, 1H), 5.93 (s, 2H), 3.20 (q, *J* = 3.0 Hz, 1H), 2.73 (dt, *J* = 12.5, 3.0 Hz, 1H), 1.93–1.81 (m, 3H), 1.71 (m, 1H), 1.65–1.49 (m, 3H), 1.36 (m, 1H), 1.16 (br s, 2H). ^13^C NMR (125 MHz, CDCl_3_): δ 147.6 (C), 145.7 (C), 138.8 (C), 120.2 (CH), 108.12 (CH), 108.05 (CH), 100.7 (CH_2_), 51.6 (CH), 47.1 (CH), 33.4 (CH_2_), 26.4 (CH_2_), 24.1 (CH_2_), 19.4 (CH_2_). LRMS (ESI) (*m*/*z*): 220 [M+H]^+^. HRMS (ESI) (*m*/*z*): [M+H]^+^ calcd for C_13_H_18_NO_2_, 220.1338; found, 220.1346. IR (NaCl): 3003, 2932, 2859, 1559, 1487, 1442, 1243, 1227, 775, 722.

*trans-*2-(Benzo[d][1,3]dioxol-5-yl)cyclohexan-1-amine (*trans*-**4a**)

Isolated yield: 37%. Colorless oil. ^1^H NMR (400 MHz, CDCl_3_): δ 6.76 (d, *J* = 7.5 Hz, 1H), 6.73 (d, *J* = 1.5 Hz, 1H), 6.68 (dd, *J* = 7.5, 1.5 Hz, 1H), 5.93 (s, 2H), 2.75 (td, *J* = 11.0, 3.5 Hz, 1H), 2.15 (td, *J* = 11.0, 3.5 Hz, 1H), 1.97 (m, 1H), 1.85–1.75 (m, 3H), 1.48–1.16 (m, 4H). ^13^C NMR (100 MHz, CDCl_3_): δ 147.8 (C), 146.0 (C), 138.6 (C), 120.9 (CH), 108.3 (CH), 107.7 (CH), 100.8 (CH_2_), 55.1 (CH), 53.6 (CH), 35.6 (CH_2_), 34.3 (CH_2_), 26.4 (CH_2_), 25.6 (CH_2_). LRMS (ESI) (*m*/*z*): 220 [M+H]^+^. HRMS (ESI) (*m*/*z*): [M+H]^+^ calcd for C_13_H_18_NO_2_, 220.1338; found, 220.1346. IR (NaCl): 3021, 2933, 1557, 1505, 1441, 1219, 775, 741.

*cis*-2-(7-Methoxybenzo[d][1,3]dioxol-5-yl)cyclohexan-1-amine (*cis*-**4b**)

Isolated yield: 44%. Pale yellow oil. ^1^H NMR (400 MHz, CDCl_3_): δ 6.43 (s, 1H), 6.38 (s, 1H), 5.94 (s, 2H), 3.90 (s, 3H), 3.22 (q, *J* = 2.0 Hz, 1H), 2.72 (dt, *J* = 10.0, 2.0 Hz, 1H), 1.96–1.80 (m, 3H), 1.71 (m, 1H), 1.64–1.48 (m, 3H), 1.36 (m, 1H). ^13^C NMR (100 MHz, CDCl_3_): δ 148.8 (C), 143.4 (C), 139.6 (C), 133.4 (C), 106.8 (CH), 101.6 (CH_2_), 101.2 (CH), 56.6 (CH_3_), 51.6 (CH), 47.4 (CH), 33.3 (CH_2_), 26.4 (CH_2_), 24.1 (CH_2_), 19.4 (CH_2_). LRMS (ESI) (*m*/*z*): 250 [M+H]^+^. HRMS (ESI) (*m*/*z*): [M+H]^+^ calcd for C_14_H_20_NO_3_, 250.1443; found, 250.1455. IR (NaCl): 3038, 3000, 2934, 2858, 1559, 1507, 1451, 1317, 1135, 1092, 825, 650.

*trans*-2-(7-Methoxybenzo[d][1,3]dioxol-5-yl)cyclohexan-1-amine (*trans*-**4b**)

Isolated yield: 44%. Pale yellow oil. ^1^H NMR (500 MHz, CDCl_3_): δ 6.42 (d, *J* = 1.0 Hz, 1H), 6.41 (d, *J* = 1.0 Hz, 1HH), 5.94 (s, 2H), 4.27 (br s, 2H), 3.90 (s, 3H), 2.85 (td, *J* = 10.5, 4.0 Hz, 1H), 2.32 (t, *J* = 10.5 Hz, 1H), 2.05 (m, 1H), 1.85–1.81 (m, 2H), 1.76 (m, 1H), 1.49–1.26 (m, 4H). ^13^C NMR (125 MHz, CDCl_3_): δ 149.2 (C), 143.6 (C), 137.7 (C), 133.9 (C), 107.4 (CH), 101.3 (CH_2_), 101.1 (CH), 56.5 (CH_3_), 55.0 (CH), 51.8 (CH), 33.9 (CH_2_), 33.5 (CH_2_), 25.9 (CH_2_), 25.2 (CH_2_). LRMS (ESI) (*m*/*z*): 250 [M+H]^+^. HRMS (ESI) (*m*/*z*): [M+H]^+^ calcd for C_14_H_20_NO_3_, 250.1443; found, 250.1445. IR (NaCl): 3049, 2994, 1556, 1511, 1449, 1222, 770.

*cis*-2-(3-Methoxyphenyl)cyclohexan-1-amine (*cis*-**4c**)

Isolated yield: 33%. Colorless oil. ^1^H NMR (500 MHz, CDCl_3_): δ 7.24 (t, *J* = 8.5 Hz, 1H), 6.81 (d, *J* = 8.5 Hz, 1H), 6.76 (s, 1H), 6.75 (d, *J* = 8.5 Hz, 1H), 3.80 (s, 3H), 3.25 (q, *J* = 2.5, 1H), 2.78 (dt, *J* = 12.5, 5.0 Hz, 1H), 1.95 (m, 1H), 1.91–1.82 (m, 2H), 1.73 (tt, *J* = 13.0, 4.0 Hz, 1H), 1.64–1.49 (m, 3H), 1.36 (m, 1H), 1.14 (br s, 2H). ^13^C NMR (125 MHz, CDCl_3_): δ 159.6 (C), 146.5 (C), 129.2 (CH), 120.0 (CH), 113.5 (CH), 111.1 (CH), 55.1 (CH_3_), 51.4 (CH), 47.5 (CH), 33.5 (CH_2_), 26.3 (CH_2_), 23.7 (CH_2_), 19.5 (CH_2_). LRMS (ESI) (*m*/*z*): 206 [M+H]^+^. HRMS (ESI) (*m*/*z*): [M+H]^+^ calcd for C_13_H_20_NO, 206.1545; found, 206.1554. IR (NaCl): 3155, 3035, 3000, 1558, 1465, 1381, 1227, 1197, 908, 739.

*trans*-2-(3-Methoxyphenyl)cyclohexan-1-amine (*trans*-**4c**)

Isolated yield: 31%. Colorless oil. ^1^H NMR (400 MHz, CDCl_3_): δ 7.23 (t, *J* = 7.5 Hz, 1H), 6.82 (d, *J* = 7.5 Hz, 1H), 6.78 (s, 1H), 6.76 (d, *J* = 7.5 Hz, 1H), 3.81 (s, 3H), 2.83 (td, *J* = 10.5, 3.0 Hz, 1H), 2.21 (td, *J* = 10.5, 3.5 Hz, 1H), 1.99 (m, 1H), 1.86–1.73 (m, 3H), 1.55–1.19 (m, 4H). ^13^C NMR (100 MHz, CDCl_3_): δ 159.8 (C), 146.0 (C), 129.6 (CH), 120.1 (CH), 113.6 (CH), 111.7 (CH), 55.1 (CH_3_), 54.9 (CH), 53.6 (CH), 35.1 (CH_2_), 34.0 (CH_2_), 26.3 (CH_2_), 25.5 (CH_2_). LRMS (ESI) (*m*/*z*): 206 [M+H]^+^. HRMS (ESI) (*m*/*z*): [M+H]^+^ calcd for C_13_H_20_NO, 206.1545; found, 206.1535. IR (NaCl): 3162, 2994, 2930, 2852, 1583, 1493, 1355, 1289, 1112, 821, 686, 652.

*cis*-2-(4-Methoxyphenyl)cyclohexan-1-amine (*cis*-**4d**)

Isolated yield: 33%. Colorless oil. ^1^H NMR (400 MHz, CDCl_3_): δ 7.13 (d, *J* = 9.0 Hz, 2H), 6.87 (d, *J* = 9.0 Hz, 2H), 3.80 (s, 3H), 3.21 (m, 1H), 2.76 (m, 1H), 1.98–1.80 (m, 3H), 1.71 (m, 1H), 1.65–1.43 (m, 3H), 1.42–1.31 (m, 1H). ^13^C NMR (100 MHz, CDCl_3_): δ 158.4 (C), 134.9 (C), 128.5 (CH), 114.1 (CH), 55.3 (CH_3_), 51.9 (CH), 45.4 (CH), 29.6 (CH_2_), 26.0 (CH_2_), 24.1 (CH_2_), 19.4 (CH_2_). LRMS (ESI) (*m*/*z*): 206 [M+H]^+^. HRMS (ESI) (*m*/*z*): [M+Na]^+^ calcd for C_13_H_19_NNaO, 228.1364; found, 228.1372. IR (KBr): 3346, 3253, 2935, 1739, 1670, 1657, 1512, 1251, 1033, 817.

*trans*-2-(4-Methoxyphenyl)cyclohexan-1-amine (*trans*-**4d**)

Isolated yield: 27%. White solid of mp 90–94 °C. ^1^H NMR (400 MHz, CDCl_3_): δ 7.14 (d, *J* = 9.0 Hz, 2H), 6.86 (d, *J* = 9.0 Hz, 2H), 3.80 (s, 3H), 2.79 (td, *J* = 10.5, 4.0 Hz, 1H), 2.20 (m, 1H), 2.00 (m, 1H), 1.84–1.74 (m, 2H), 1.51–1.21 (m, 4H). ^13^C NMR (100 MHz, CDCl_3_): δ 158.2 (C), 136.1 (C), 128.6 (CH), 114.0 (CH), 55.2 (CH_3_), 55.1 (CH), 52.2 (CH), 34.8 (CH_2_), 34.1 (CH_2_), 26.3 (CH_2_), 25.5 (CH_2_). LRMS (ESI) (*m*/*z*): 228 [M+Na]^+^. HRMS (ESI) (*m*/*z*): [M+Na]^+^ calcd for C_13_H_19_NNaO, 228.1363; found, 228.1364. IR (KBr): 3262, 2936, 2859, 1453, 1252, 1031.

### 4.4. General Procedure for the Formation of Phenanthridinones via Formylation and Oxidation

To a solution of **4** (0.15 mmol) in a 3:1 mixture of AcOH and TFA (2.4 mL) at room temperature, hexamethylenetetramine (112 mg, 0.75 mmol) was added. The mixture was heated at 90 °C with continuous stirring for 5 h and concentrated under reduced pressure. The residue was dissolved in MeOH (10 mL), and NaHCO_3_ (4.8 g) was carefully added. The mixture was then loaded onto a Celite pad, which was successively washed with EtOAc (20 mL × 3). The combined washings were washed with saturated aqueous NaHCO_3_ (20 mL) and brine (20 mL), dried over Na_2_SO_4_, and concentrated under reduced pressure. The residue was passed through silica gel column chromatography (hexane/EtOAc 1:2) to give the corresponding imine intermediate. To a solution of the imine in THF (1.1 mL), 2-methyl-2-butene (0.64 mL, 5.5 mmol), water (1.1 mL), NaH_2_PO_4_·2H_2_O (246 mg, 2.20 mmol), and NaClO_2_ (330 mg, 2.20 mmol) were added at 0 °C. The mixture was allowed to warm up to room temperature. After 35 min, saturated aqueous Na_2_SO_3_ (5 mL) was added, and the whole was extracted with EtOAc (10 mL × 3). The combined organic layers were washed with brine, dried over Na_2_SO_4_, and concentrated in vacuo. The residue was purified by column chromatography (EtOAc/hexane 1:1) to afford **5**.

*cis*-1,3,4,4a,5,11b-Hexahydro-[1,3]dioxolo [4,5-j]phenanthridin-6(2*H*)-one (*cis*-**5a**)

Isolated yield: 29%. White solid. Decomp. at >250 °C. ^1^H NMR (400 MHz, CDCl_3_): δ 7.52 (s, 1H), 6.64 (s, 1H), 5.99 (dd, *J* = 4.0, 1.5 Hz, 2H), 5.37 (br, 1H), 3.88 (dd, *J* = 7.0, 4.0 Hz, 1H), 2.69 (m, 1H), 1.80–1.70 (m, 2H), 1.65–1.50 (m, 4H), 1.40–1.30 (m, 2H). ^13^C NMR (100 MHz, CDCl_3_): δ 166.2 (C), 150.9 (C), 146.7 (C), 121.6 (C), 108.0 (CH), 106.7 (CH), 101.5 (CH_2_), 50.0 (CH_3_), 40.3 (CH), 30.2 (CH_2_), 29.2 (CH_2_), 24.5 (CH_2_), 19.8 (CH_2_). LRMS (ESI) (*m*/*z*): 268 [M+Na]^+^. HRMS (ESI) (*m*/*z*): [M+Na]^+^ calcd for C_14_H_15_NNaO_3_, 268.0950; found, 268.0958. IR (KBr): 3173, 3054, 2936, 2857, 1668, 1493, 1458, 1412, 1388, 1359, 1322, 1262, 1241, 1037, 934, 807, 741.

*trans*-1,3,4,4a,5,11b-Hexahydro-[1,3]dioxolo[4,5-j]phenanthridin-6(2*H*)-one (*trans*-**5a**)

Isolated yield: 45%. White solid of mp 191–192 °C. ^1^H NMR (400 MHz, CDCl_3_): δ 7.54 (s, 1H), 6.76 (s, 1H), 6.01 (s, 2H), 5.57 (s, 2H), 3.22 (td, *J* = 11.0, 4.0 Hz, 1H), 2.62 (td, *J* = 11.0, 4.0 Hz, 1H), 2.34 (d, *J* = 12.0 Hz, 1H), 1.98–1.83 (m, 3H), 1.55–1.25 (m, 4H). ^13^C NMR (100 MHz, CDCl_3_): δ 165.6 (C), 151.2 (C), 146.5 (C), 138.2 (C), 123.3 (C), 108.2 (CH), 103.9 (CH), 101.5 (CH_2_), 55.8 (CH_3_), 42.0 (CH), 32.2 (CH_2_), 27.1 (CH_2_), 25.4 (CH_2_), 24.1 (CH_2_). LRMS (ESI) (*m*/*z*): 268 [M+Na]^+^. HRMS (ESI) (*m*/*z*): [M+Na]^+^ calcd for C_14_H_15_NNaO_3_, 268.0950; found, 268.0950. IR (KBr): 3278, 3052, 2935, 2860, 1668, 1503, 1460, 1389, 1361, 1255, 1033, 930, 771.

*cis*-7-Methoxy-1,3,4,4a,5,11b-hexahydro-[1,3]dioxolo[4,5-j]phenanthridin-6(2*H*)-one (*cis*-**5b**)

Isolated yield: 31%. White solid of mp 189–190 °C. ^1^H NMR (400 MHz, CDCl_3_): δ 6.39 (s, 1H), 5.97 (d, *J* = 12.0 Hz, 2H), 5.34 (br s, 1H), 4.06 (s, 3H), 3.79 (dd, *J* = 6.5, 3.0 Hz, 1H), 2.60 (m, 1H), 1.82–1.65 (m, 6H), 1.39–1.24 (m, 2H). ^13^C NMR (100 MHz, CDCl_3_): δ 164.6 (C), 151.6 (C), 144.9 (C), 141.8 (C), 137.1 (C), 114.5 (C), 101.9 (CH), 101.4 (CH_2_), 60.8 (CH_3_), 49.1 (CH), 41.9 (CH), 29.7 (CH_2_), 28.6 (CH_2_), 24.6 (CH_2_), 19.6 (CH_2_). LRMS (ESI) (*m*/*z*): 298 [M+Na]^+^. HRMS (ESI) (*m*/*z*): [M+H]^+^ calcd for C_15_H_18_NO_4_, 276.1236; found, 276.1222. IR (KBr): 3549, 3480, 3411, 3050, 2941, 2898, 2878, 2842, 1670, 1474, 1388, 1357, 1322, 1217, 1061, 1040, 941, 807, 775.

*trans*-7-Methoxy-1,3,4,4a,5,11b-hexahydro-[1,3]dioxolo[4,5-j]phenanthridin-6(2*H*)-one (*trans*-**5b**)

Isolated yield: 27%. White solid of mp 204–205 °C. ^1^H NMR (400 MHz, CDCl_3_): δ 6.51 (s, 1H), 5.98 (d, *J* = 12.0 Hz, 2H), 5.62 (br s, 1H), 4.06 (s, 3H), 3.10 (td, *J* = 12.0, 4.0 Hz, 1H), 2.50 (td, *J* = 12.0, 4.0 Hz, 1H), 2.28 (d, *J* = 12.0 Hz, 1H), 1.97–1.87 (m, 3H), 1.50–1.20 (m, 5H). ^13^C NMR (125 MHz, CDCl_3_): δ 164.0 (C), 151.6 (C), 144.6 (C), 139.9 (C), 136.7 (C), 116.0 (C), 101.4 (CH_2_), 99.0 (CH), 60.8 (CH_3_), 54.6 (CH), 42.9 (CH), 31.7 (CH_2_), 27.4 (CH_2_), 25.3 (CH_2_), 24.0 (CH_2_). LRMS (ESI) (*m*/*z*): 298 [M+Na]^+^. HRMS (ESI) (*m*/*z*): [M+H]^+^ calcd for C_15_H_18_NO_4_, 276.1236; found, 276.1235. IR (KBr): 3182, 3060, 2926, 2855, 1729, 1663, 1509, 1475, 1363, 1325, 1278, 1217, 1136, 1088, 1035, 927, 797, 769, 727, 630.

*cis*-9-Methoxy-1,3,4,4a,5,10b-hexahydrophenanthridin-6(2*H*)-one (*cis*-**5c**)

Isolated yield: 47%. White solid of mp 172–178 °C. ^1^H NMR (500 MHz, CDCl_3_): δ 8.02 (d, *J* = 9.0 Hz, 1H), 6.85 (dd, *J* = 9.0, 2.5 Hz, 1H), 6.69 (d, *J* = 9.0 Hz, 1H), 5.83 (br s, 1H), 3.91 (q, *J* = 3.0 Hz, 1H), 3.85 (s, 3H), 2.76 (m, 1H), 1.82 (m, 1H), 1.76–1.55 (m, 5H), 1.44–1.24 (m, 2H). ^13^C NMR (125 MHz, CDCl_3_): δ 166.6 (C), 162.7 (C), 146.1 (C), 130.3 (CH), 120.4 (C), 112.1 (CH), 111.8 (CH), 55.3 (CH_3_), 50.0 (CH), 40.4 (CH), 30.1 (CH_2_), 29.0 (CH_2_), 24.5 (CH_2_), 19.8 (CH_2_), 19.9 (CH_2_). LRMS (ESI) (*m*/*z*): 254 [M+Na]^+^. HRMS (ESI) (*m*/*z*): [M+H]^+^ calcd for C_14_H_18_NO_2_, 232.1338; found, 232.1324. IR (KBr): 3179, 2948, 1732, 1658, 1604, 1497, 1254, 816.

*trans*-9-Methoxy-1,3,4,4a,5,10b-hexahydrophenanthridin-6(2*H*)-one (*trans*-**5c**)

Isolated yield: 50%. White solid of mp 206–209 °C. ^1^H NMR (500 MHz, CDCl_3_): δ 8.04 (d, *J* = 9.0 Hz, 1H), 6.85 (dd, *J* = 9.0, 2.0 Hz, 1H), 6.79 (d, *J* = 2.0 Hz, 1H), 6.12 (br s, 1H), 3.86 (s, 3H), 3.27 (td, *J* = 11.0, 4.0 Hz, 1H), 2.67 (td, *J* = 11.0, 4.0 Hz, 1H), 2.39 (d, *J* = 12.0, 1H), 2.11–1.85 (m, 4H), 1.59–1.24 (m, 3H). ^13^C NMR (125 MHz, CDCl_3_): δ 166.0 (C), 162.9 (C), 144.2 (C), 130.2 (CH), 122.0 (C), 111.1 (CH), 109.6 (CH), 55.6 (CH), 55.3 (CH_3_), 42.1 (CH), 32.2 (CH_2_), 26.7 (CH_2_), 25.4 (CH_2_), 24.1 (CH_2_). LRMS (ESI) (*m*/*z*): 254 [M+Na]^+^. HRMS (ESI) (*m*/*z*): [M+Na]^+^ calcd for C_14_H_17_NNaO_2_, 254.1157; found, 254.1154. IR (KBr): 3179, 3060, 2925, 1734, 1664, 1610, 1394, 1032, 803.

### 4.5. General Procedure for the Formation of Phenanthridinones via a Bischler–Napieralski Reaction

To a solution of **4d** (0.055 mmol) in benzene (1.0 mL), anhydrous potassium carbonate (12 mg, 0.082 mmol) and ethyl chloroformate (0.015 mL, 0.14 mmol) were added, and the mixture was heated under reflux for 1 h. Filtration through filter paper and removal of volatile materials in vacuo gave a thick oil, which solidified with the addition of hexane. Recrystallization from hexane/EtOAc (4:1) gave the corresponding carbamate intermediate. A mixture of the carbamate and PPA (200 mg) was heated at 110 °C for 1.5 h. After cooling to room temperature, water (5 mL) was added, and the mixture was filtered through filter paper. The filtrate was concentrated in vacuo, and the residue was purified by column chromatography (EtOAc/hexane 1:1) to afford **5d**.

*cis*-8-Methoxy-1,3,4,4a,5,10b-hexahydrophenanthridin-6(2*H*)-one (*cis*-**5d**)

Isolated yield: 44%. White solid of mp 177–178 °C. ^1^H NMR (400 MHz, CDCl_3_): δ 7.60 (d, *J* = 2.5 Hz, 1H), 7.11 (d, *J* = 8.0 Hz, 1H), 7.03 (dd, *J* = 8.0, 2.5 Hz, 1H), 5.60 (br, 1H), 3.90 (m, 1H), 3.85 (s, 3H), 2.77 (m, 1H), 1.80–1.32 (m, 8H). ^13^C NMR (125 MHz, CDCl_3_): δ 166.6 (C), 158.6 (C), 136.3 (C), 128.5 (C), 127.9 (CH), 120.1 (CH), 111.1 (CH), 55.5 (CH_3_), 50.2 (CH), 39.3 (CH), 30.3 (CH_2_), 29.7 (CH_2_), 29.2 (CH_2_), 19.9 (CH_2_). LRMS (ESI) (*m*/*z*): 254 [M+Na]^+^. HRMS (ESI) (*m*/*z*): [M+Na]^+^ calcd for C_14_H_17_NNaO_2_, 254.1157; found, 254.1161. IR (KBr): 3051, 2930, 2895, 1737, 1666, 1493, 1453, 1390, 1321, 1270, 1250, 1037, 804, 771.

*trans*-8-Methoxy-1,3,4,4a,5,10b-hexahydrophenanthridin-6(2*H*)-one (*trans*-**5d**)

Isolated yield: 61%. White solid of mp 194–195 °C. ^1^H NMR (400 MHz, CDCl_3_): δ 7.55 (d, *J* = 3.0 Hz, 1H), 7.12 (d, *J* = 8.0 Hz, 1H), 6.98 (dd, *J* = 8.0, 3.0 Hz, 1H), 5.85 (br s, 1H), 3.78 (s, 3H), 3.19 (td, *J* = 12.0, 4.0 Hz, 1H), 2.59 (td, *J* = 12.0, 4.0 Hz, 1H), 2.35 (dd, *J* = 12.0, 3.0 Hz, 1H), 1.92–1.78 (m, 3H), 1.51–1.21 (m, 4H). ^13^C NMR (125 MHz, CDCl_3_): δ 165.9 (C), 158.5 (C), 134.5 (C), 130.1 (C), 124.6 (CH), 119.5 (CH), 111.4 (CH), 55.8 (CH_3_), 55.5 (CH), 41.5 (CH), 32.1 (CH_2_), 26.9 (CH_2_), 25.4 (CH_2_), 24.2 (CH_2_). LRMS (ESI) (*m*/*z*): 254 [M+Na]^+^. HRMS (ESI) (*m*/*z*): [M+H]^+^ calcd for C_14_H_18_NO_2_, 232.1338; found, 232.1334. IR (KBr): 3191, 3066, 2968, 2872, 2833, 1668. 1494, 1429, 1357, 1313, 1261, 1030, 877, 801, 774, 724, 673.

### 4.6. General Procedure for the Formation of Phenanthridines via Formylation and Reduction

To a solution of **4a** (0.22 mmol) in a 3:1 mixture of AcOH and TFA (3.6 mL) at room temperature, hexamethylenetetramine (151 mg, 1.07 mmol) was added. The mixture was heated at 90 °C with continuous stirring for 5 h and concentrated under reduced pressure. The residue was dissolved in MeOH (10 mL), and NaHCO_3_ (5.4 g) was carefully added. The mixture was then loaded onto a Celite pad, which was successively washed with EtOAc (20 mL × 2). The combined washings were washed with saturated aqueous NaHCO_3_ (20 mL) and brine (20 mL), dried over Na_2_SO_4_, and concentrated under reduced pressure. The residue was passed through silica gel column chromatography (hexane/EtOAc 1:2) to give the imine intermediate. To a solution of the imine in EtOH (3.6 mL), NaBH_4_ (22.6 mg, 0.598 mmol) was added at 0 °C, and the mixture was stirred at the same temperature. After 10 min, the mixture was diluted with EtOAc and H_2_O, and the whole was extracted with EtOAc (20 mL × 2), dried over Na_2_SO_4_, and concentrated in vacuo. The residue was purified by column chromatography (CHCl_3_/MeOH 20:1 to 5:1) to afford **6**.

*cis*-1,2,3,4,4a,5,6,11b-Octahydro-[1,3]dioxolo[4,5-j]phenanthridine (*cis*-**6**)

Isolated yield: 66%. Yellow oil. ^1^H NMR (500 MHz, CDCl_3_): δ 6.55 (s, 1H), 6.47 (s, 1H), 5.88 (d, *J* = 1.5 Hz, 1H), 5.87 (d, *J* = 1.5 Hz, 1H), 4.04 (d, *J* = 16.0 Hz, 1H), 3.96 (d, *J* = 16.0 Hz, 1H), 3.07 (dd, *J* = 7.0, 3.5 Hz, 1H), 2.48 (dt, *J* = 12.0, 3.0 Hz, 1H), 1.84 (m, 1H), 1.75–1.67 (m, 3H), 1.61–1.50 (m, 2H), 1.48–1.35 (m, 2H). ^13^C NMR (125 MHz, CDCl_3_): δ 145.7 (C), 145.6 (C), 134.1 (C), 127.9 (C), 108.5 (CH), 105.7 (CH), 100.5 (CH_2_), 52.1 (CH), 49.1 (CH_2_), 39.2 (CH), 31.9 (CH_2_), 31.4 (CH_2_), 25.9 (CH_2_), 20.5 (CH_2_). LRMS (ESI) (*m*/*z*): 232 [M+H]^+^. HRMS (ESI) (*m*/*z*): [M+H]^+^ calcd for C_14_H_18_NO_2_, 232.1338; found, 232.1324. IR (NaCl): 3031, 2930, 1503, 1482, 1292, 1256.

*trans*-1,2,3,4,4a,5,6,11b-Octahydro-[1,3]dioxolo[4,5-j]phenanthridine (*trans*-**6**)

Isolated yield: 59%. White solid of mp. 86–89 °C. ^1^H NMR (500 MHz, CDCl_3_): δ 6.78 (s, 1H), 6.47 (s, 1H), 5.89 (d, *J* = 1.0 Hz, 1H), 5.88 (d, *J* = 1.0 Hz, 1H), 4.12 (d, *J* = 17.0 Hz, 1H), 3.94 (d, *J* = 17.0 Hz, 1H), 2.41 (td, *J* = 11.0, 3.5 Hz, 1H), 2.34–2.27 (m, 2H), 1.93–1.81 (m, 3H), 1.46–1.28 (m, 3H), 1.17 (m, 1H). ^13^C NMR (125 MHz, CDCl_3_): δ 146.1 (C), 145.5 (C), 131.8 (C), 128.7 (C), 106.0 (CH), 105.6 (CH), 100.6 (CH_2_), 59.2 (CH), 49.1 (CH_2_), 43.9 (CH), 33.9 (CH_2_), 29.8 (CH_2_), 26.3 (CH_2_), 25.4 (CH_2_). LRMS (ESI) (*m*/*z*): 232 [M+H]^+^. HRMS (ESI) (*m*/*z*): [M+Na]^+^ calcd for C_14_H_17_NNaO_2_, 254.1157; found, 254.1149. IR (NaCl): 3315, 2924, 2854, 1631, 1579, 1485, 1258, 1229, 1039, 797.

## Figures and Tables

**Table 1:** Optimization of the coupling reaction ^[a]^.

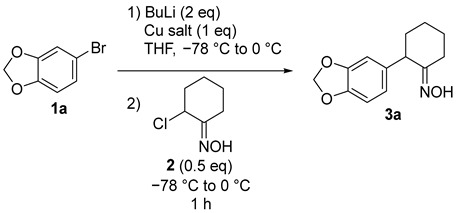		
Entry	Cu Salt	Yield (%) ^[b]^
1	CuI	75
2	CuOAc	59
3	CuCN	80
4	CuTC	85
5 ^[c]^	CuTC	9

^[a]^ The reactions were performed using **1a** (0.80 mmol). ^[b]^ Isolated yield. ^[c]^ Et_2_O was used instead of THF.

**Table 2:** Substrate scope of the coupling reaction ^[a]^.

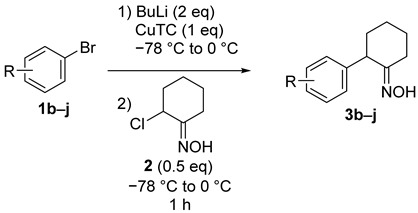			
Entry	1	3	Yield (%) ^[b]^
1	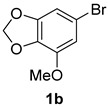	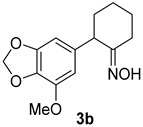	83
2	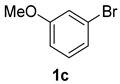	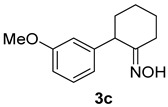	71
3	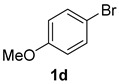	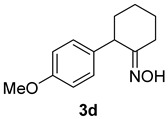	73
4	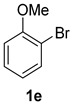	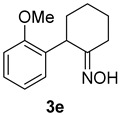	84
5	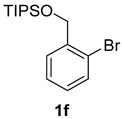	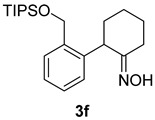	64
6	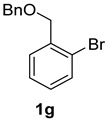	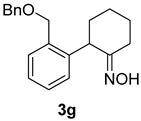	66
7	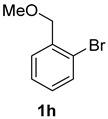	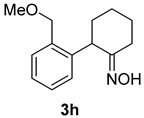	67
8	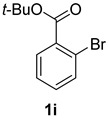	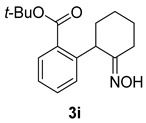	0
9	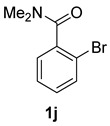	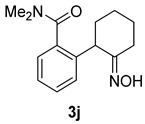	0

^[a]^ The reactions were performed using **1** (0.80 mmol). ^[b]^ Isolated yield.

**Table 3:** Screening of the conditions for reduction of **4a** ^[a]^.

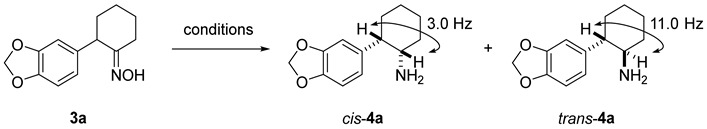				
Entry	Conditions	Yield of 4a (%) ^[b]^		Comment
		*cis*	*trans*	
1	H_2_ (1 atm), Pd(OH)_2_/C, EtOAc/MeOH	0	0	no reaction
2	SmI_2_, THF	0	0	complex mixture
3	DIBAL-H, THF	0	0	no reaction
4	LiB(*s*-Bu)_3_H, THF	0	0	no reaction
5	NaB(OAc)_3_H, THF	0	0	no reaction
6	NaBH_3_CN, TFA	0	0	complex mixture
7	NiCl_2_·6H_2_O, NaBH_4_, EtOH	34	37	
8	MoO_3_, NaBH_4_, MeOH	17	51	

^[a]^ The reactions were performed using **3a** (0.20 mmol). ^[b]^ Isolated yield.

**Table 4:** Substrate scope of reduction of **3** ^[a]^.

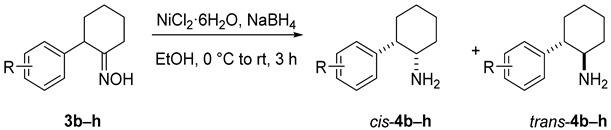				
Entry	3	4	Yield (%) ^[b]^	
			*cis*-4	*trans*-4
1	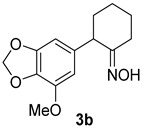	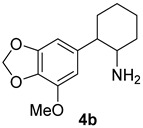	44	44
2	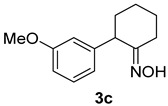	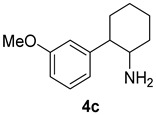	33	31
3	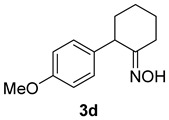	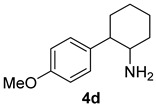	33	27
4	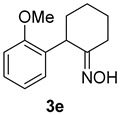	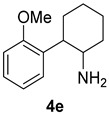	0	0
5	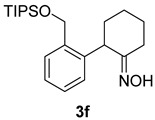	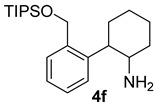	0	0
6	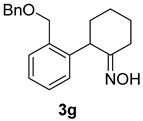	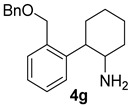	0	0
7	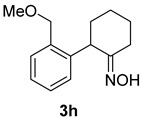	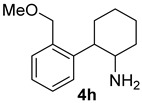	0	0

^[a]^ The reactions were performed using **3** (0.2 mmol). ^[b]^ Isolated yield.

**Table 5:** Substrate scope of phenanthridinone formation ^[a]^.

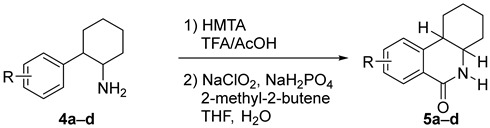			
Entry	4	5	Yield (%) ^[b]^
1	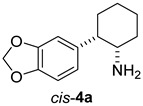	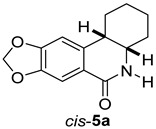	29
2	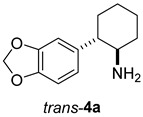	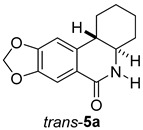	45
3	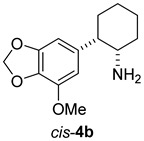	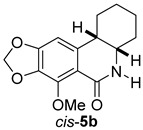	31
4	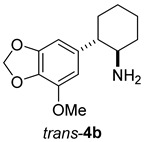	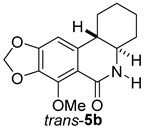	27
5	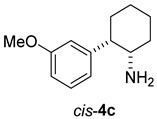	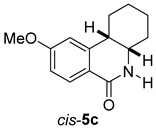	47
6	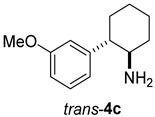	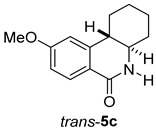	50
7	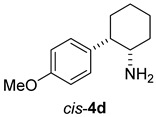	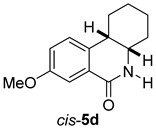	0
8	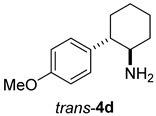	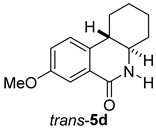	0

^[a]^ The reactions were performed using **4** (0.1 mmol). ^[b]^ Isolated yield over 2 steps.

## Data Availability

The data that support the findings of this study are available in the manuscript and Supplementary Materials of this article.

## Supplementary Materials

The following supporting information can be downloaded at: https://www.mdpi.com/article/10.3390/molecules30020371/s1: The copies of ^1^H- and ^13^C-NMR spectra of **3a**–**h**; and *cis*- and *trans* **4a**–**d**, **5a**–**d**, and **6** are available online.
