# Supplementary material for: Diastereodivergent Construction of Octahydrophenanthridinone and Octahydrophenanthridine Cores
Source: Molecules. 2025 Jan 17;30(2):371. doi: 10.3390/molecules30020371 (PMC11767715; doi:10.3390/molecules30020371)
Supplement: Supplementary file 1 [file molecules-30-00371-s001.zip › molecules-3423266-supplementary.pdf]

Supplementary Materials

Diastereodivergent Construction of Octahydrophenanthridinone and Octahydrophenanthridine Cores

Chunzhao Sun,[1] Hiromichi Nishikawa,[1] Tsubasa Inokuma,[1,2,3] and Ken-ichi Yamada[1,2,*]

[1] Graduate School of Pharmaceutical Sciences, Tokushima University, Shomachi, Tokushima 770-8505, Japan

[2] Research Cluster on "Key Material Development", Tokushima University, Shomachi, Tokushima 770-8505, Japan.

[3] Research Cluster on "Hybrid Modality Exploration", Tokushima University, Shomachi, Tokushima 770-8505, Japan.

yamak@tokushima-u.ac.jp

Contents

Copies of ^{1}H and ^{13}C NMR spectra S2–27

1. Copies of ^{1}H and ^{13}C spectra

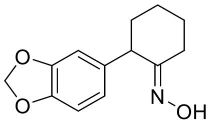

3a

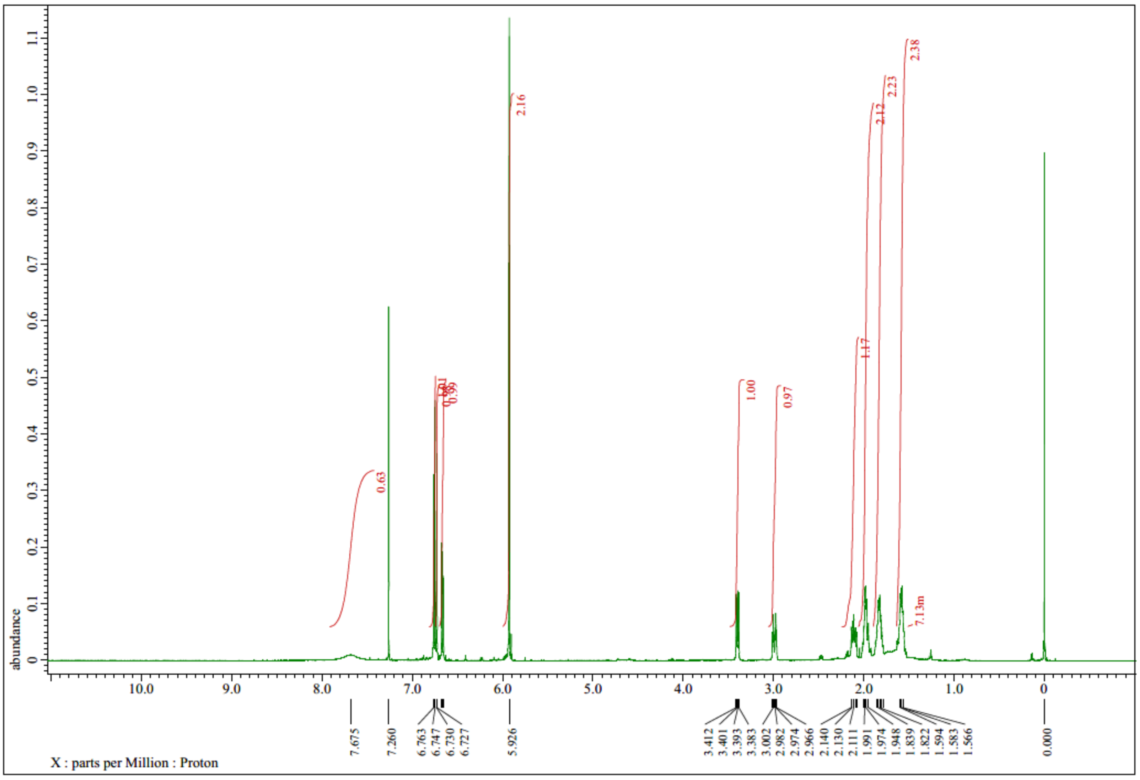

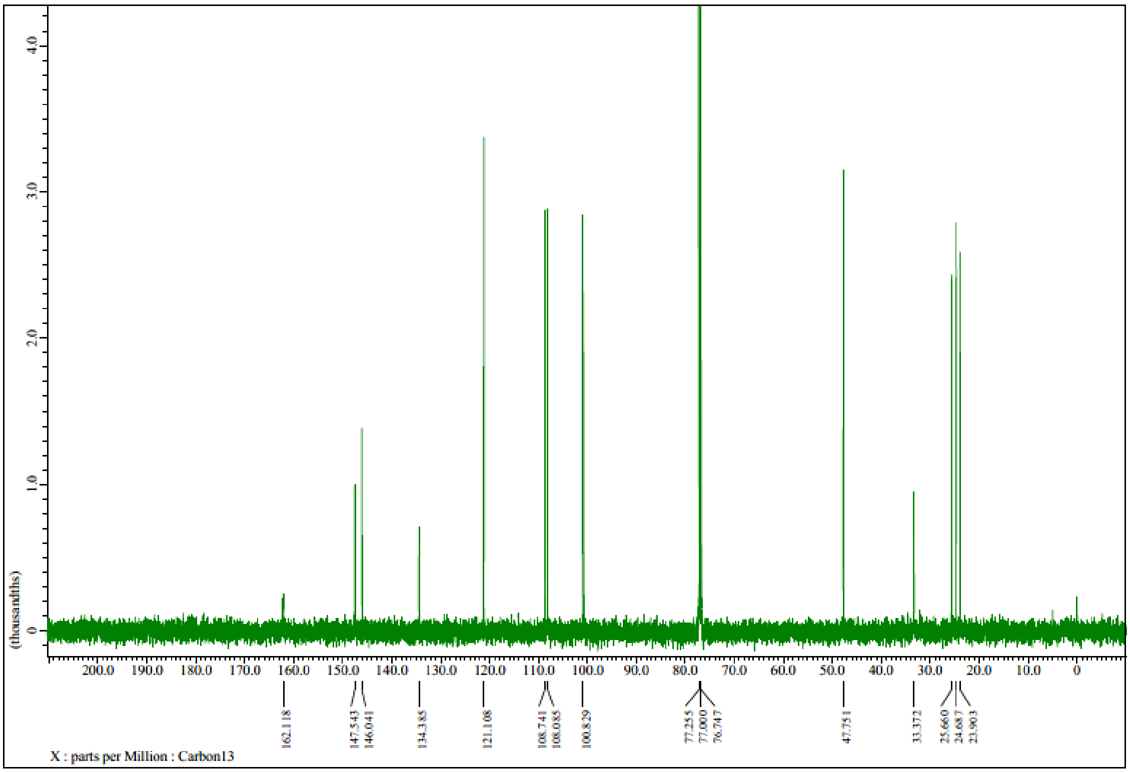

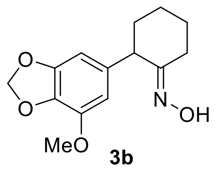
O
O
MeO
N
OH

3b

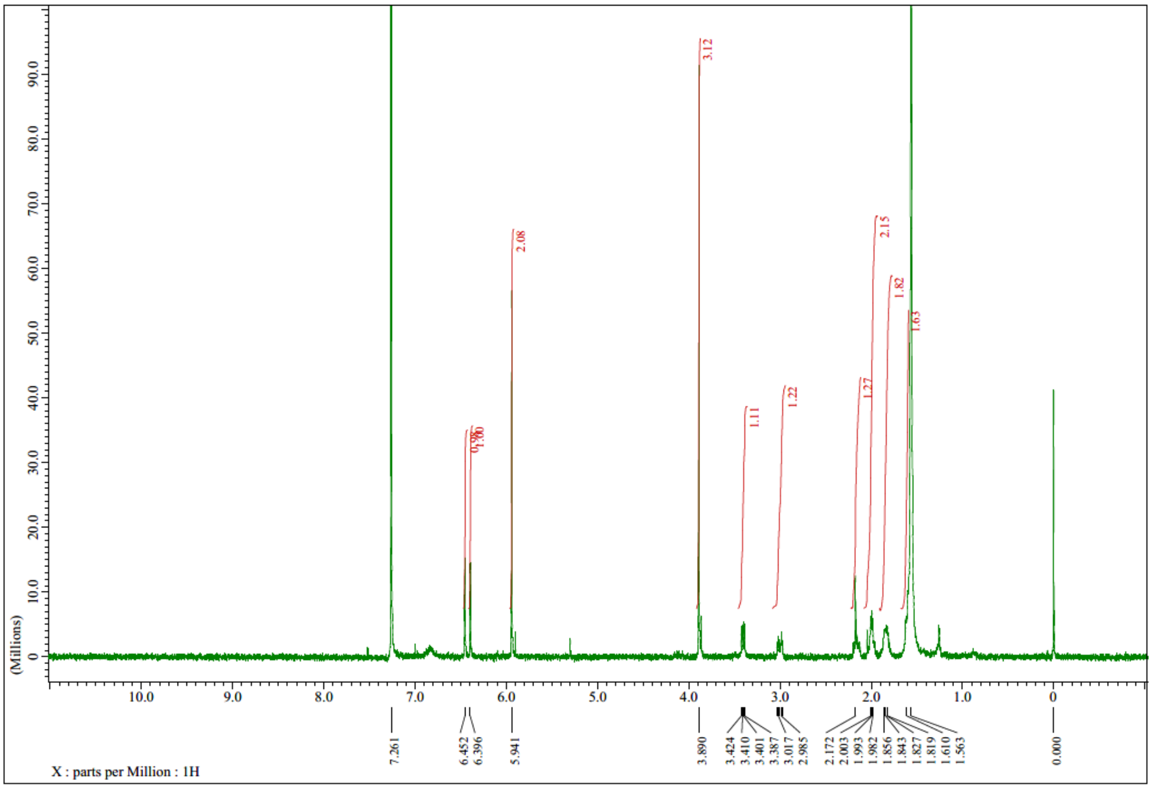
X : parts per Million : 1H
(Millions)
7.261
6.452
6.396
5.941
3.890
3.424
3.410
3.401
3.387
3.017
2.985
2.172
2.003
1.993
1.982
1.856
1.843
1.827
1.819
1.610
1.563
0.000
3.12
2.08
2.15
1.82
1.63
1.27
1.22
1.11
0.98
1.00

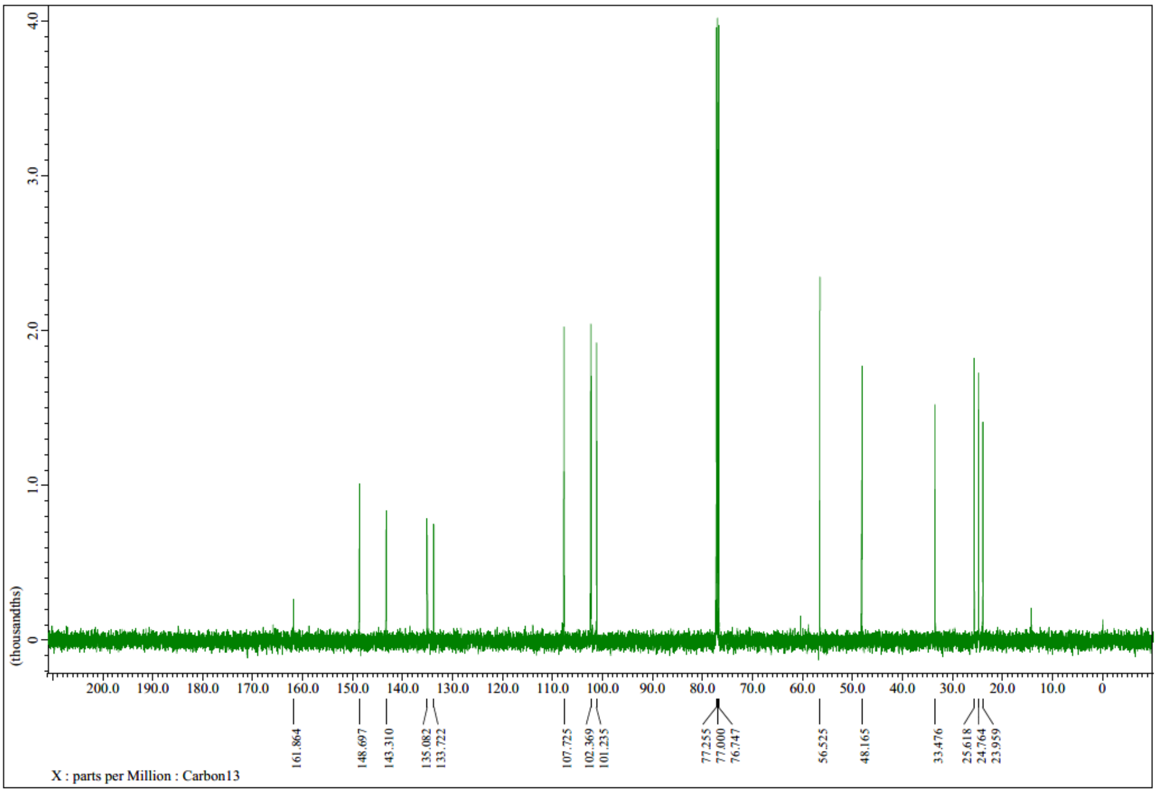
X : parts per Million : Carbon13
(thousandths)
161.864
148.697
143.310
135.082
133.722
107.725
102.369
101.235
77.255
77.000
76.747
56.525
48.165
33.476
25.618
24.764
23.959

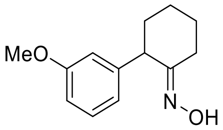
MeO
N
OH

3c

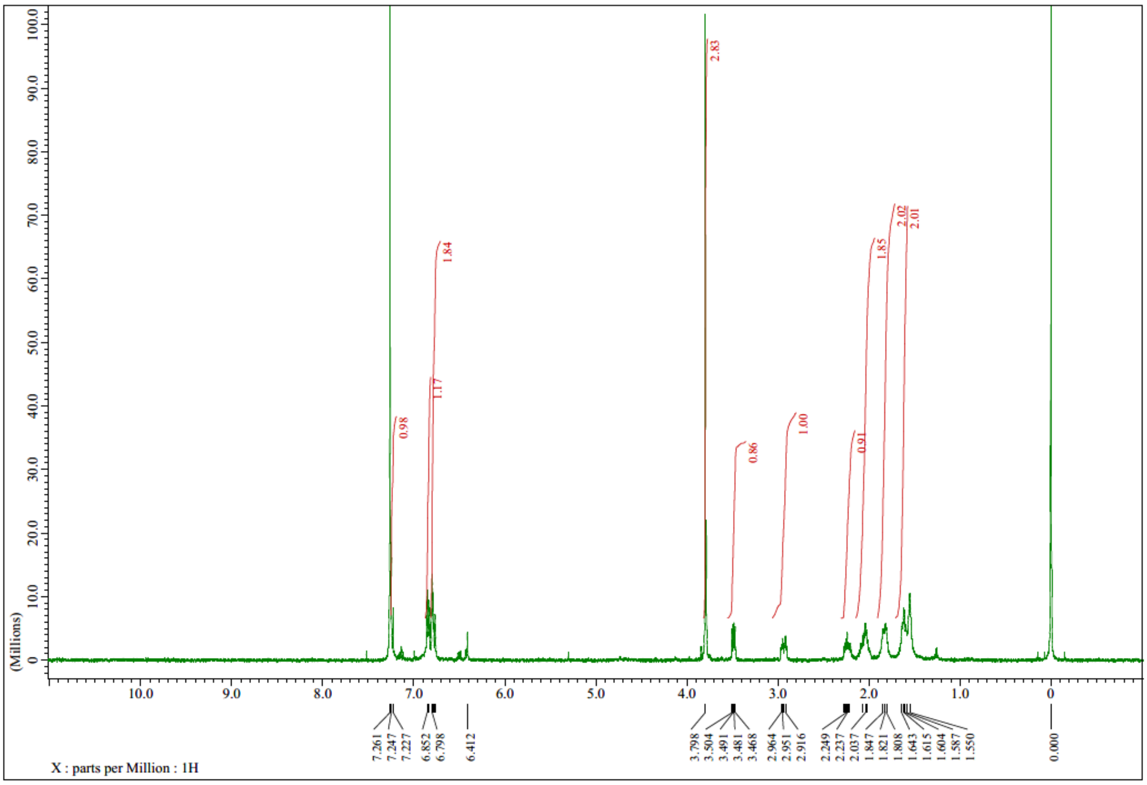
X : parts per Million : 1H
(Millions)

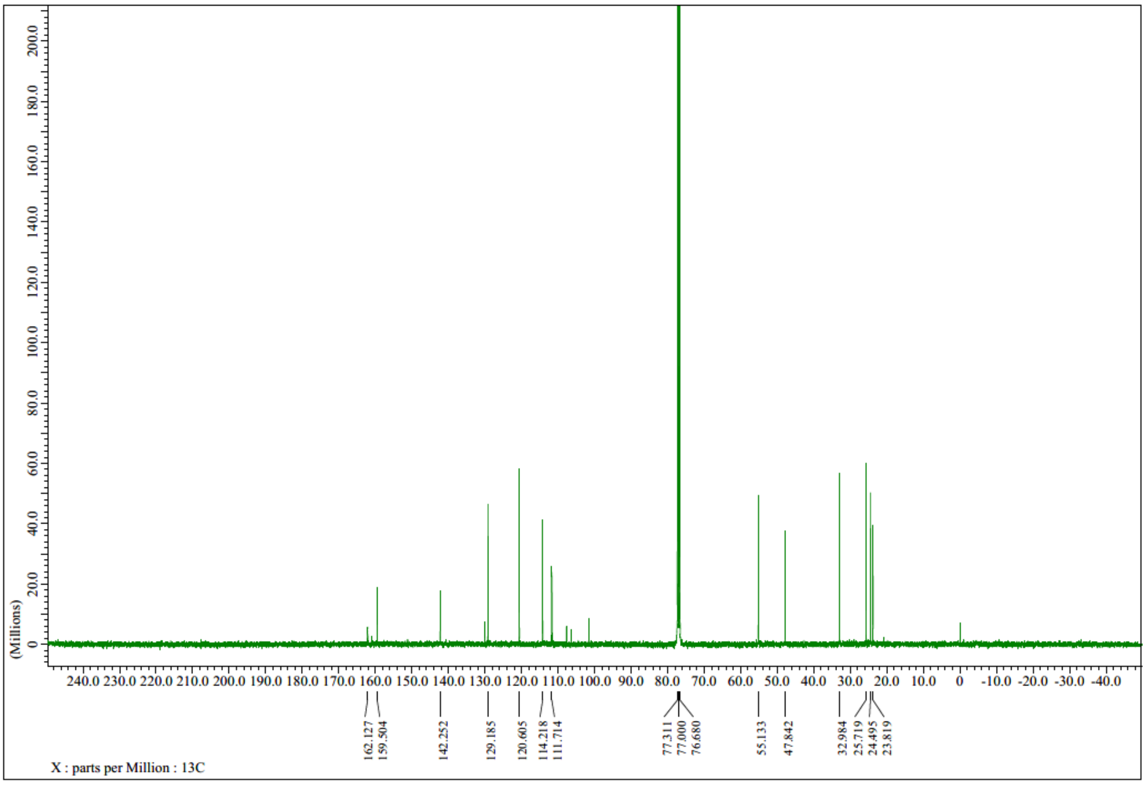
X : parts per Million : 13C
(Millions)

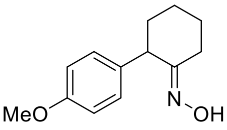
MeO
N
OH

3d

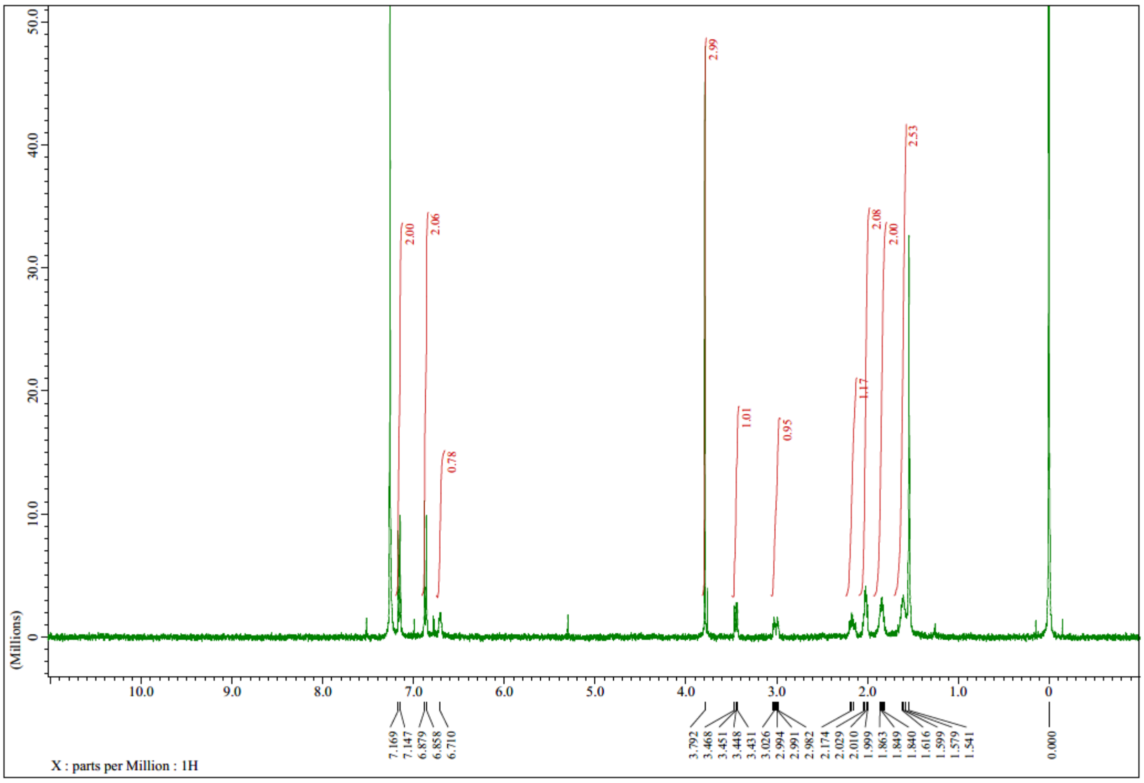
X : parts per Million : 1H
(Millions)

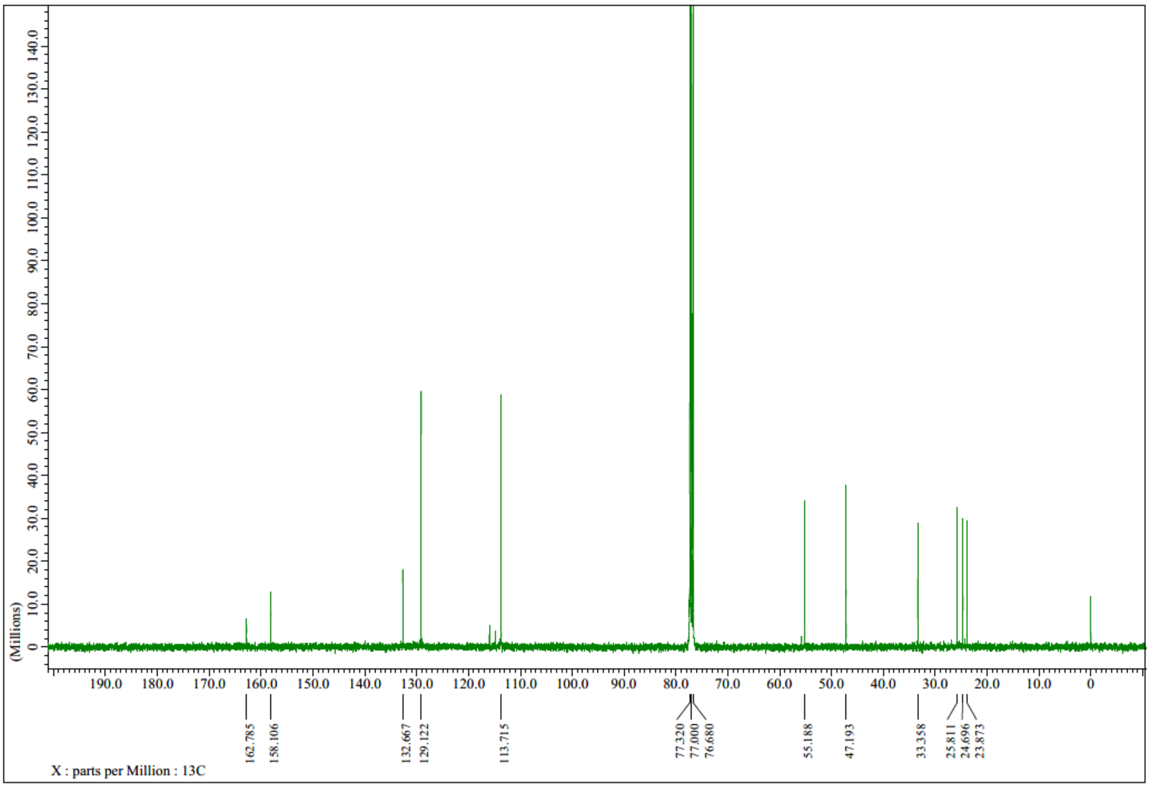
X : parts per Million : 13C
(Millions)

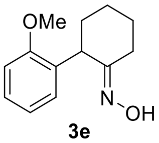
OMe
N
OH

3e

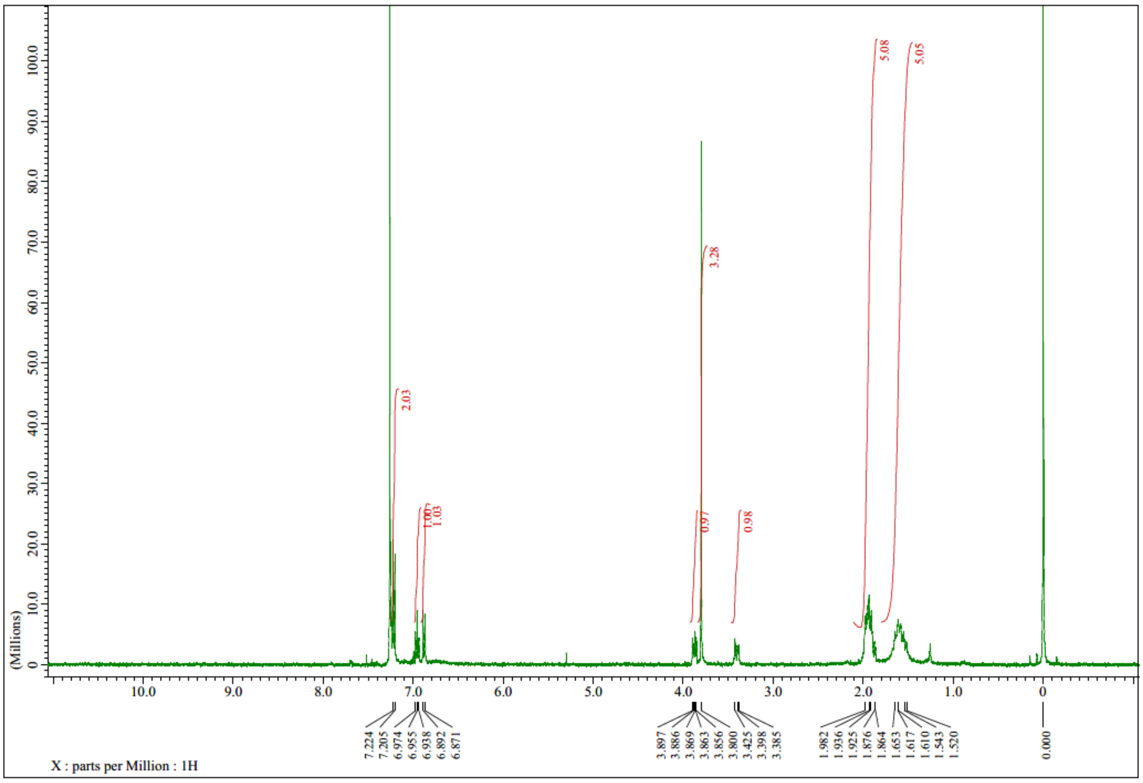
(Millions)
X : parts per Million : 1H
2.03
1.00
1.03
0.97
3.28
0.98
5.08
5.05
7.224
7.205
6.974
6.955
6.938
6.892
6.871
3.897
3.886
3.869
3.863
3.856
3.800
3.425
3.398
3.385
1.982
1.936
1.925
1.876
1.864
1.653
1.617
1.610
1.543
1.520
0.000

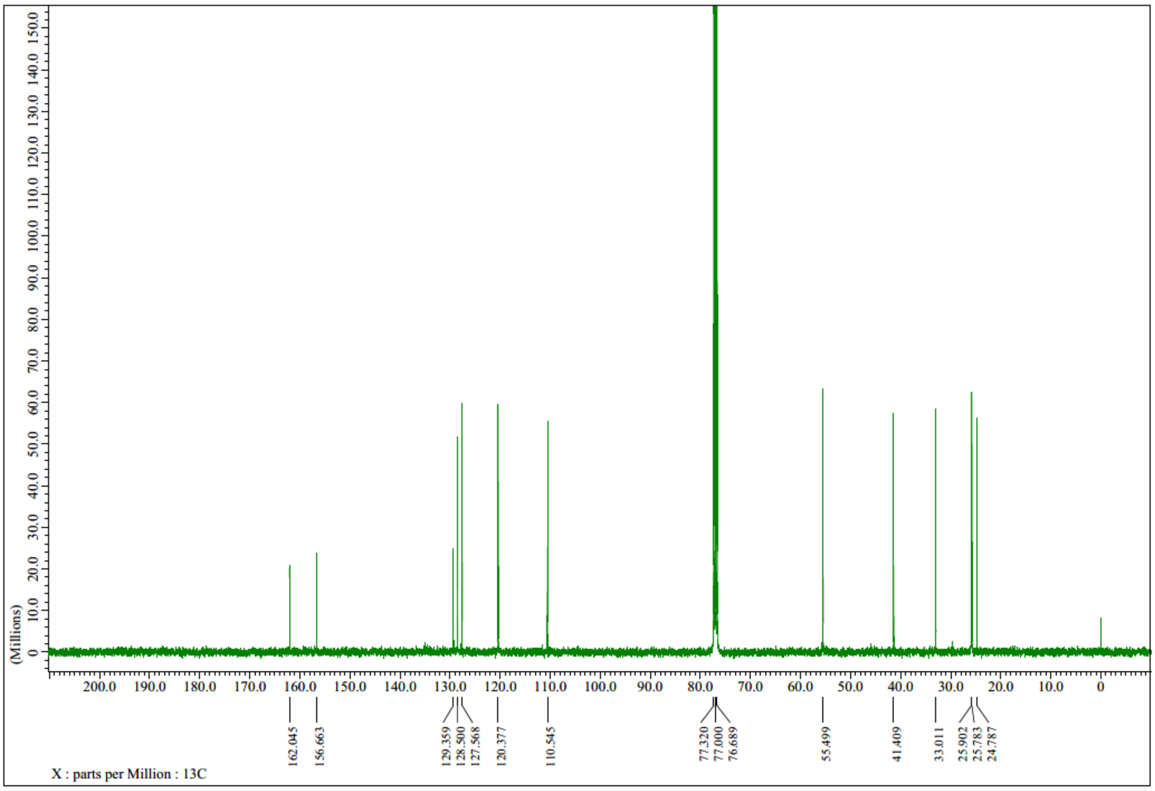
(Millions)
X : parts per Million : 13C
162.045
156.663
129.359
128.500
127.568
120.377
110.545
77.320
77.000
76.689
55.499
41.409
33.011
25.902
25.783
24.787

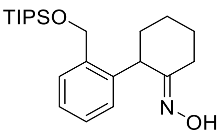
TIPSO
N
OH

3f

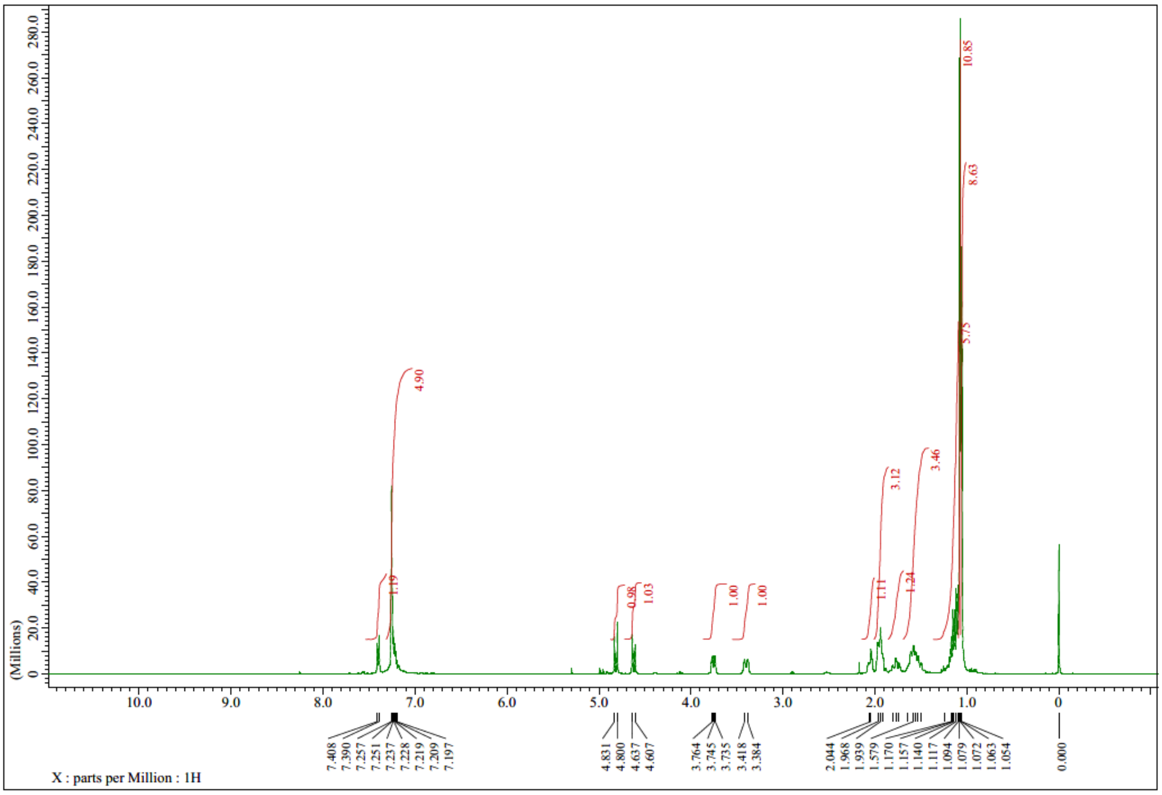
(Millions)
X : parts per Million : 1H
7.408
7.390
7.257
7.251
7.237
7.228
7.219
7.209
7.197
4.831
4.800
4.637
4.607
3.764
3.745
3.735
3.418
3.384
2.044
1.968
1.939
1.579
1.170
1.157
1.140
1.117
1.094
1.079
1.072
1.063
1.054
0.000
4.90
1.19
0.98
1.03
1.00
1.00
1.11
3.12
1.24
3.46
5.75
8.63
10.85

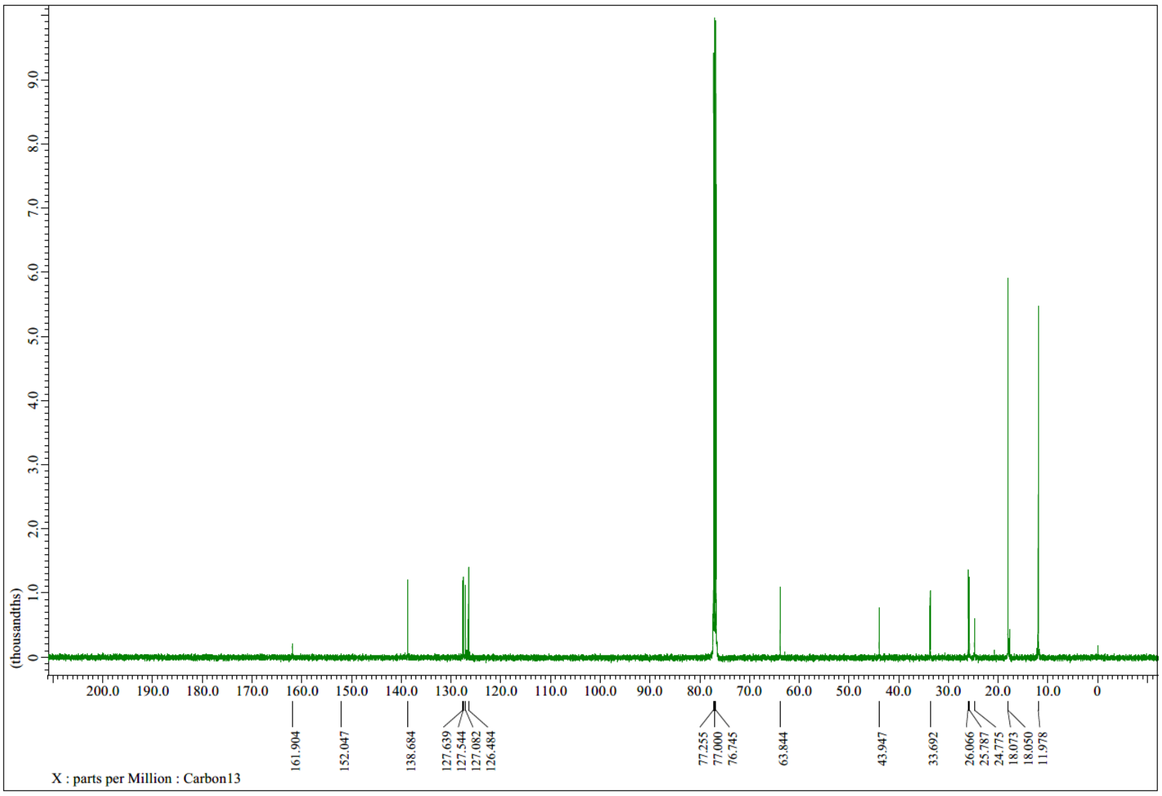
(thousandths)
X : parts per Million : Carbon13
161.904
152.047
138.684
127.639
127.544
127.082
126.484
77.255
77.000
76.745
63.844
43.947
33.692
26.066
25.787
24.775
18.073
18.050
11.978

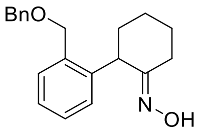

BnO
N
OH

3g

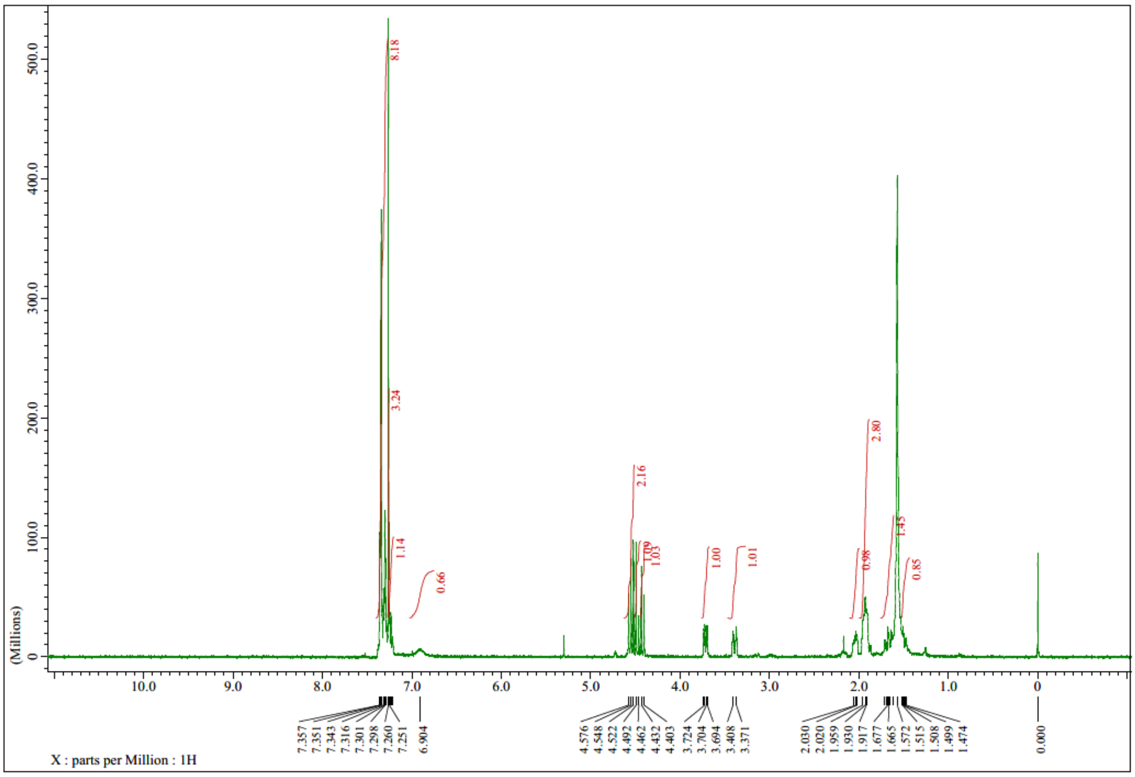

X : parts per Million : 1H
(Millions)

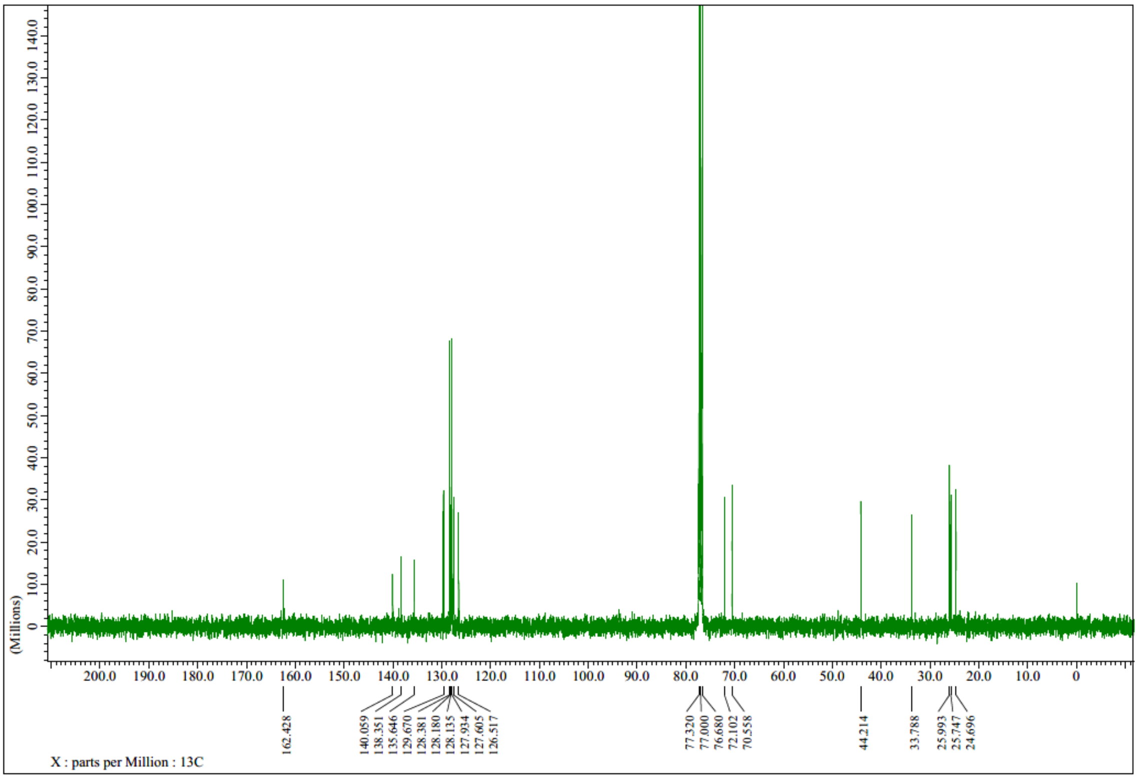

X : parts per Million : 13C
(Millions)

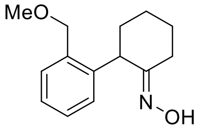

3h

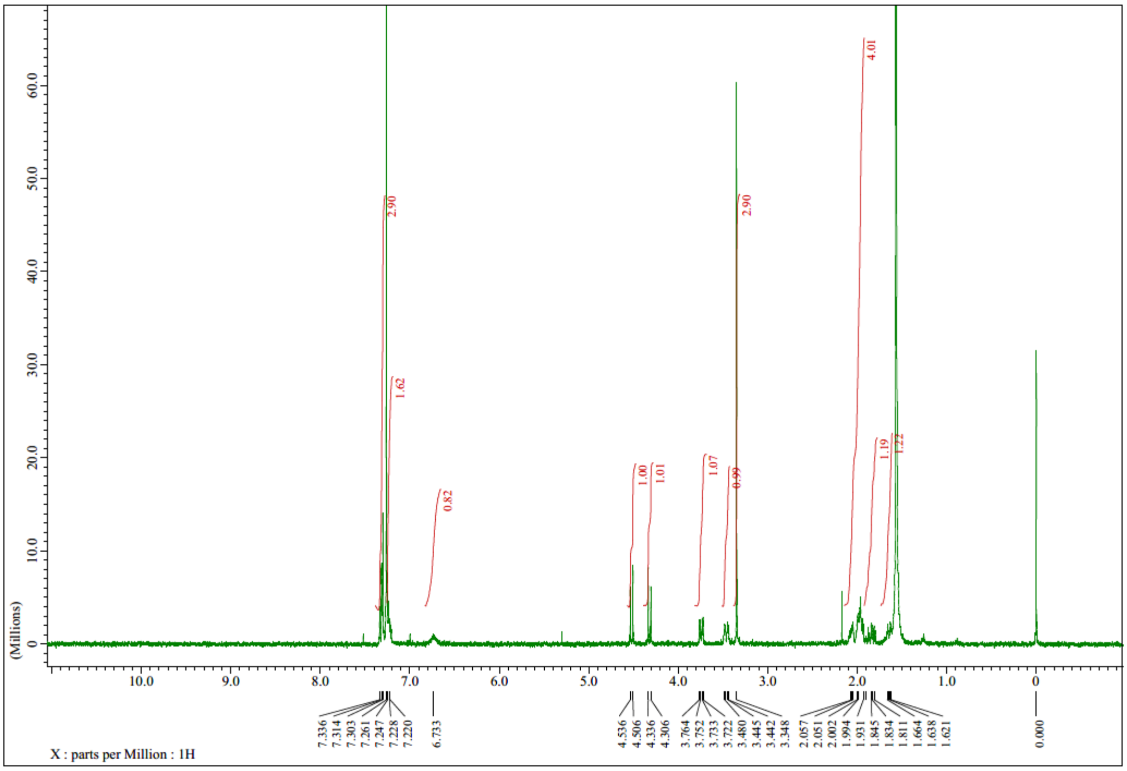

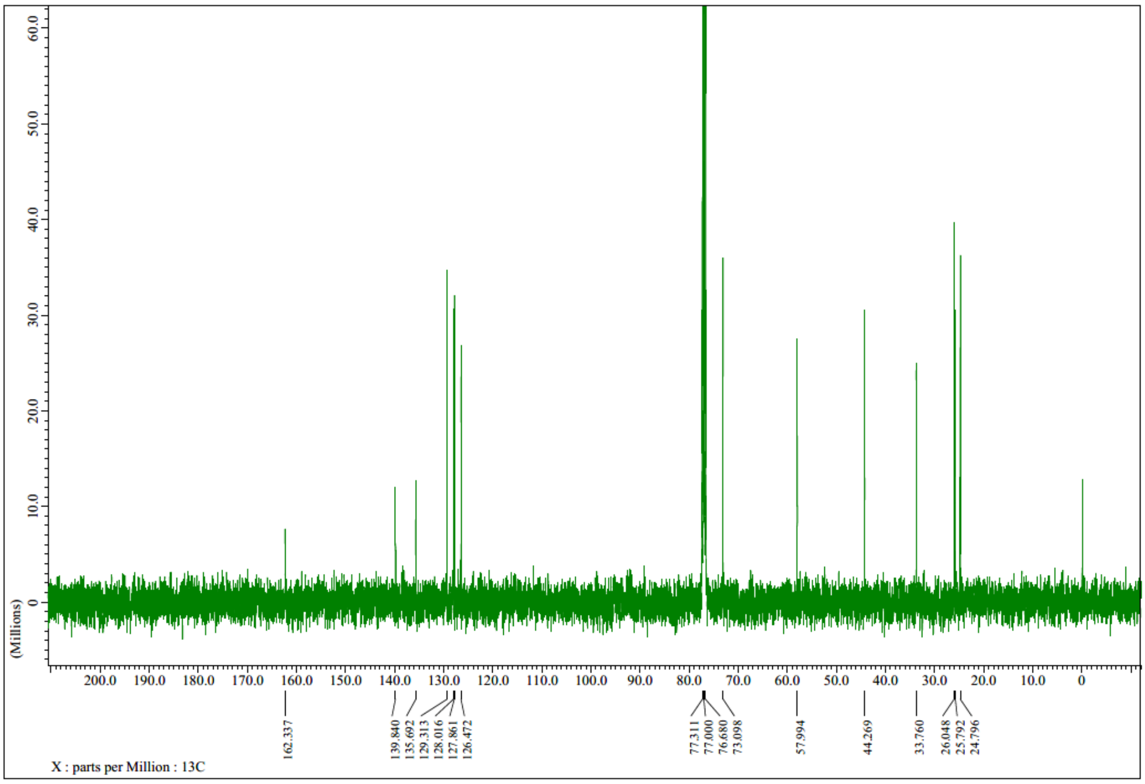

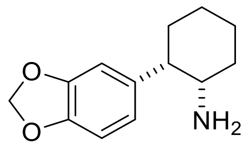
O
O
NH2

cis-**4a**

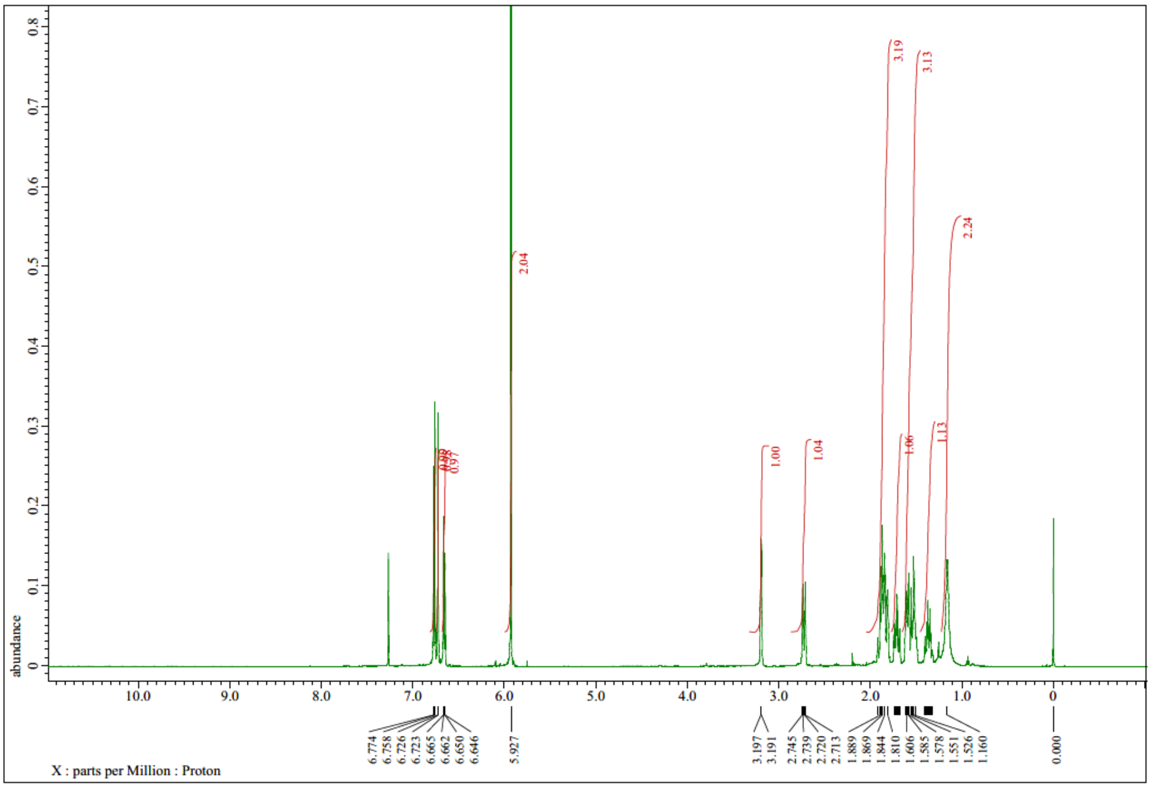
abundance
X : parts per Million : Proton
10.0 9.0 8.0 7.0 6.0 5.0 4.0 3.0 2.0 1.0 0
2.04
1.00
1.04
3.19
3.13
1.06
1.13
2.24
6.774 6.758 6.726 6.723 6.665 6.662 6.650 6.646
5.927
3.197 3.191
2.745 2.739 2.720 2.713
1.889 1.869 1.844 1.810 1.606 1.585 1.578 1.551 1.526 1.160
0.000

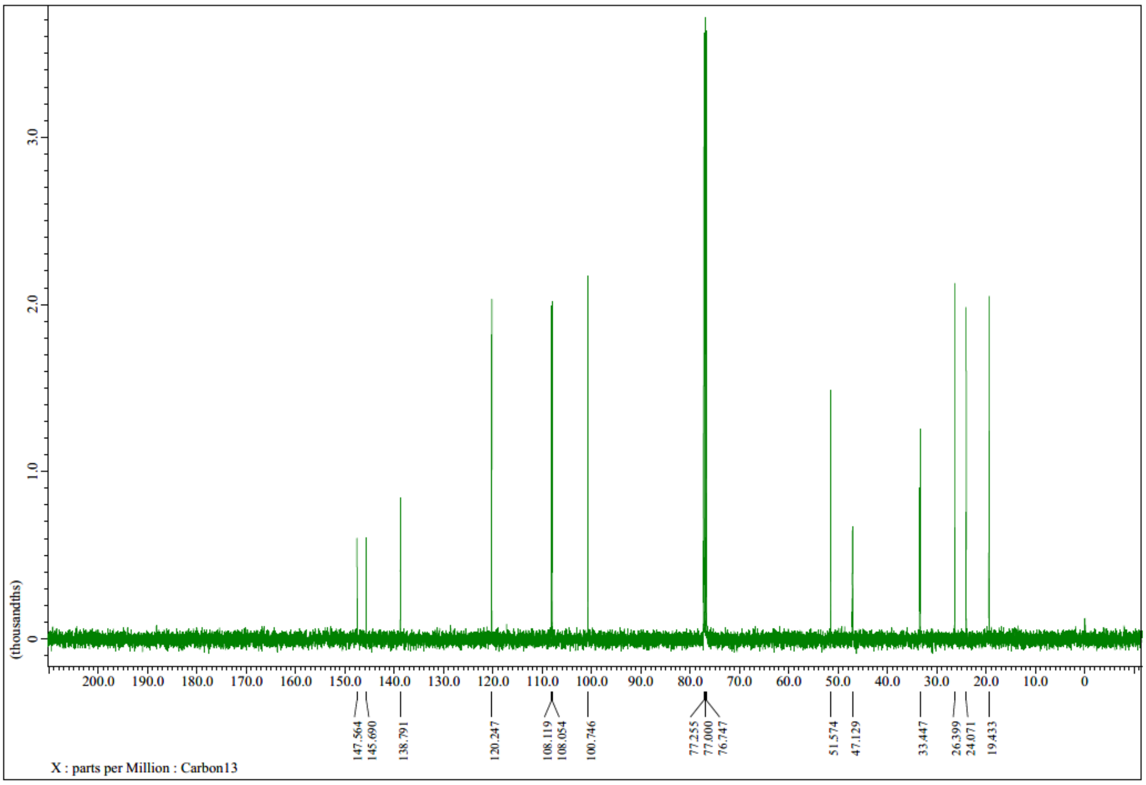
(thousandths)
X : parts per Million : Carbon13
200.0 190.0 180.0 170.0 160.0 150.0 140.0 130.0 120.0 110.0 100.0 90.0 80.0 70.0 60.0 50.0 40.0 30.0 20.0 10.0 0
147.564 145.690
138.791
120.247
108.119 108.054
100.746
77.255 77.000 76.747
51.574
47.129
33.447
26.399 24.071
19.433

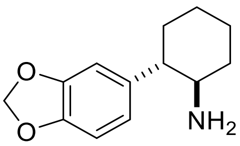
O
O
NH2

trans-**4a**

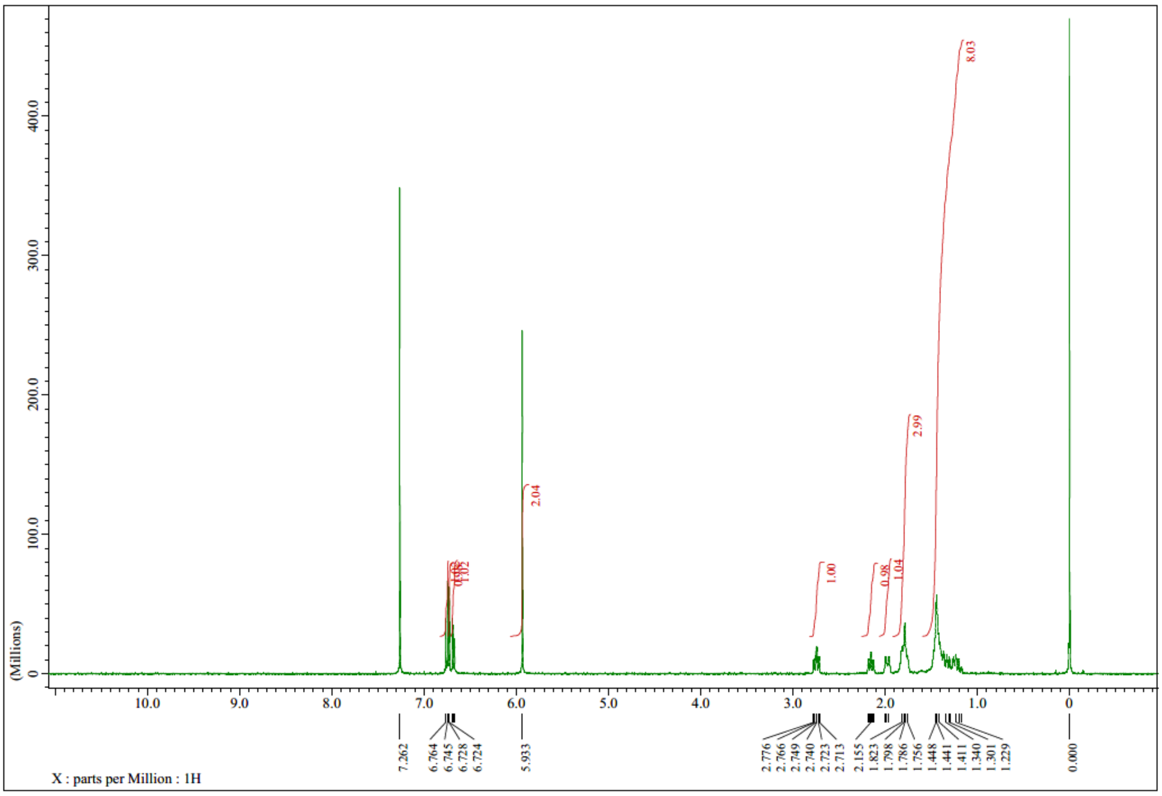
(Millions)
X : parts per Million : 1H
8.03
2.99
2.04
1.00
0.98
1.04
7.262
6.764
6.745
6.728
6.724
5.933
2.776
2.766
2.749
2.740
2.723
2.713
2.155
1.823
1.798
1.786
1.756
1.448
1.441
1.411
1.340
1.301
1.229
0.000

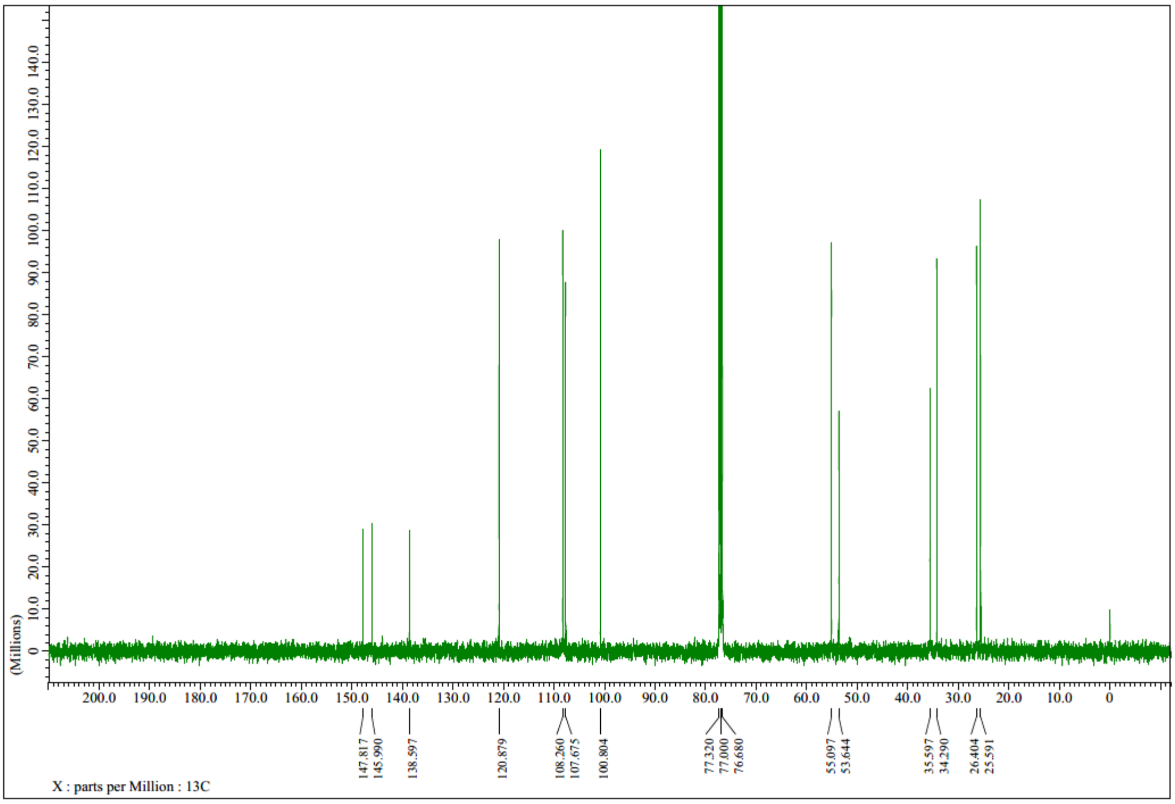
(Millions)
X : parts per Million : 13C
147.817
145.990
138.597
120.879
108.260
107.675
100.804
77.320
77.000
76.680
55.097
53.644
35.597
34.290
26.404
25.591

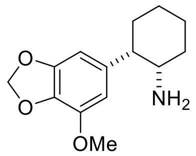
O
O
OMe
NH2

cis-**4b**

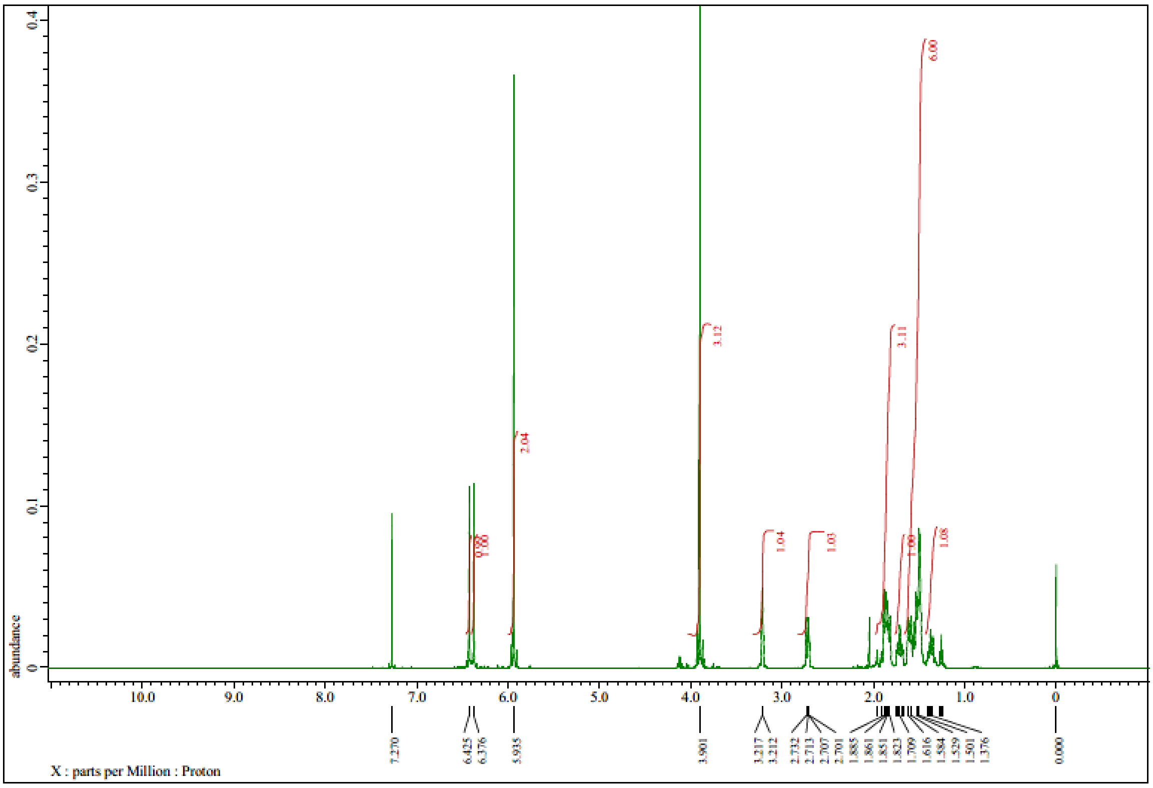
abundance
X : parts per Million : Proton
7.270
6.425
6.376
5.935
3.901
3.217
3.212
2.732
2.713
2.707
2.701
1.885
1.861
1.851
1.823
1.709
1.616
1.584
1.529
1.501
1.376
0.000
3.12
2.04
1.04
1.03
3.11
6.00
1.08

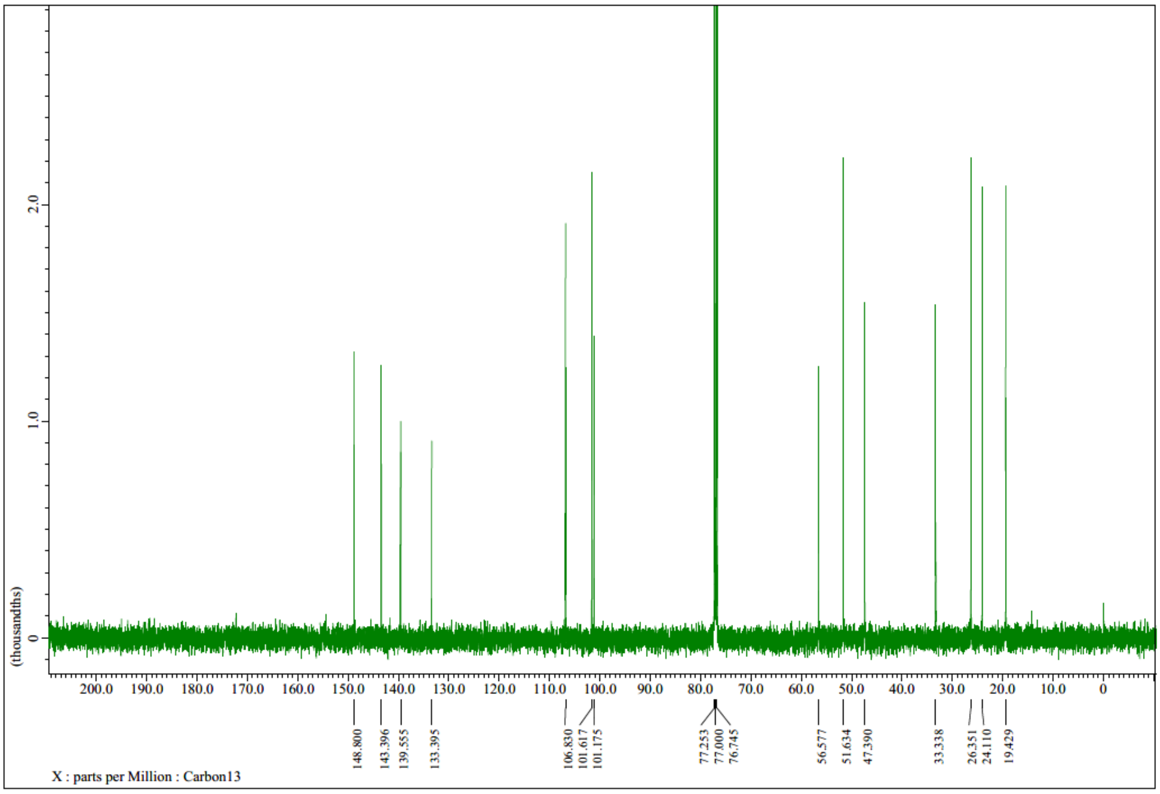
(thousandths)
X : parts per Million : Carbon13
148.800
143.396
139.555
133.395
106.830
101.617
101.175
77.253
77.000
76.745
56.577
51.634
47.390
33.338
26.351
24.110
19.429

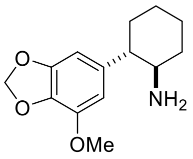
O
O
NH2
OMe

trans-**4b**

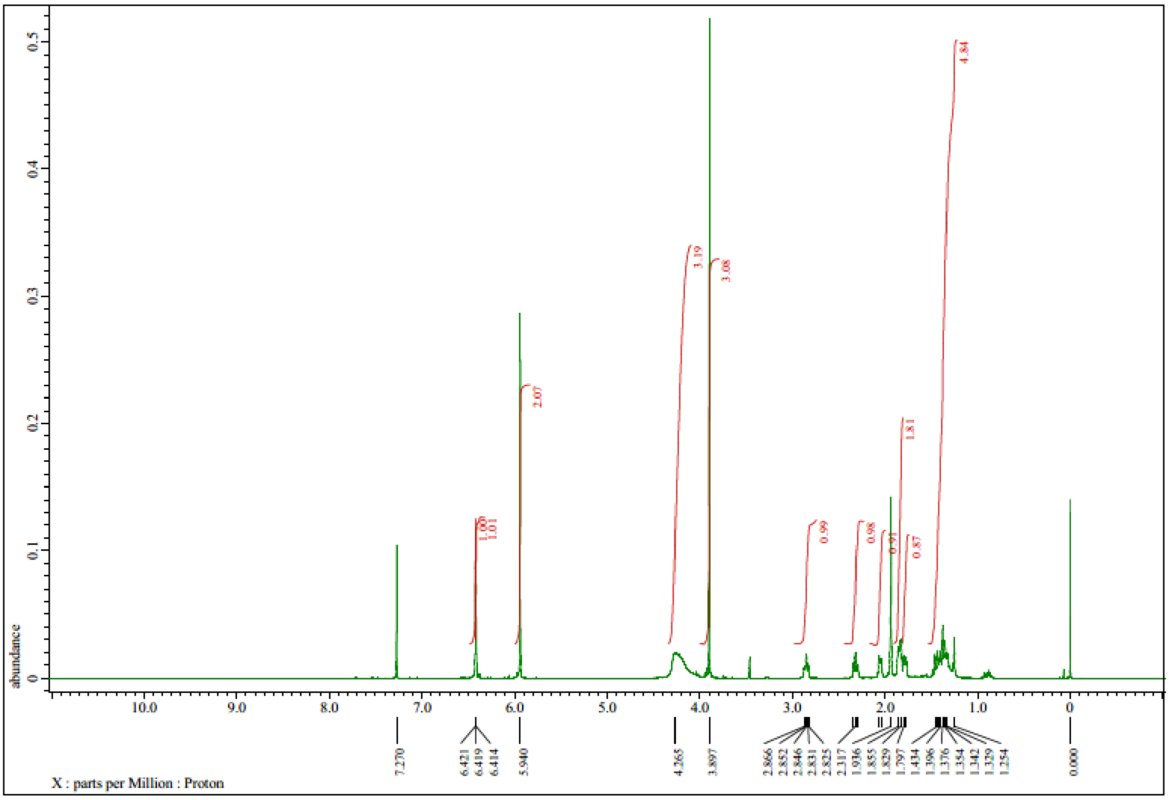
abundance
X : parts per Million : Proton
1.00
1.01
2.07
3.19
3.08
0.99
0.98
0.91
1.81
0.87
4.84
7.270
6.421
6.419
6.414
5.940
4.265
3.897
2.866
2.852
2.846
2.831
2.825
2.317
1.936
1.855
1.829
1.797
1.434
1.396
1.376
1.354
1.342
1.329
1.254
0.000

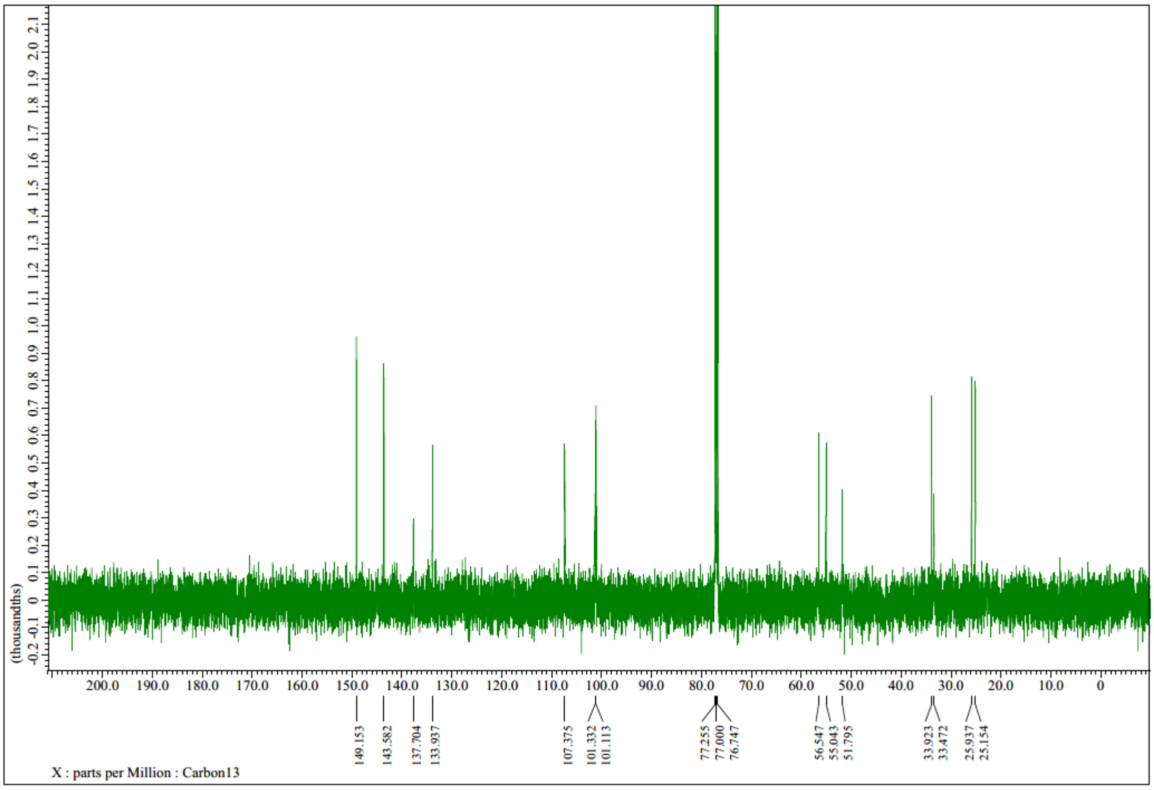
(thousandths)
X : parts per Million : Carbon13
149.153
143.582
137.704
133.937
107.375
101.332
101.113
77.255
77.000
76.747
56.547
55.043
51.795
33.923
33.472
25.937
25.154

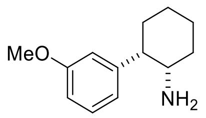
MeO
NH2

cis-**4c**

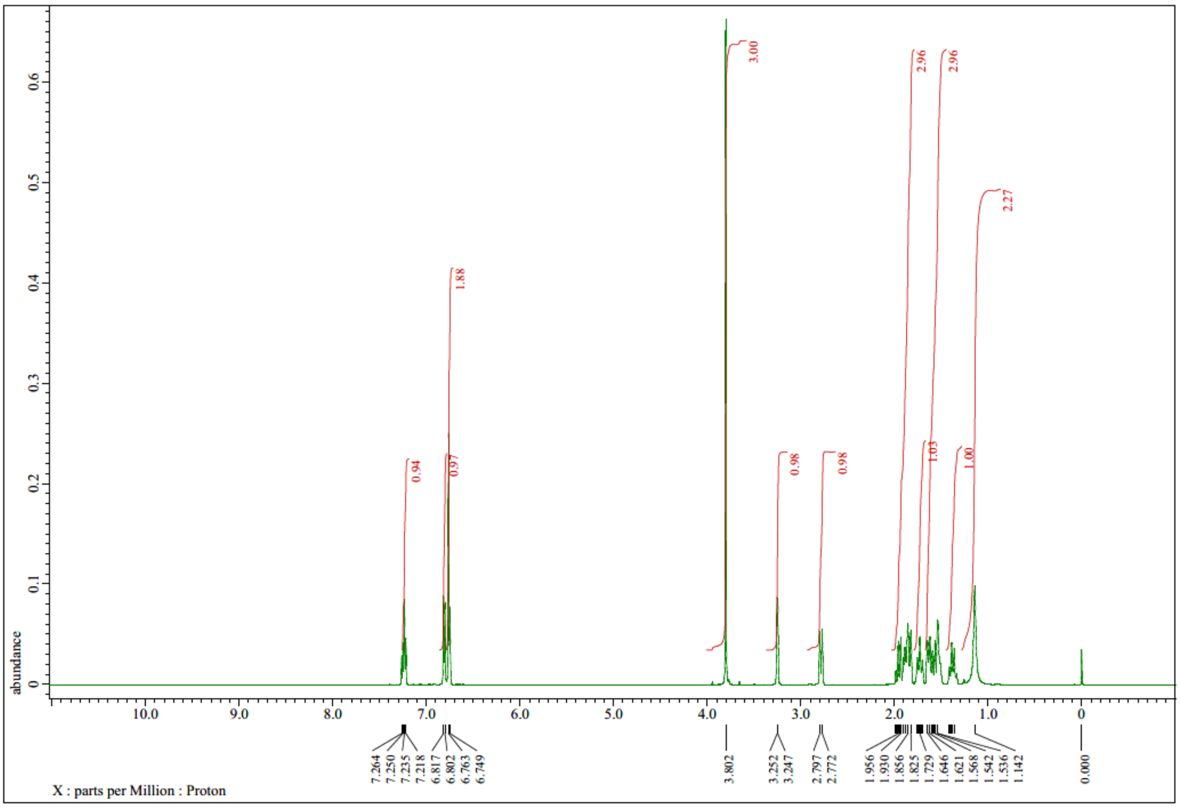
X : parts per Million : Proton
abundance
3.802
0.000

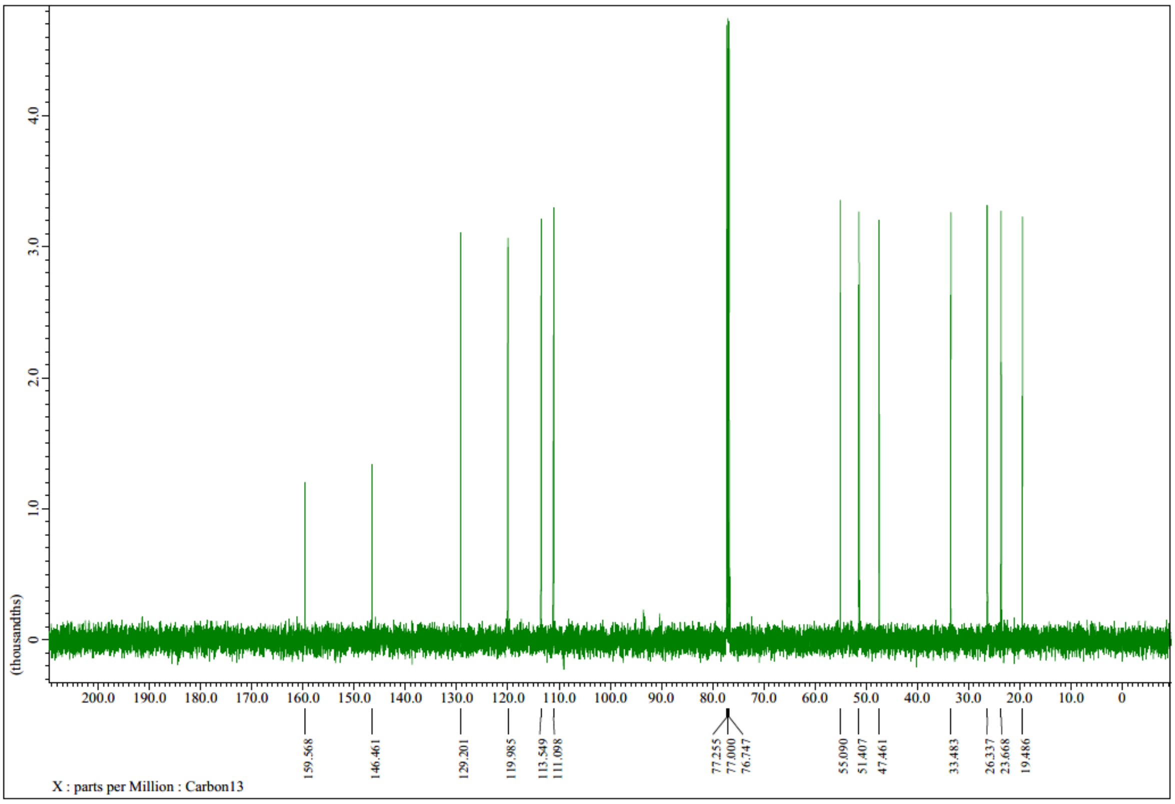
X : parts per Million : Carbon13
(thousandths)
159.568
146.461
129.201
119.985
113.549
111.098
77.255
77.000
76.747
55.090
51.407
47.461
33.483
26.337
23.668
19.486

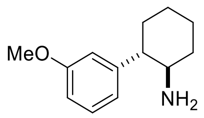
MeO
NH2

trans-**4c**

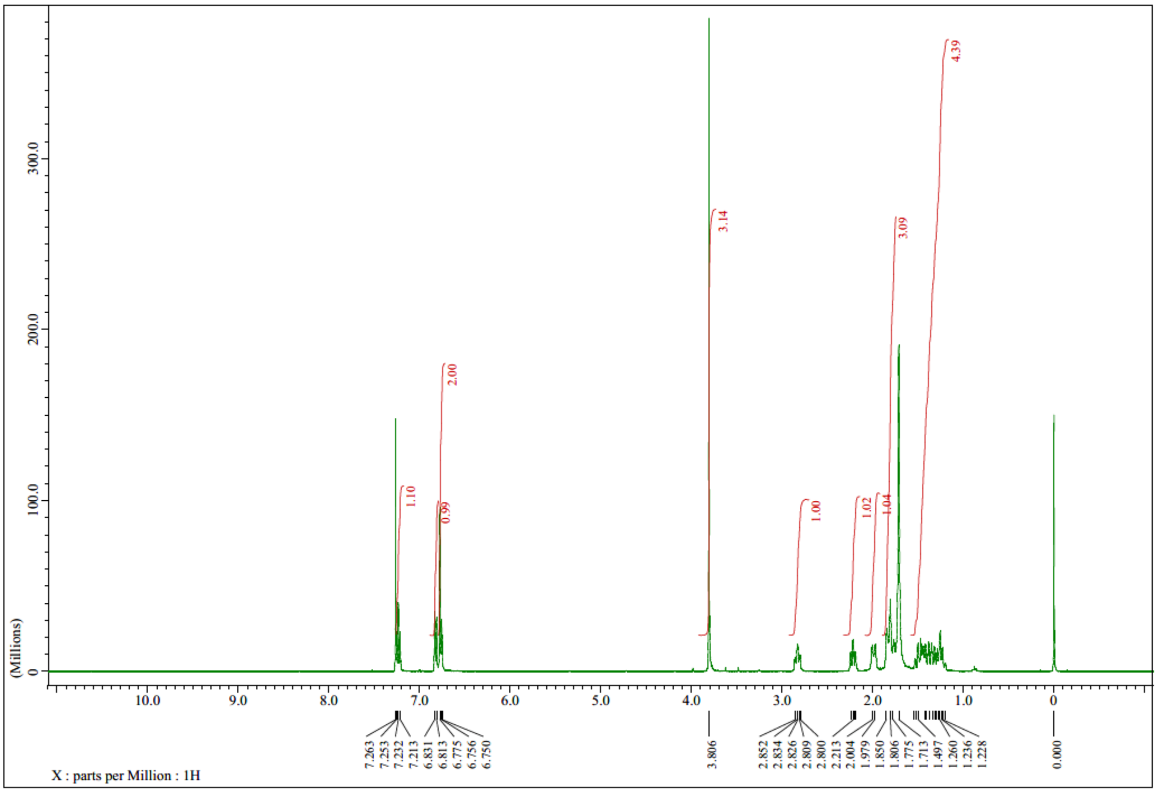
(Millions)
X : parts per Million : 1H
7.263
7.253
7.232
7.213
6.831
6.813
6.775
6.756
6.750
3.806
2.852
2.834
2.826
2.809
2.800
2.213
2.004
1.979
1.850
1.806
1.775
1.713
1.497
1.260
1.236
1.228
0.000
1.10
0.99
2.00
3.14
1.00
1.02
1.04
3.09
4.39

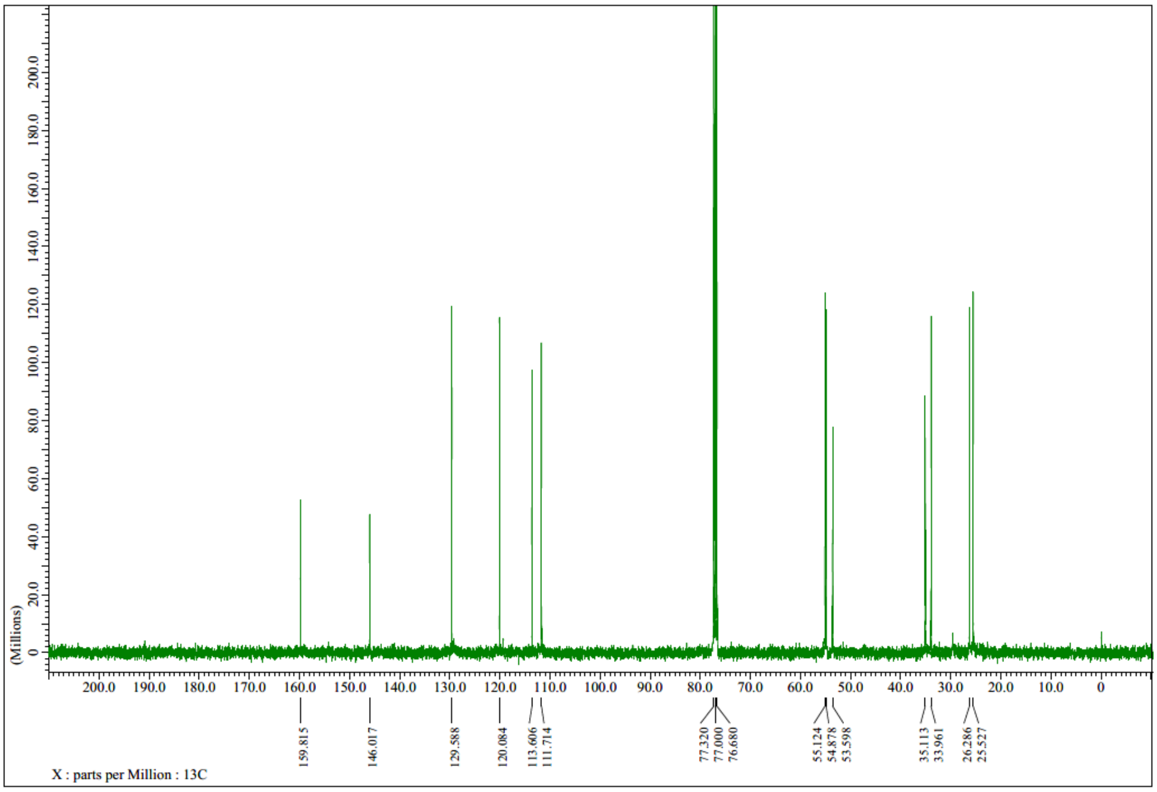
(Millions)
X : parts per Million : 13C
159.815
146.017
129.588
120.084
113.606
111.714
77.320
77.000
76.680
55.124
54.878
53.598
35.113
33.961
26.286
25.527

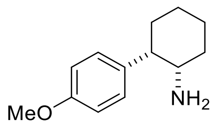
MeO
NH2

cis-**4d**

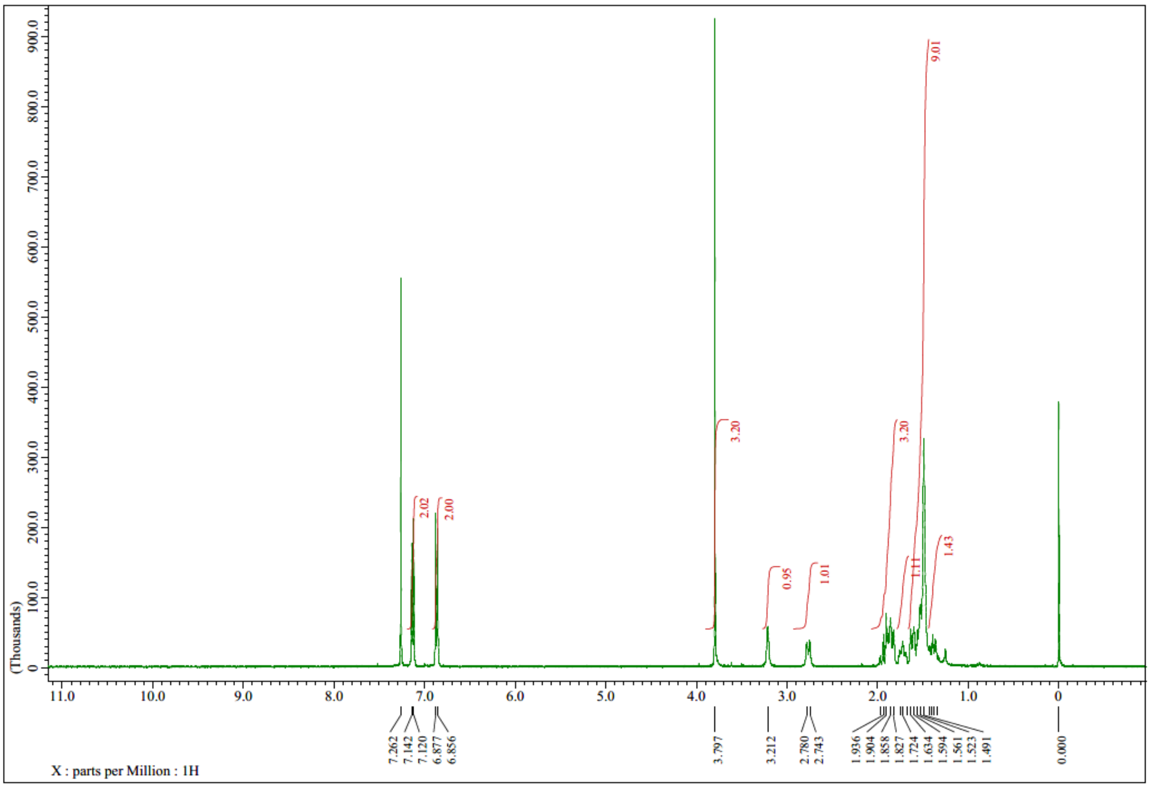
(Thousands)
3.20
2.02
2.00
0.95
1.01
3.20
1.11
9.01
1.43
7.262
7.142
7.120
6.877
6.856
3.797
3.212
2.780
2.743
1.936
1.904
1.858
1.827
1.724
1.634
1.594
1.561
1.523
1.491
0.000
X : parts per Million : 1H

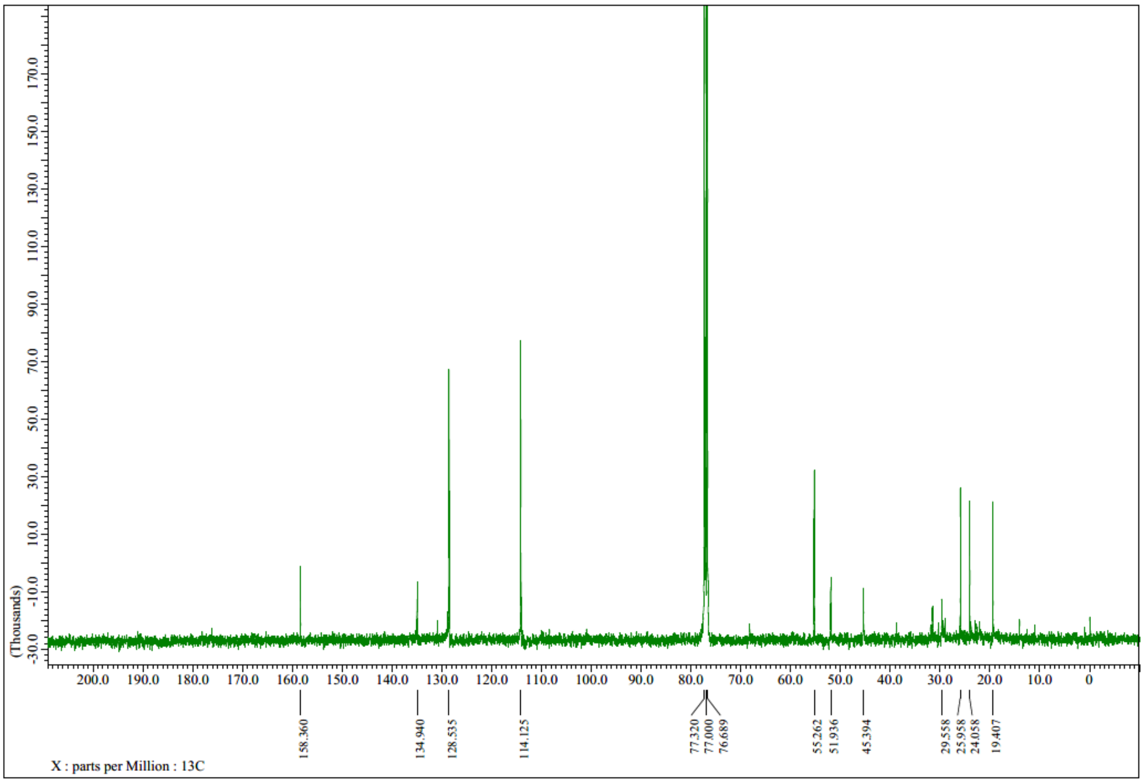
(Thousands)
158.360
134.940
128.535
114.125
77.320
77.000
76.689
55.262
51.936
45.394
29.558
25.958
24.058
19.407
X : parts per Million : 13C

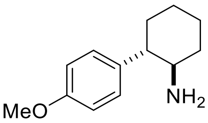
MeO
NH2
trans-**4d**

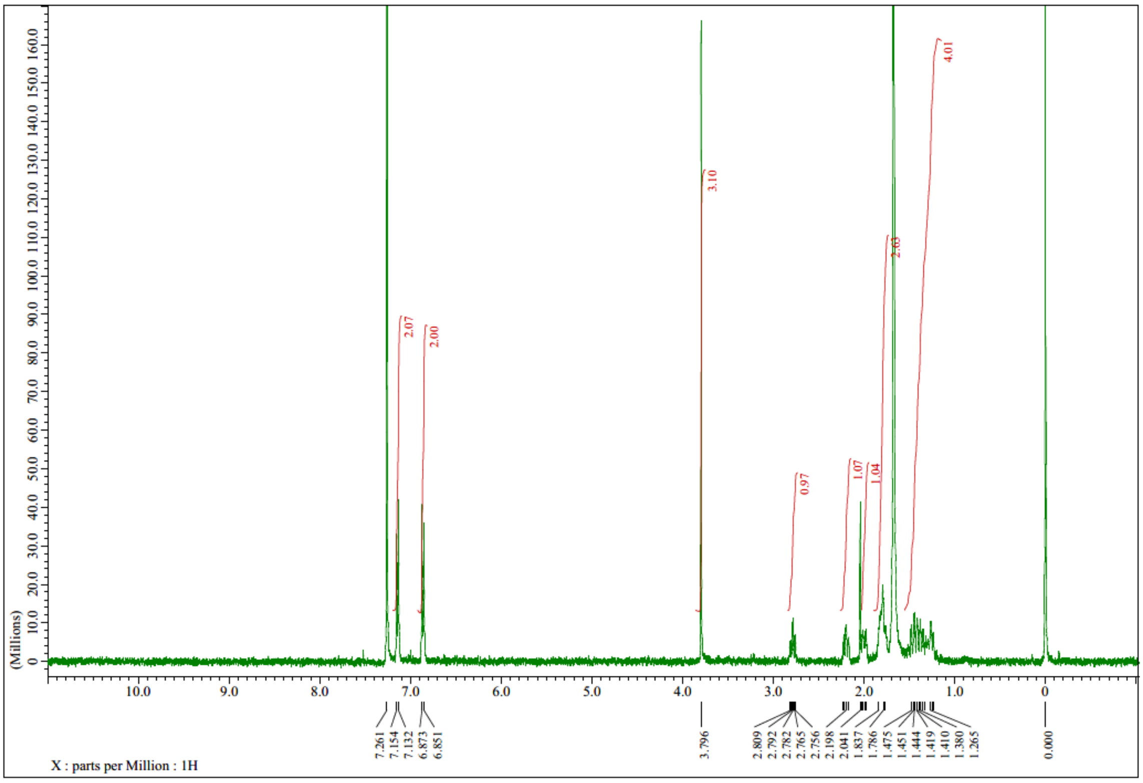
(Millions)
X : parts per Million : 1H
7.261
7.154
7.132
6.873
6.851
3.796
2.809
2.792
2.782
2.765
2.756
2.198
2.041
1.837
1.786
1.475
1.451
1.444
1.419
1.410
1.380
1.265
0.000
2.07
2.00
3.10
0.97
1.07
1.04
2.03
4.01

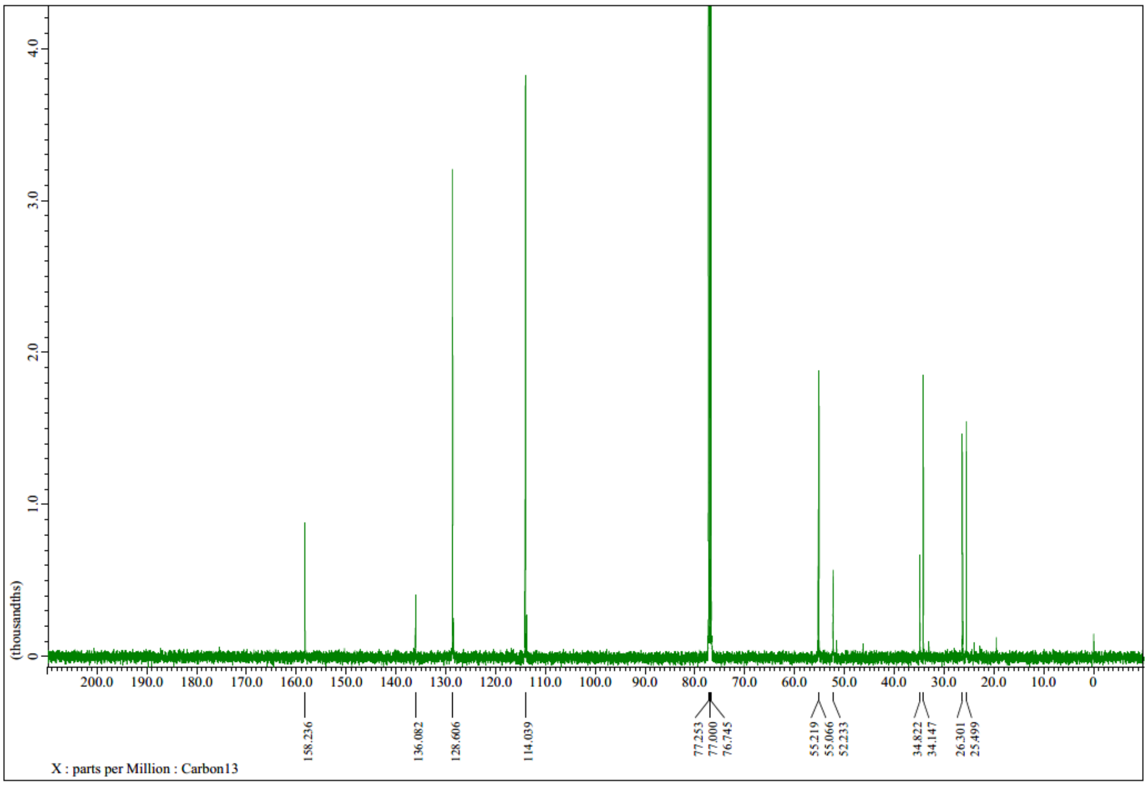
(thousandths)
X : parts per Million : Carbon13
158.236
136.082
128.606
114.039
77.253
77.000
76.745
55.219
55.066
52.233
34.822
34.147
26.301
25.499

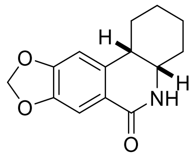
H
O
O
NH
H
O

cis-**5a**

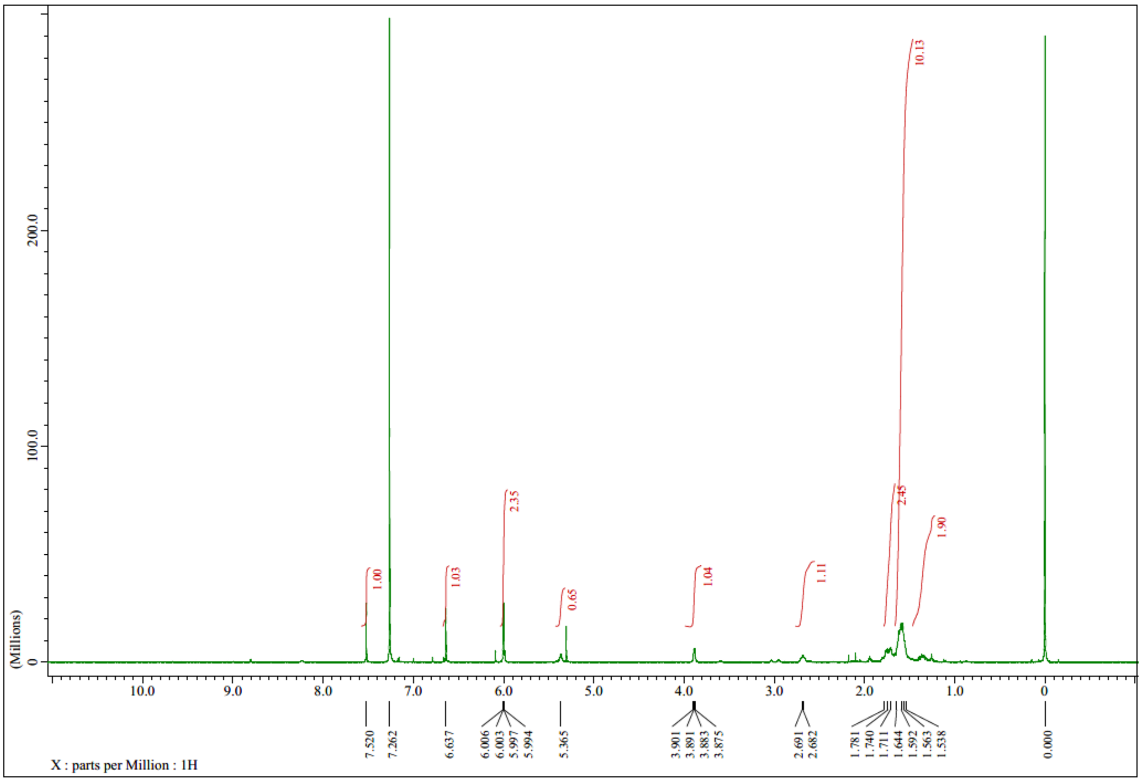
(Millions)
0
100.0
200.0
1.00
1.03
2.35
0.65
1.04
1.11
2.45
10.13
1.90
10.0
9.0
8.0
7.0
6.0
5.0
4.0
3.0
2.0
1.0
0
7.520
7.262
6.637
6.006
6.003
5.997
5.994
5.365
3.901
3.891
3.883
3.875
2.691
2.682
1.781
1.740
1.711
1.644
1.592
1.563
1.538
0.000
X : parts per Million : 1H

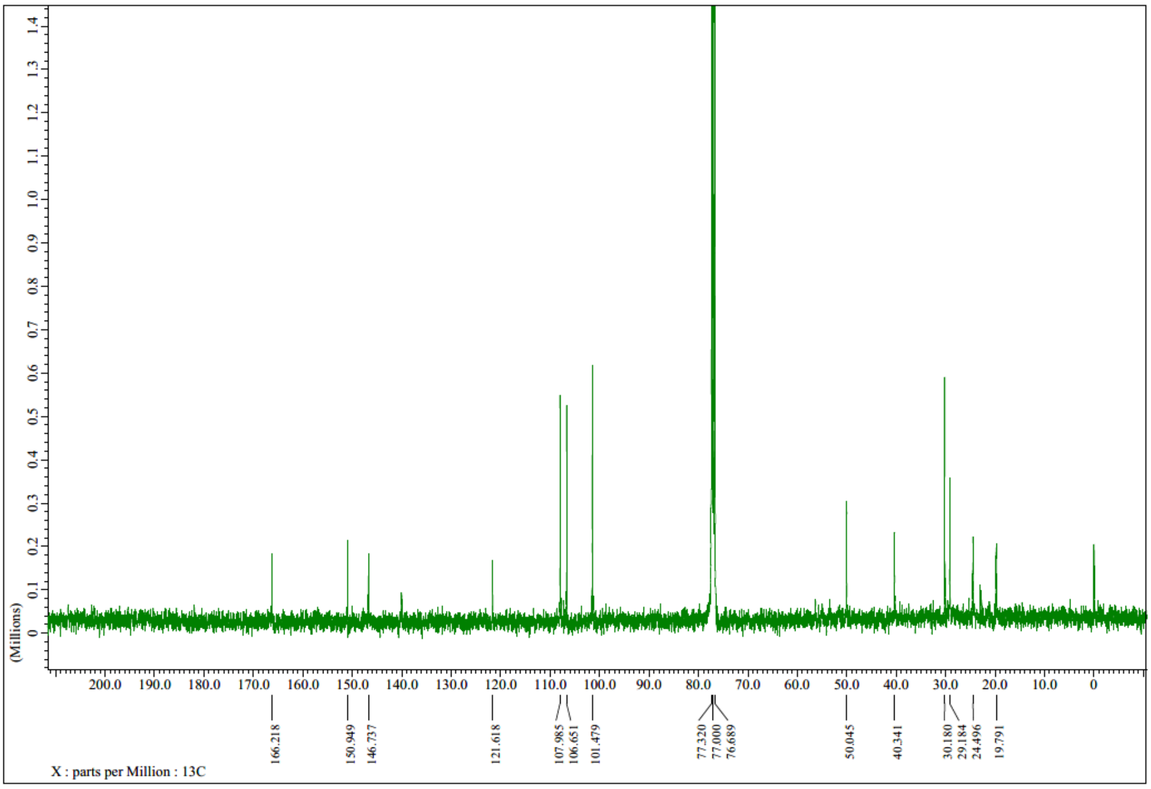
(Millions)
0
0.1
0.2
0.3
0.4
0.5
0.6
0.7
0.8
0.9
1.0
1.1
1.2
1.3
1.4
200.0
190.0
180.0
170.0
160.0
150.0
140.0
130.0
120.0
110.0
100.0
90.0
80.0
70.0
60.0
50.0
40.0
30.0
20.0
10.0
0
166.218
150.949
146.737
121.618
107.985
106.651
101.479
77.320
77.000
76.689
50.045
40.341
30.180
29.184
24.496
19.791
X : parts per Million : 13C

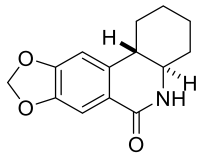

trans-**5a**

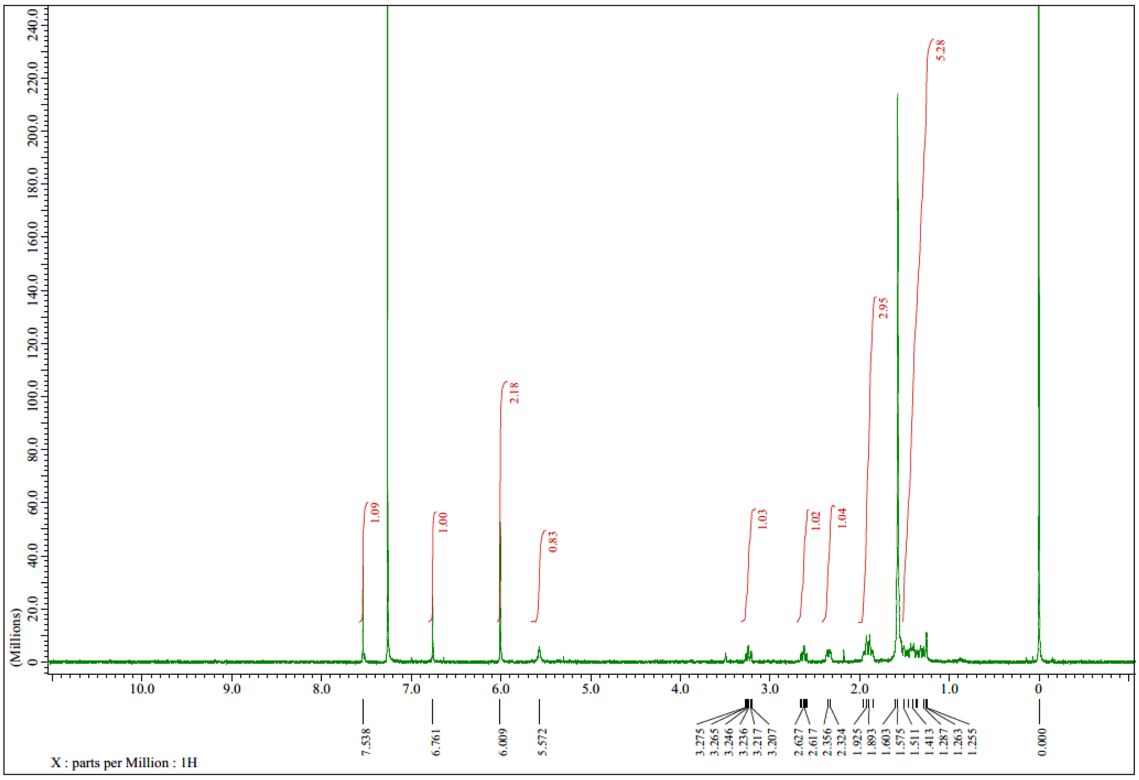

X : parts per Million : 1H
(Millions)

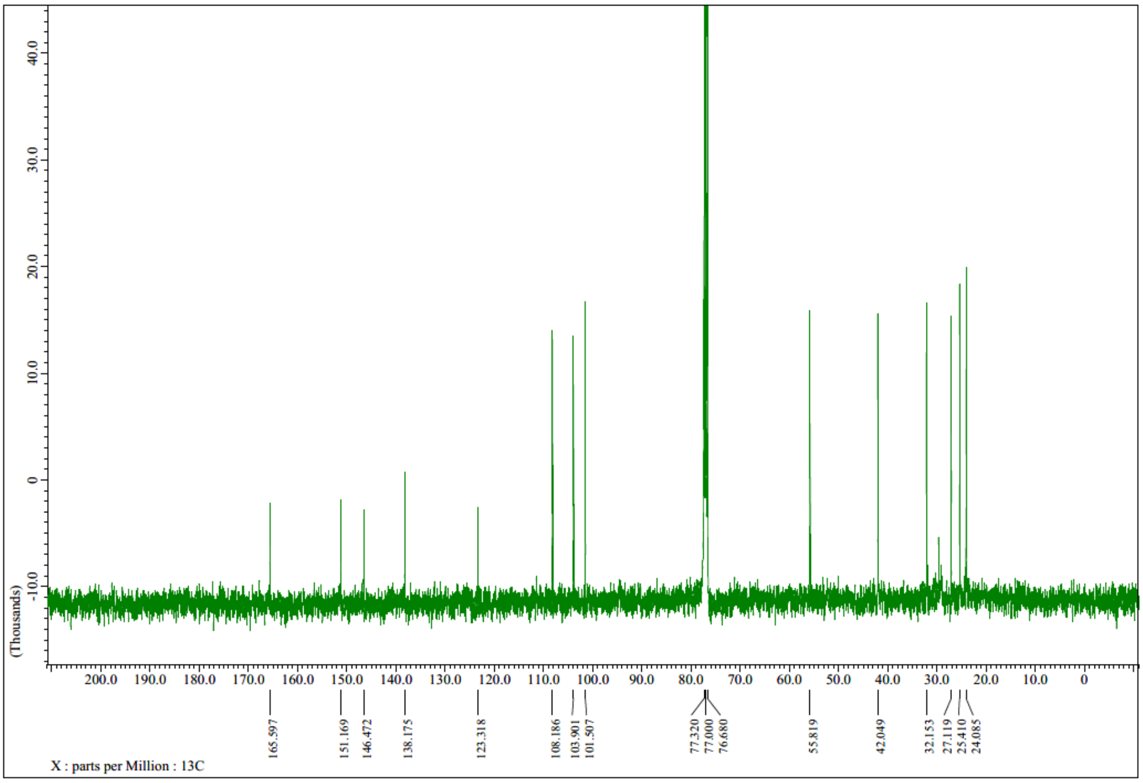

X : parts per Million : 13C
(Thousands)

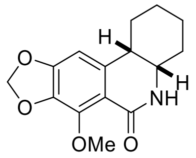
H
O
O
NH
H
OMe
O

cis-**5b**

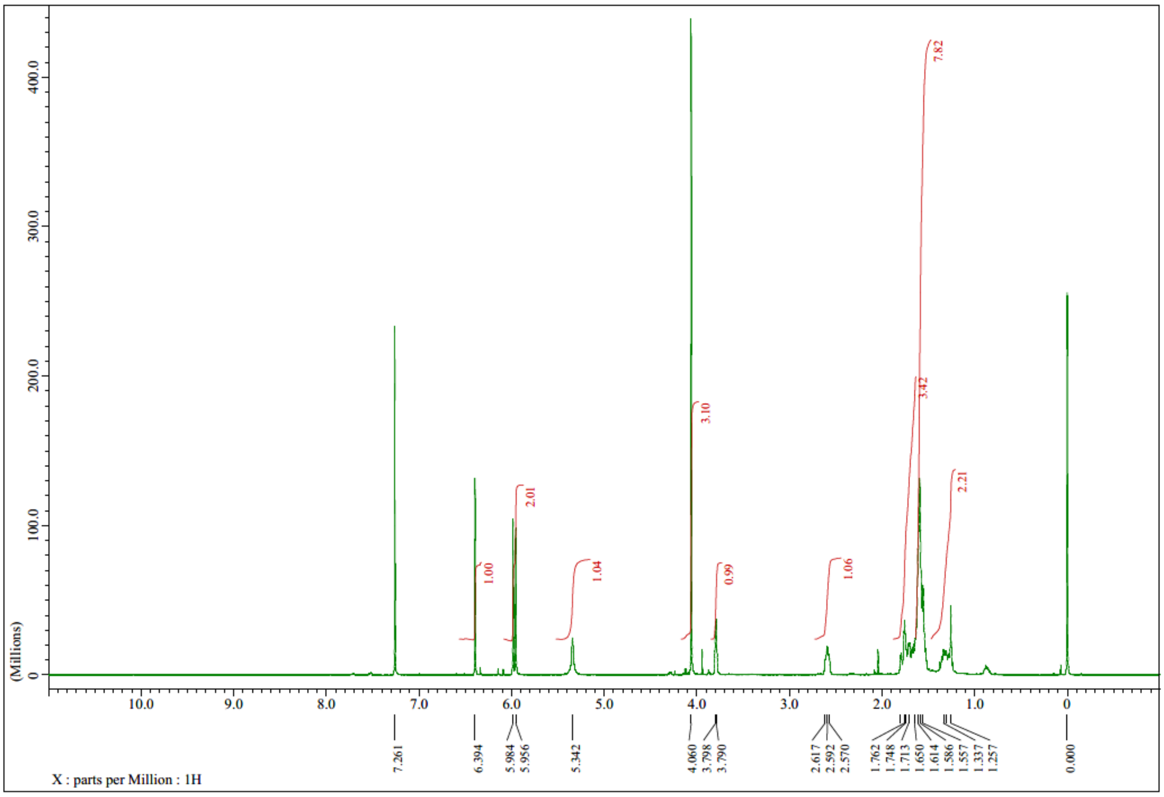
(Millions)
X : parts per Million : 1H
7.261
6.394
5.984
5.956
5.342
4.060
3.798
3.790
2.617
2.592
2.570
1.762
1.748
1.713
1.650
1.614
1.586
1.557
1.337
1.257
0.000
1.00
2.01
1.04
3.10
0.99
1.06
3.42
7.82
2.21

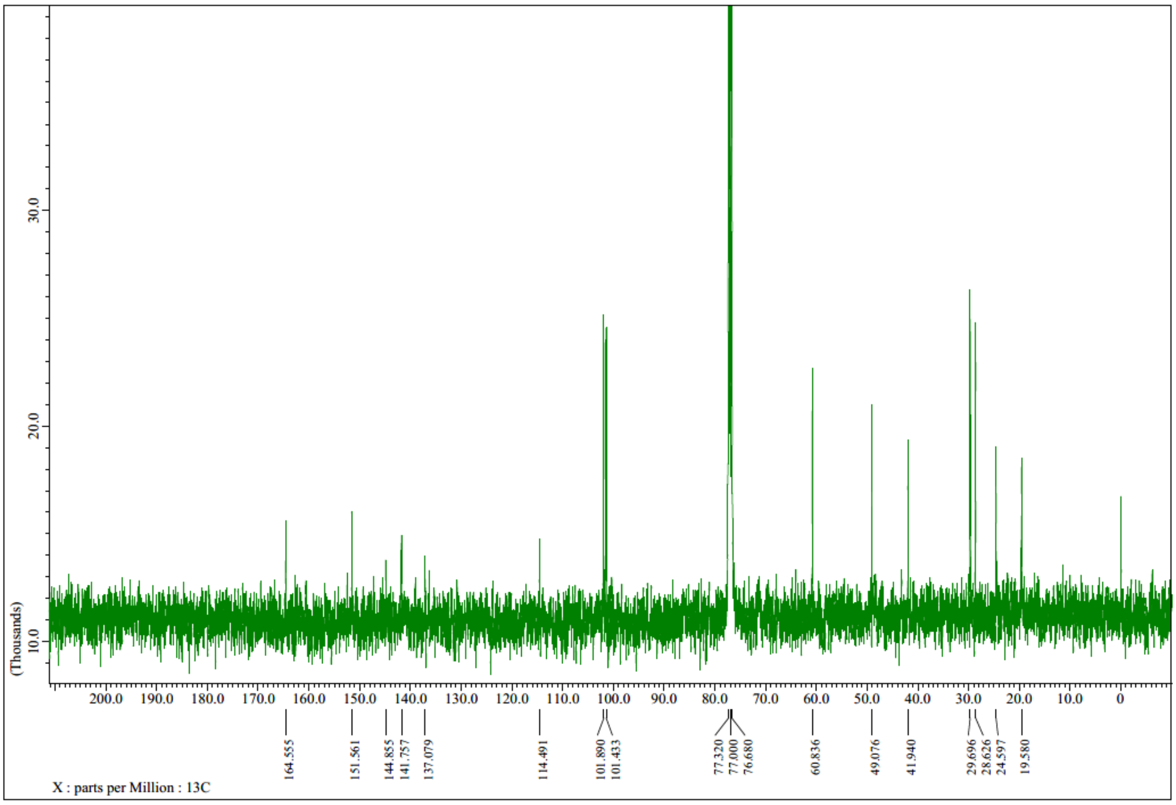
(Thousands)
X : parts per Million : 13C
164.555
151.561
144.855
141.757
137.079
114.491
101.890
101.433
77.320
77.000
76.680
60.836
49.076
41.940
29.696
28.626
24.597
19.580

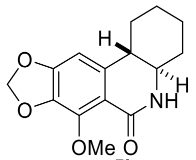
H
O
O
NH
H
OMe O

trans-**5b**

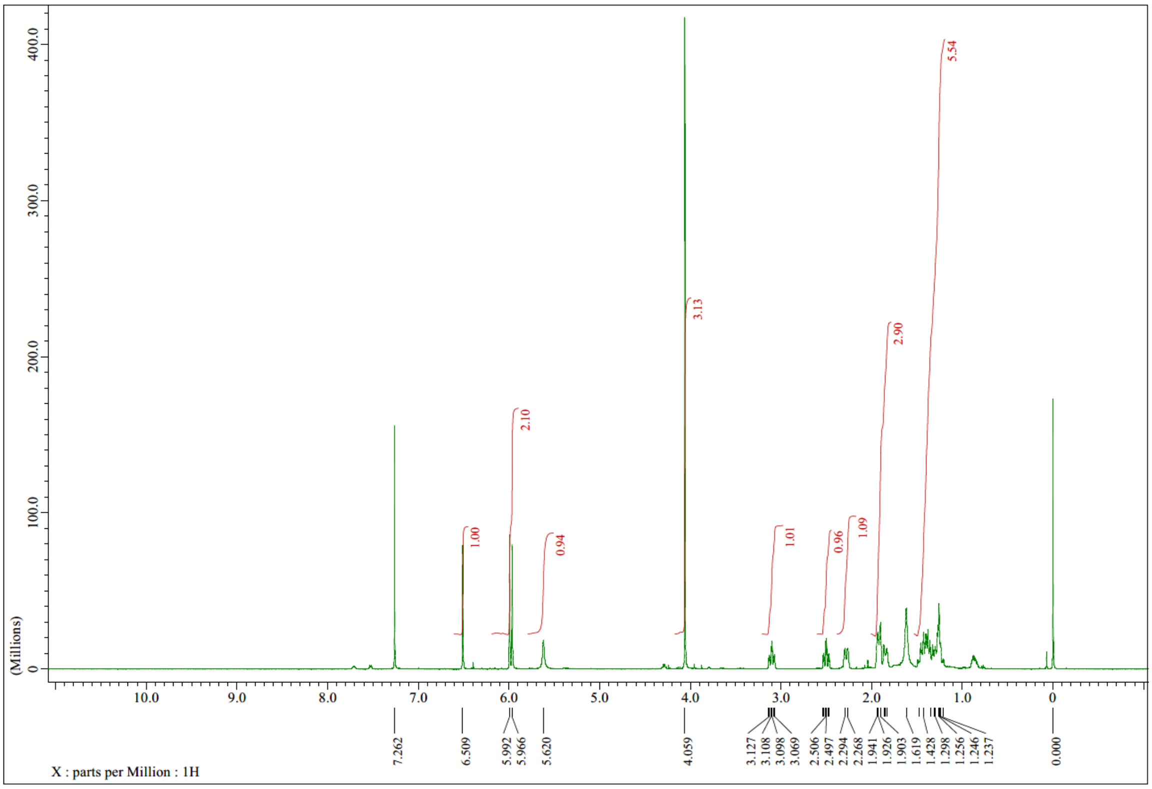
(Millions)
X : parts per Million : 1H
7.262
6.509
5.992
5.966
5.620
4.059
3.127
3.108
3.098
3.069
2.506
2.497
2.294
2.268
1.941
1.926
1.903
1.619
1.428
1.298
1.256
1.246
1.237
0.000
1.00
2.10
0.94
3.13
1.01
0.96
1.09
2.90
5.54

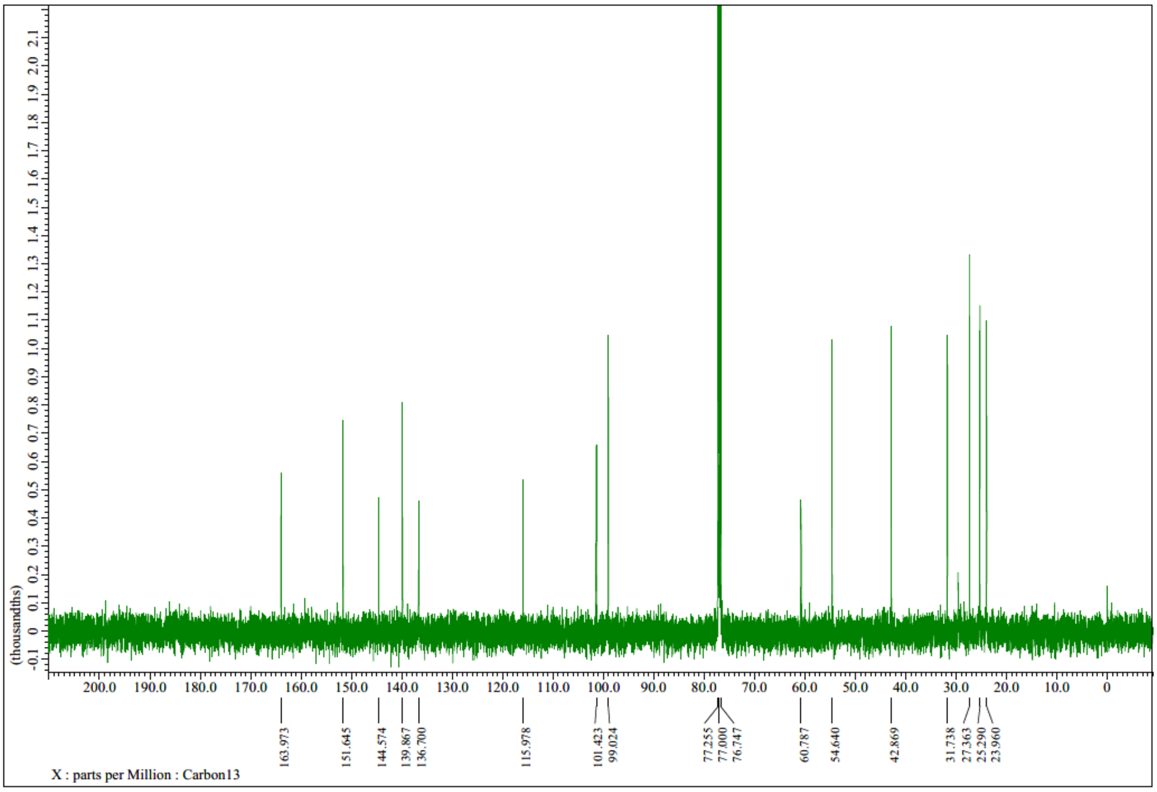
(thousandths)
X : parts per Million : Carbon13
163.973
151.645
144.574
139.867
136.700
115.978
101.423
99.024
77.255
77.000
76.747
60.787
54.640
42.869
31.738
27.363
25.290
23.960

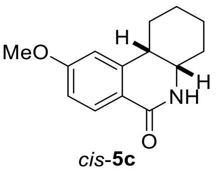

cis-**5c**

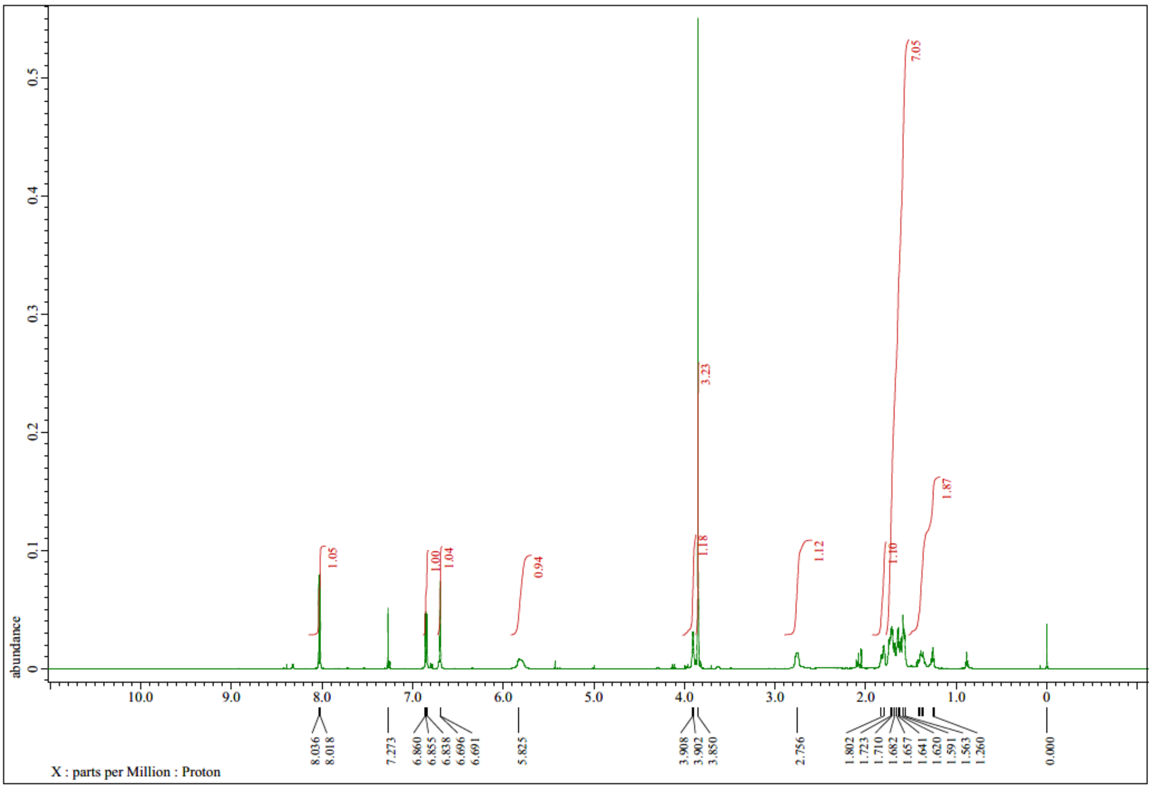

X : parts per Million : Proton

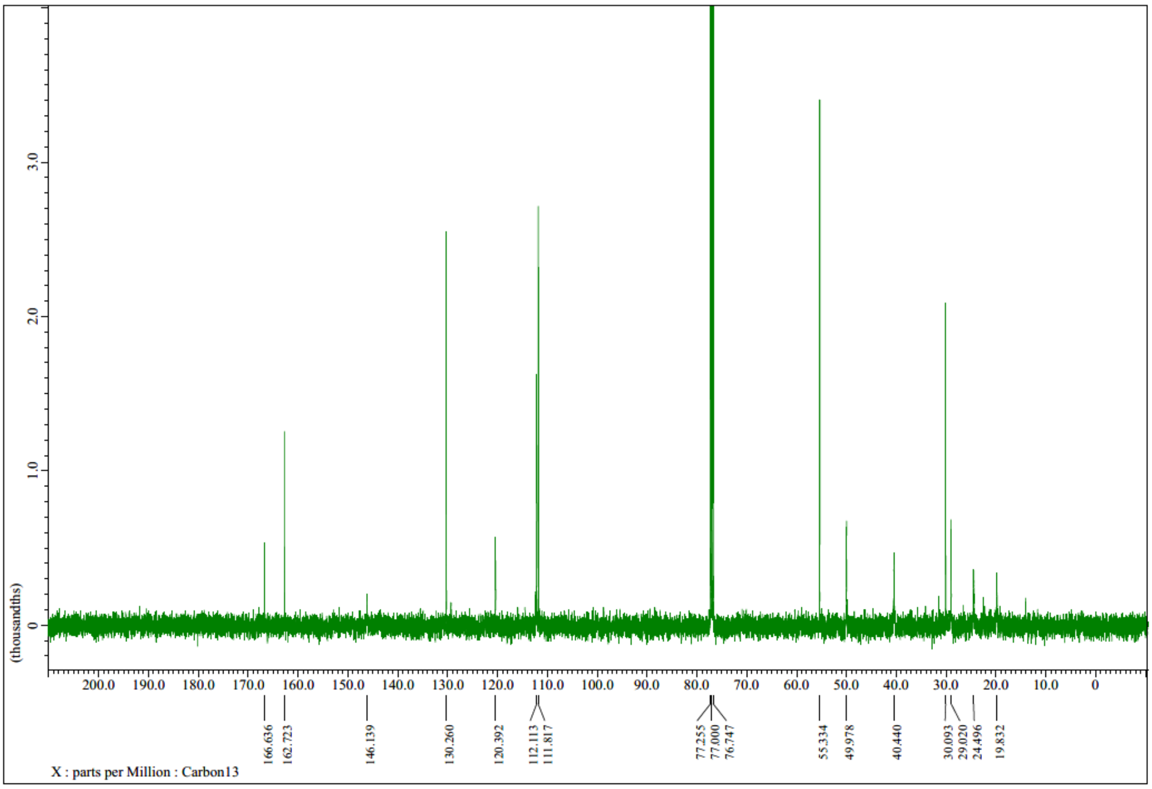

X : parts per Million : Carbon13

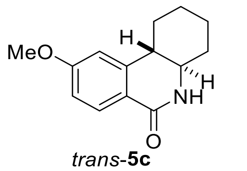
MeO
H
NH
H
O

trans-**5c**

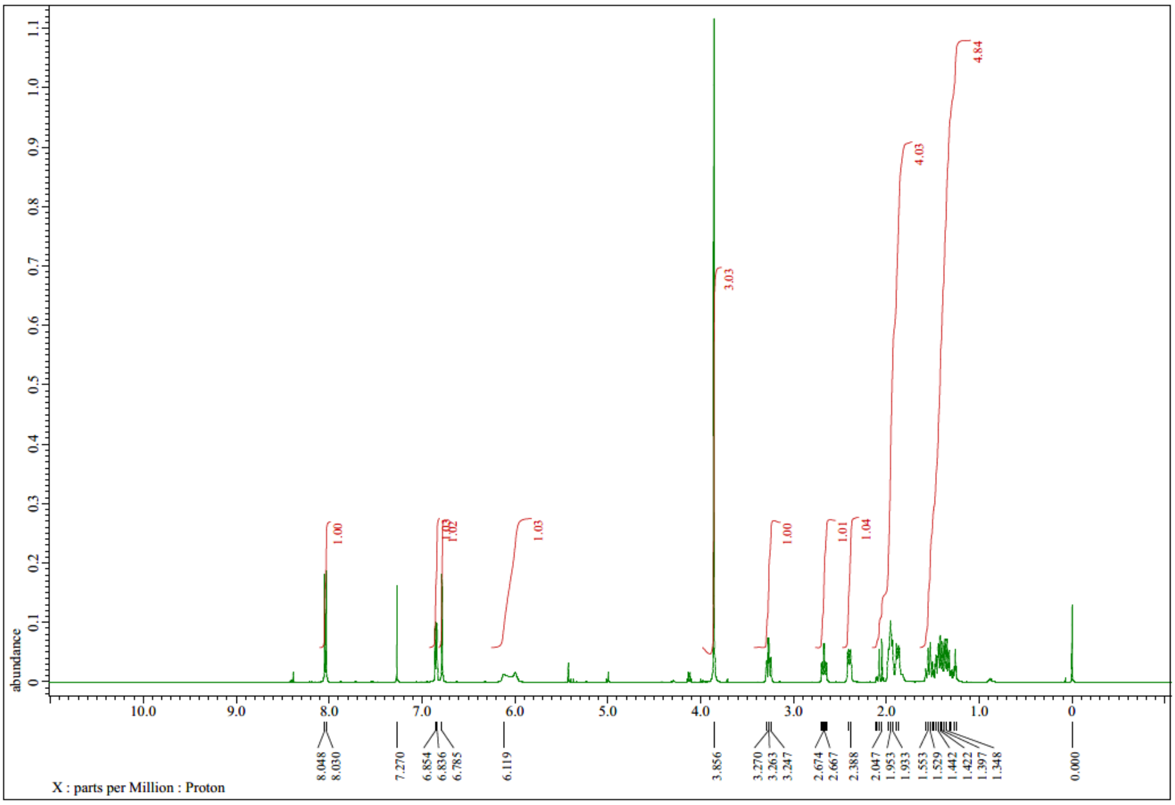
abundance
X : parts per Million : Proton
8.048
8.030
7.270
6.854
6.836
6.785
6.119
3.856
3.270
3.263
3.247
2.674
2.667
2.388
2.047
1.953
1.933
1.553
1.529
1.442
1.422
1.397
1.348
0.000
1.00
1.03
1.02
1.03
3.03
1.00
1.01
1.04
4.03
4.84

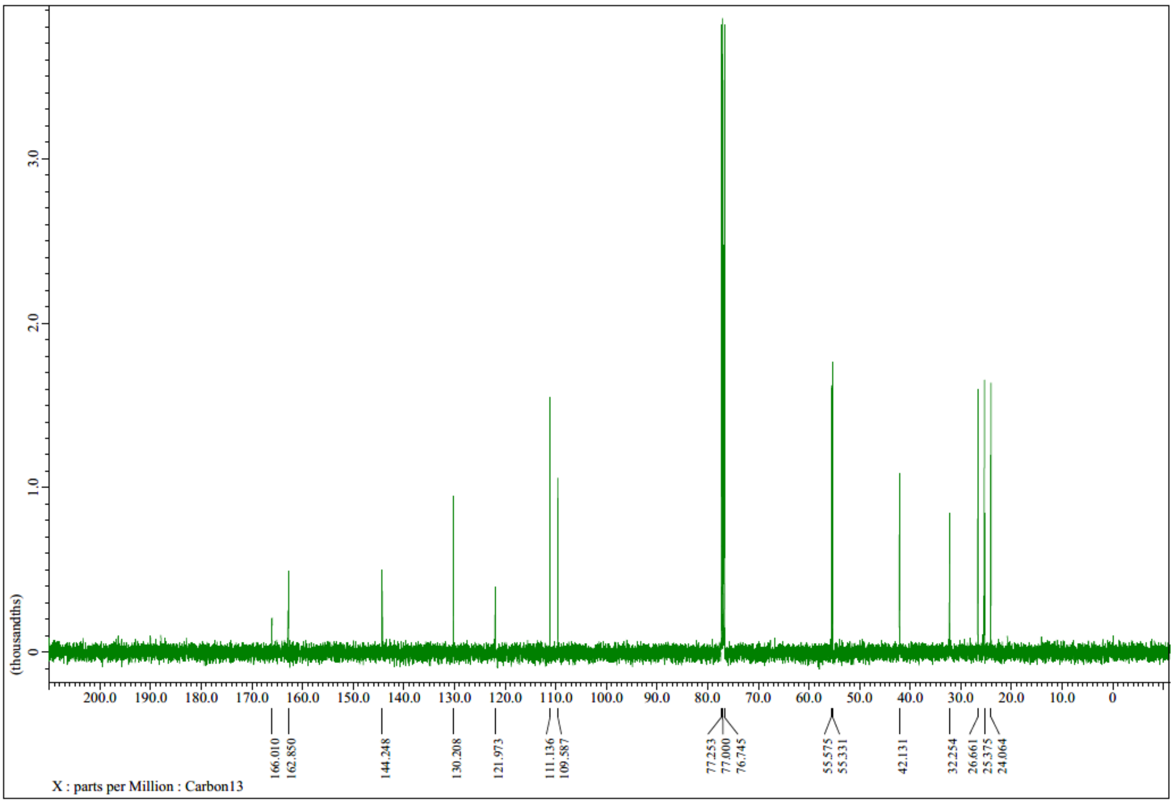
(thousandths)
X : parts per Million : Carbon13
166.010
162.850
144.248
130.208
121.973
111.136
109.587
77.253
77.000
76.745
55.575
55.331
42.131
32.254
26.661
25.375
24.064

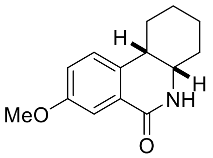
H
H
NH
MeO
O

cis-**5d**

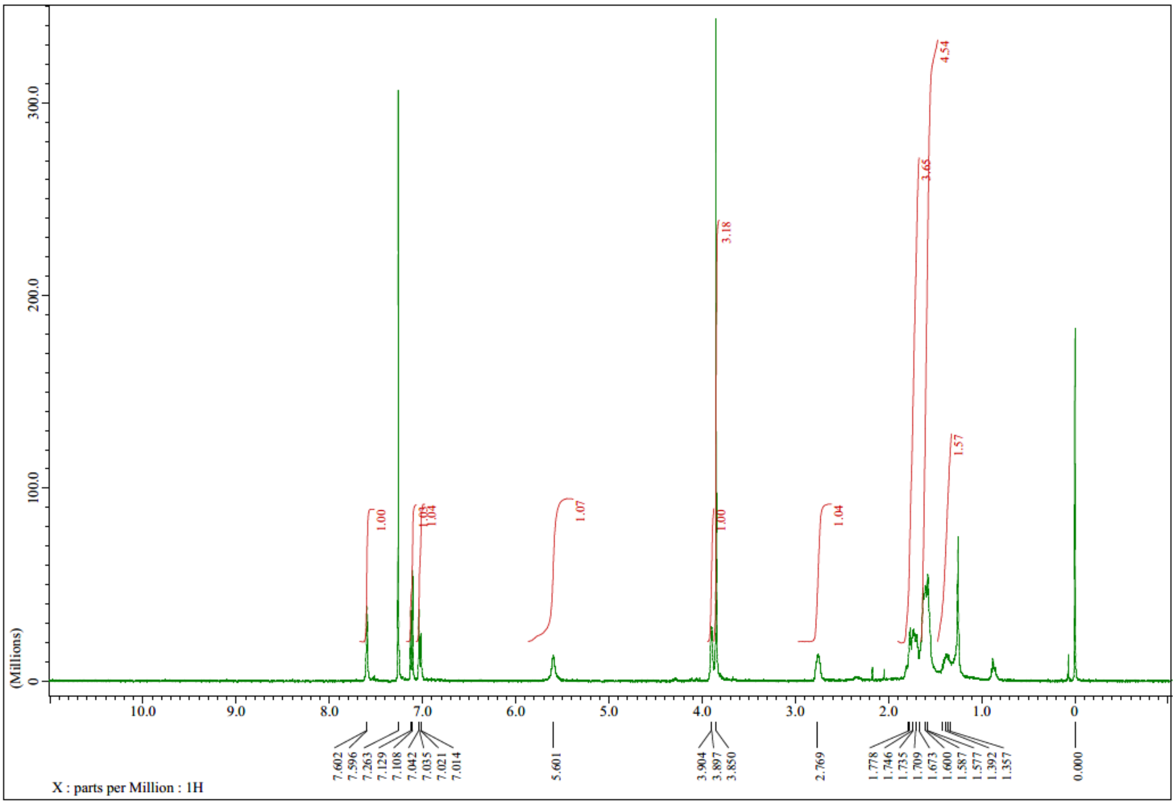
(Millions)
300.0
200.0
100.0
0
1.00
1.03
1.04
1.07
3.18
1.00
1.04
3.65
4.54
1.57
10.0 9.0 8.0 7.0 6.0 5.0 4.0 3.0 2.0 1.0 0
7.602
7.596
7.263
7.129
7.108
7.042
7.035
7.021
7.014
5.601
3.904
3.897
3.850
2.769
1.778
1.746
1.735
1.709
1.673
1.600
1.587
1.577
1.392
1.357
0.000
X : parts per Million : 1H

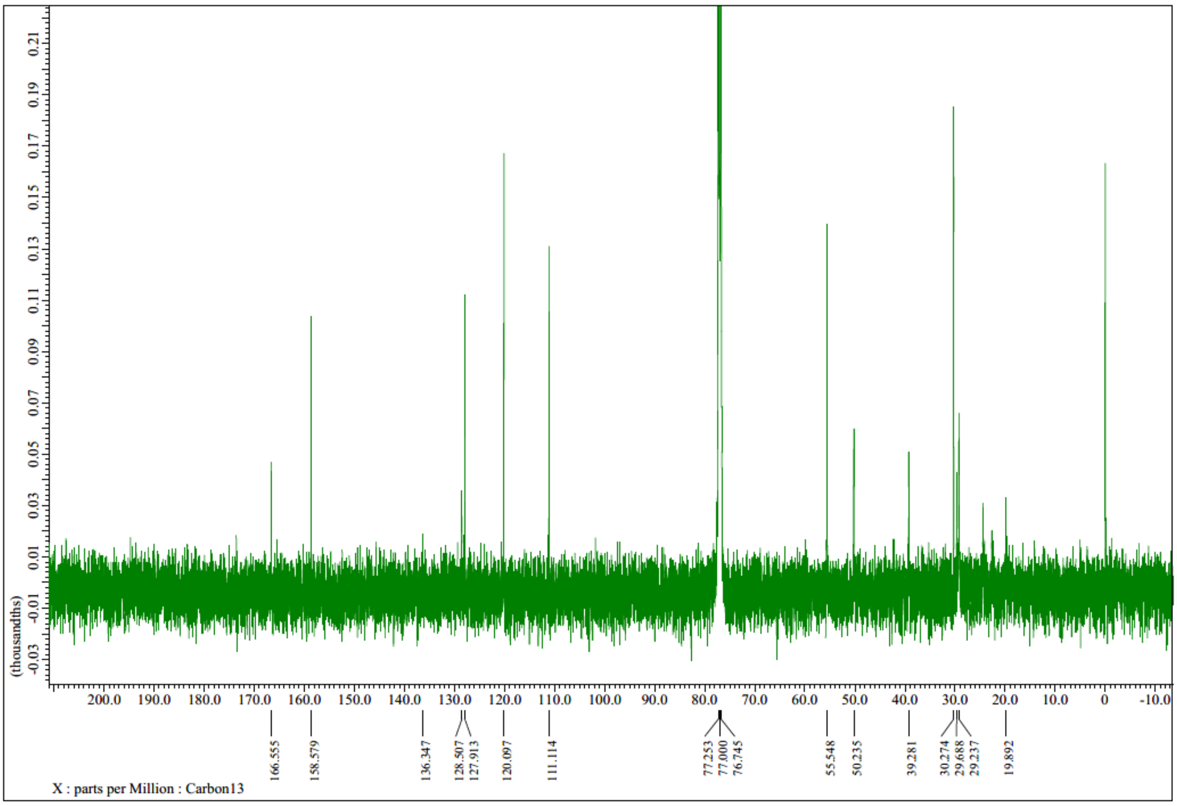
(thousandths)
0.21
0.19
0.17
0.15
0.13
0.11
0.09
0.07
0.05
0.03
0.01
-0.01
-0.03
200.0 190.0 180.0 170.0 160.0 150.0 140.0 130.0 120.0 110.0 100.0 90.0 80.0 70.0 60.0 50.0 40.0 30.0 20.0 10.0 0 -10.0
166.555
158.579
136.347
128.507
127.913
120.097
111.114
77.253
77.000
76.745
55.548
50.235
39.281
30.274
29.688
29.237
19.892
X : parts per Million : Carbon13

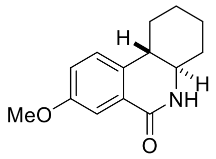
H
H
NH
MeO
O

trans-**5d**

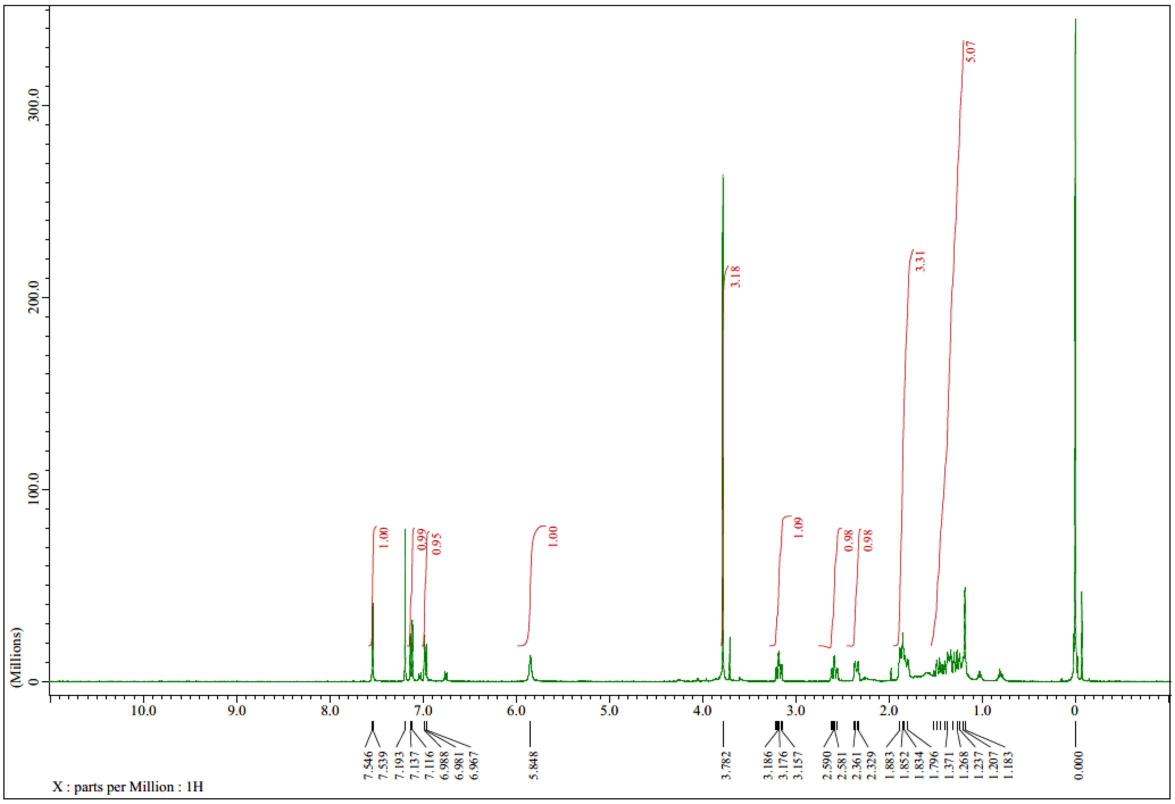
(Millions)
X : parts per Million : 1H
7.546
7.539
7.193
7.137
7.116
6.988
6.981
6.967
5.848
3.782
3.186
3.176
3.157
2.590
2.581
2.361
2.329
1.883
1.852
1.834
1.796
1.371
1.268
1.237
1.207
1.183
0.000
1.00
0.99
0.95
1.00
3.18
1.09
0.98
0.98
3.31
5.07

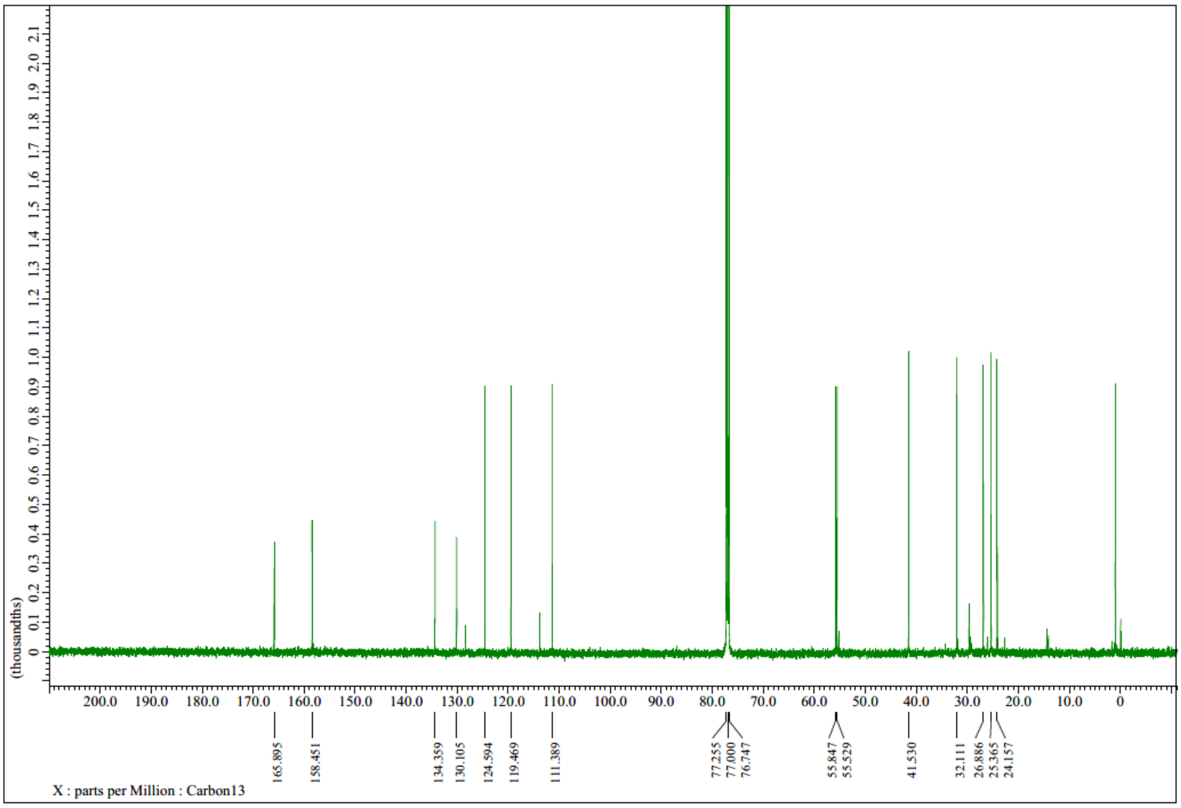
(thousandths)
X : parts per Million : Carbon13
165.895
158.451
134.359
130.105
124.594
119.469
111.389
77.255
77.000
76.747
55.847
55.529
41.530
32.111
26.886
25.365
24.157

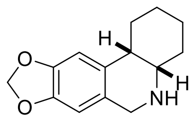
O
O
H
H
NH

cis-**6**

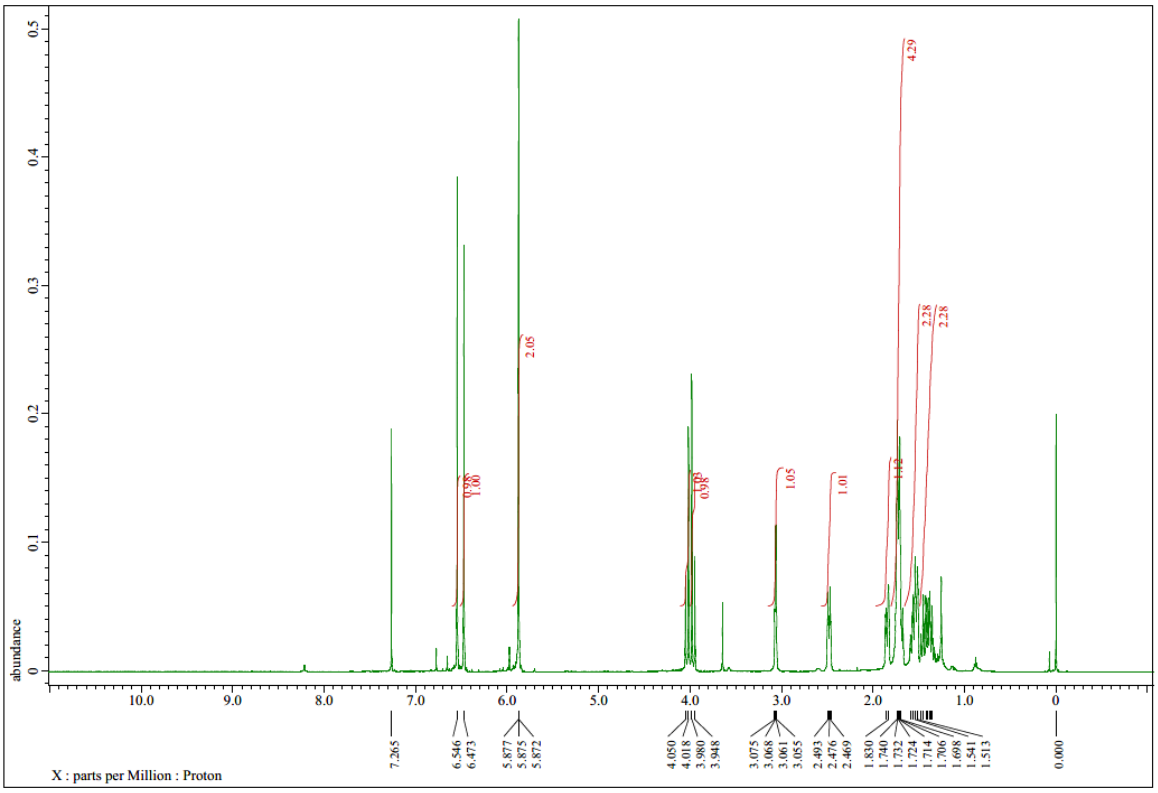
abundance
X : parts per Million : Proton
7.265
6.546
6.473
5.877
5.875
5.872
4.050
4.018
3.980
3.948
3.075
3.068
3.061
3.055
2.493
2.476
2.469
1.830
1.740
1.732
1.724
1.714
1.706
1.698
1.541
1.513
0.000

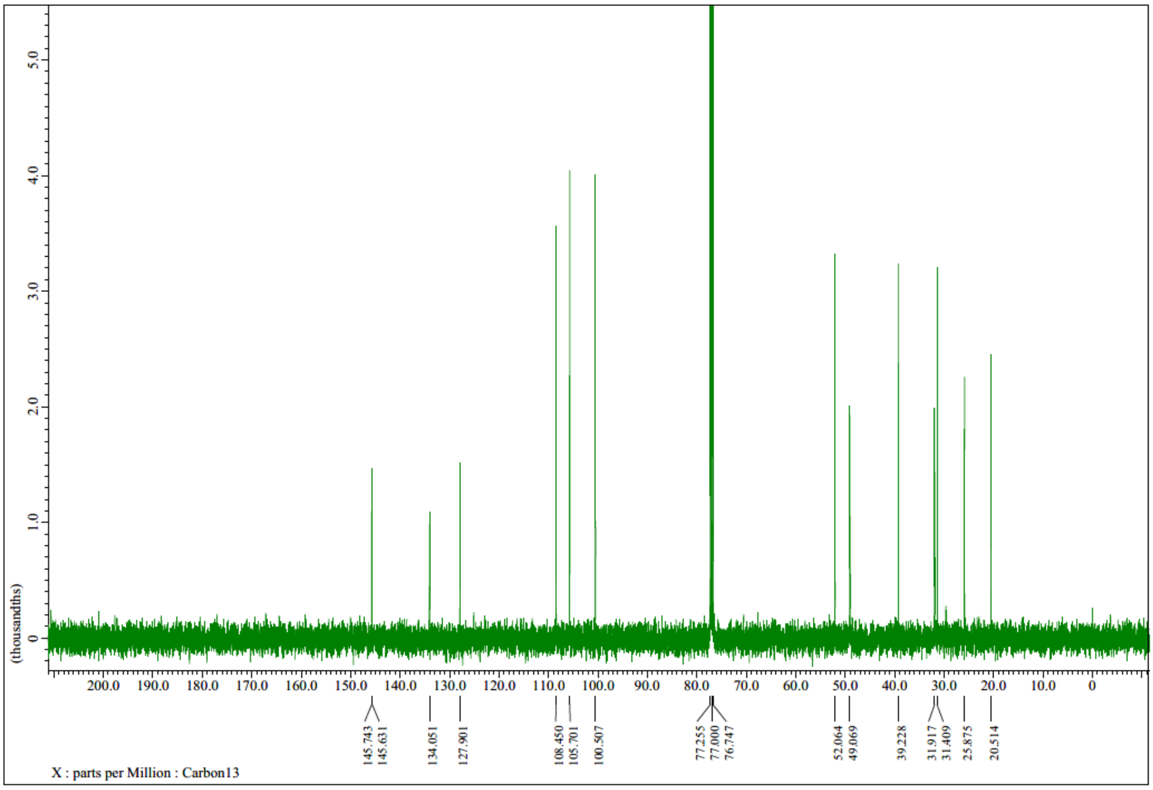
(thousandths)
X : parts per Million : Carbon13
145.743
145.631
134.051
127.901
108.450
105.701
100.507
77.255
77.000
76.747
52.064
49.069
39.228
31.917
31.409
25.875
20.514

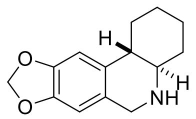
O
O
H
H
NH

trans-**6**

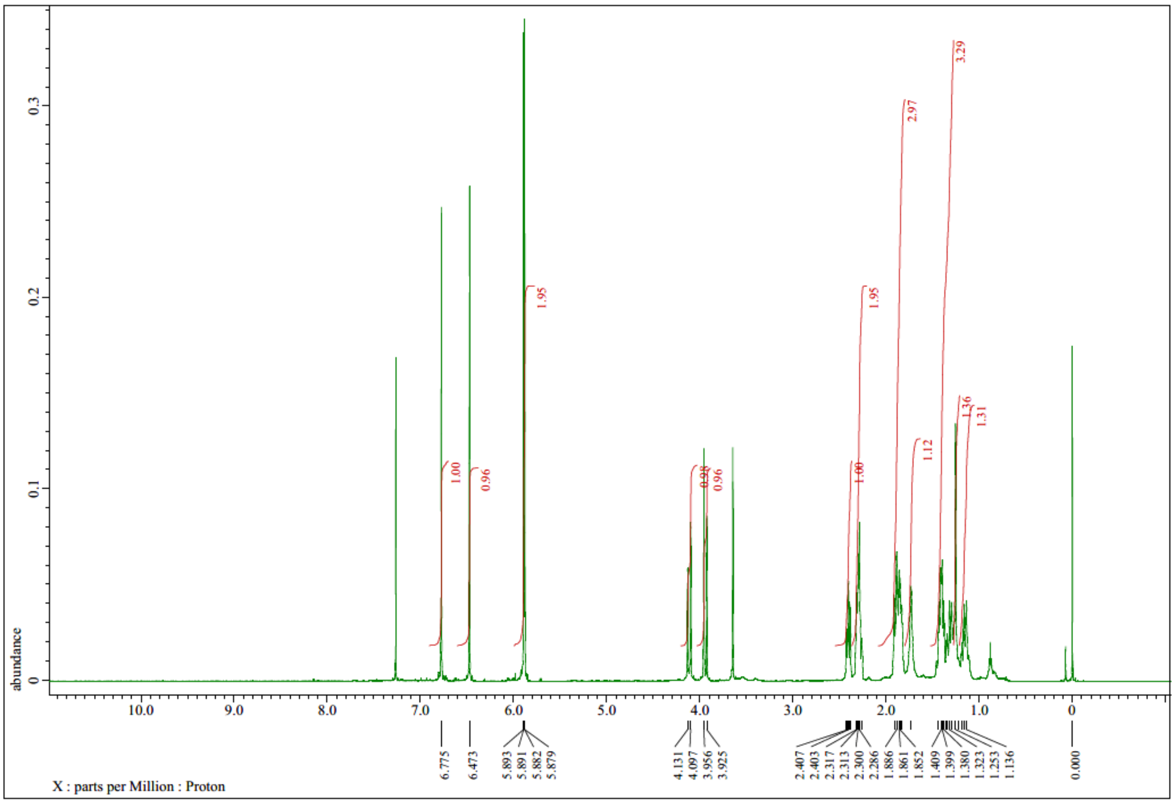
abundance
X : parts per Million : Proton
1.00
0.96
1.95
0.98
0.96
1.00
1.95
2.97
1.12
1.36
1.31
3.29
6.775
6.473
5.893
5.891
5.882
5.879
4.131
4.097
3.956
3.925
2.407
2.403
2.317
2.313
2.300
2.286
1.886
1.861
1.852
1.409
1.399
1.380
1.323
1.253
1.136
0.000

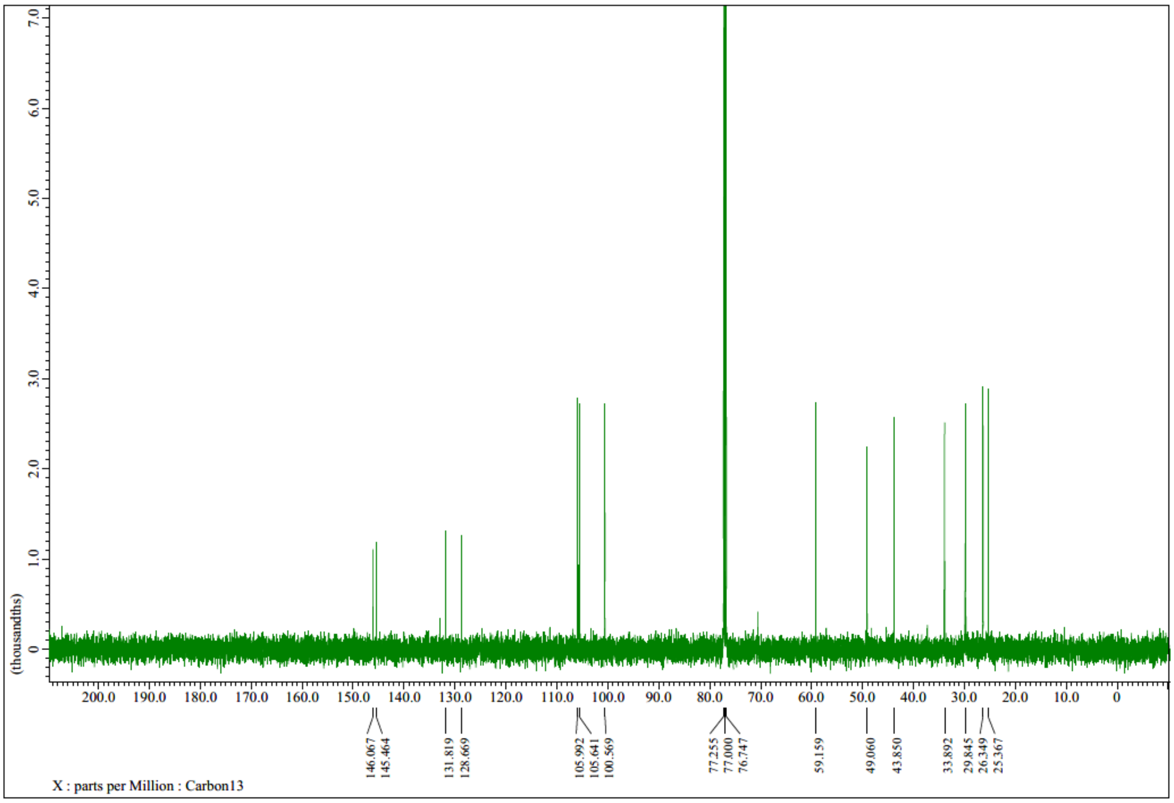
(thousandths)
X : parts per Million : Carbon13
146.067
145.464
131.819
128.669
105.992
105.641
100.569
77.255
77.000
76.747
59.159
49.060
43.850
33.892
29.845
26.349
25.367
